# Benchmarking atlas-level data integration in single-cell genomics

**DOI:** 10.1038/s41592-021-01336-8

**Published:** 2021-12-23

**Authors:** Malte D. Luecken, M. Büttner, K. Chaichoompu, A. Danese, M. Interlandi, M. F. Mueller, D. C. Strobl, L. Zappia, M. Dugas, M. Colomé-Tatché, Fabian J. Theis

**Affiliations:** 1 Institute of Computational Biology, Helmholtz Zentrum München, German Research Center for Environmental Health, Neuherberg, Germany; 2 Institute of Medical Informatics, University of Münster, Münster, Germany; 3 Department of Mathematics, Technische Universität München, Garching bei München, München, Germany; 4 Institute of Medical Informatics, Heidelberg University Hospital, Heidelberg, Germany; 5 TUM School of Life Sciences Weihenstephan, Technical University of Munich, Freising, Germany; 6 Biomedical Center (BMC), Physiological Chemistry, Faculty of Medicine, Ludwig Maximilian University of Munich, Planegg-Martinsried, Germany

**Keywords:** Machine learning, Data integration, Software, Transcriptomics

## Abstract

Single-cell atlases often include samples that span locations, laboratories and conditions, leading to complex, nested batch effects in data. Thus, joint analysis of atlas datasets requires reliable data integration. To guide integration method choice, we benchmarked 68 method and preprocessing combinations on 85 batches of gene expression, chromatin accessibility and simulation data from 23 publications, altogether representing >1.2 million cells distributed in 13 atlas-level integration tasks. We evaluated methods according to scalability, usability and their ability to remove batch effects while retaining biological variation using 14 evaluation metrics. We show that highly variable gene selection improves the performance of data integration methods, whereas scaling pushes methods to prioritize batch removal over conservation of biological variation. Overall, scANVI, Scanorama, scVI and scGen perform well, particularly on complex integration tasks, while single-cell ATAC-sequencing integration performance is strongly affected by choice of feature space. Our freely available Python module and benchmarking pipeline can identify optimal data integration methods for new data, benchmark new methods and improve method development.

## Abstract (web summary)

This benchmarking study compares 16 methods for integrating complex single-cell RNA and ATAC datasets and provides a guide to method choice.

## Main

The complexity of single-cell omics datasets is increasing. Current datasets often include many samples^1^, generated across multiple conditions^2^, with the involvement of multiple laboratories^3^. Such complexity, which is common in reference atlas initiatives such as the Human Cell Atlas^4^, creates inevitable batch effects. Therefore, the development of data integration methods that overcome the complex, nonlinear, nested batch effects in these data has become a priority: a grand challenge in single-cell RNA-seq data analysis^5,6^.

Batch effects represent unwanted technical variation in data that result from handing cells in distinct batches. These effects can arise from variations in sequencing depth, sequencing lanes, read length, plates or flow cells, protocol, experimental laboratories, sample acquisition and handling, sample composition, reagents or media and/or sampling time. Furthermore, biological factors such as tissues, spatial locations, species, time points or inter-individual variation can also be regarded as a batch effect.

A single-cell data integration method aims to combine high-throughput sequencing datasets or samples to produce a self-consistent version of the data for downstream analysis^7^. Batch-integrated cellular profiles are represented as an integrated graph, a joint embedding or a corrected feature matrix.

Currently, at least 49 integration methods for scRNA-seq data are available^8^ (as of November 2020, Supplementary Table 1). In the absence of objective metrics, subjective opinions based on visualizations of integrated data will determine method evaluation. Benchmarking integration methods facilitates this process to provide an unbiased guide to method choice.

Previous studies on benchmarking methods for data integration have focused on the simpler problem of batch effect removal^7^ in scRNA-seq^9–11^. These studies benchmarked methods on simple integration tasks with low batch or biological complexity and did not compare different output options such as corrected features or joint embeddings, finding that ComBat^11^ or the linear, principal component analysis (PCA)-based, Harmony method^9^ outperformed more complex, nonlinear, methods.

Here, we present a benchmarking study of data integration methods in complex integration tasks, such as tissue or organ atlases. Specifically, we benchmarked 16 popular data integration tools on 13 data integration tasks consisting of up to 23 batches and 1 million cells, for both scRNA- and single-cell ATAC-sequencing (scRNA-seq and scATAC-seq) data. We selected 12 single-cell data integration tools: mutual nearest neighbors (MNN)^12^ and its extension FastMNN^12^, Seurat v3 (CCA and RPCA)^13^, scVI^14^ and its extension to an annotation framework (scANVI^15^), Scanorama^16^, batch-balanced *k* nearest neighbors (BBKNN)^17^, LIGER^18^, Conos^19^, SAUCIE^20^ and Harmony^21^; one bulk data integration tool (ComBat^22^); a method for clustering with batch removal (DESC^23^) and two perturbation modeling tools developed previously by one of the authors (trVAE^24^ and scGen^25^). Moreover, we use 14 metrics to evaluate the integration methods on their ability to remove batch effects while conserving biological variation. We focus in particular on assessing the conservation of biological variation beyond cell identity labels via new integration metrics on trajectories or cell-cycle variation. We find that Scanorama and scVI perform well, particularly on complex integration tasks. If cell annotations are available, scGen and scANVI outperform most other methods across tasks, and Harmony and LIGER are effective for scATAC-seq data integration on window and peak feature spaces.

## Results

### Single-cell integration benchmarking (scIB)

**Fig. 1:**
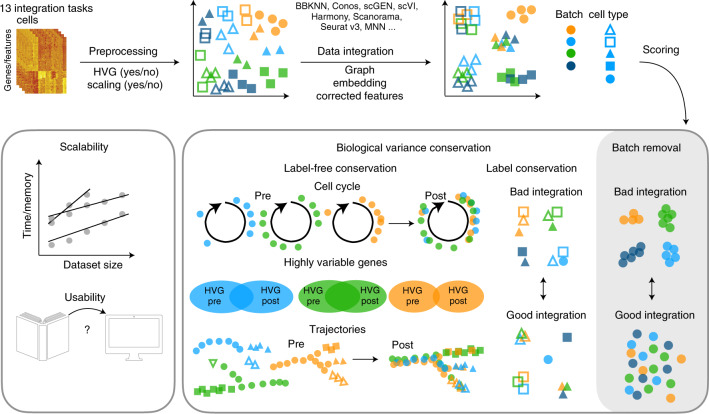
Design of single-cell integration benchmarking (scIB). Schematic diagram of the benchmarking workflow. Here, 16 data integration methods with four preprocessing decisions are tested on 13 integration tasks. Integration results are evaluated using 14 metrics that assess batch removal, conservation of biological variance from cell identity labels (label conservation) and conservation of biological variance beyond labels (label-free conservation). The scalability and usability of the methods are also evaluated.

We benchmarked 16 popular data integration methods on 13 preprocessed integration tasks: two simulation tasks, five scRNA-seq tasks and six scATAC-seq tasks (Fig. 1). Each task posed a unique challenge (for example, nested batch effects caused by protocols and donors, batch effects in a different data modality and scalability up to 1 million cells) that revolved around integrating data on a particular biological system from multiple laboratories (Table 1). Our simulation tasks allowed us to assess the integration methods in a setting where the nature of the batch effect could be determined and the ground truth is known. In real data, we predetermined the ground truth by preprocessing and annotating data from 23 publications separately for each batch (Methods).

**Table 1:** Integration tasks for benchmarking

Integration task	Cell number	Batches	Tested features
Pancreas	16,382	9 batches	Widely used test data, protocols
Lung	32,472	16 donors	Human variation, protocols, spatial locations, high resolution subtypes, laboratories
Immune (human)	33,506	10 donors	Tissues, laboratories, similar cell types
Immune (human and mouse)	97,952	23 samples	Tissues, laboratories, similar cell types, species
Mouse brain (RNA)	978,734	4 datasets	Large dataset, spatial locations, nucleus versus cell, protocols
Mouse brain small (ATAC, 3 tasks: windows, peaks, gene activity)	10,761, 11,597, 11,270	3 datasets	Different modality, laboratories, technologies and feature spaces
Mouse brain large (ATAC, 3 tasks: windows, peaks gene activity)	84,813	11 samples	Different samples from 3 unbalanced datasets; different modality, laboratories, technologies and feature spaces
Simulation 1	12,097	6 batches	Variation in cellular compositions
Simulation 2	19,318	16 batches	Nested batch effects, composition variation

Overview of the tasks used to benchmark data integration methods. The tested feature describes the unique challenge presented by the integration task. Donor refers to human individuals, sample is used when mice are involved and batches is the general term that includes dataset and sample batches. The six ATAC tasks are summarized in two entries.

Each integration method was evaluated with regards to accuracy, usability and scalability (Methods). Integration accuracy was evaluated using 14 performance metrics divided into two categories: removal of batch effects and conservation of biological variance (Fig. 1). Batch effect removal per cell identity label was measured via the *k*-nearest-neighbor batch effect test (kBET)^11^, *k-*nearest-neighbor (*k*NN) graph connectivity and the average silhouette width (ASW)^11^ across batches. Independently of cell identity labels, we further measured batch removal using the graph integration local inverse Simpson’s Index (graph iLISI, extended from iLISI^21^) and PCA regression^11^. Conservation of biological variation in single-cell data can be captured at the scale of cell identity labels (label conservation) and beyond this level of annotation (label-free conservation). Therefore, we used both classical label conservation metrics, which assess local neighborhoods (graph cLISI, extended from cLISI^21^), global cluster matching (Adjusted Rand Index (ARI)^26^, normalized mutual information (NMI)^27^) and relative distances (cell-type ASW) as well as two new metrics evaluating rare cell identity annotations (isolated label scores) and three new label-free conservation metrics: (1) cell-cycle variance conservation, (2) overlaps of highly variable genes (HVGs) per batch before and after integration and (3) conservation of trajectories (Methods).

Two central challenges to benchmarking data integration methods are: (1) the diversity of output formats^28^, and (2) the inconsistent requirement on data preprocessing before integration. We addressed these challenges in three ways. First, all integration outputs were treated as separate integration runs. For example, Scanorama outputs both corrected expression matrices and embeddings; these are evaluated separately as Scanorama gene and Scanorama embedding. Some methods output more than an integrated graph, joint embedding or corrected feature space; for example, scANVI outputs predicted labels where these are not provided, and DESC outputs a clustering of the data. These outputs are explicitly not evaluated in our study. Second, we developed new extensions to kBET and LISI scores that work on graph-based outputs, joint embeddings and corrected data matrices in a consistent manner (Supplementary Notes 1 and 2). Thus, multiple metrics could be computed for each category of batch effect removal, label conservation and label-free conservation (Supplementary Table 2). Overall accuracy scores were computed by taking the weighted mean of all metrics computed for an integration run, with a 40/60 weighting of batch effect removal to biological variance conservation (bio-conservation) irrespective of the number of metrics computed. Third, we also included preprocessing decisions in our benchmark: each integration method was run with and without scaling and HVG selection. We considered that some methods cannot accept scaled input data (LIGER, trVAE, scVI and scANVI) and that others require cell-type labels as input (scGen and scANVI). Thus, we tested up to 68 data integration setups per integration task, resulting in 590 attempted integration runs. All performance metrics, integration methods with parameterizations and preprocessing functions have been made available in our scIB Python module. Furthermore, the generated outputs are visible on our scIB website and our workflow is provided as a reproducible Snakemake^29^ pipeline to allow users to test and evaluate data integration methods in their own setting (Code availability).

### Benchmarking data integration: the human immune cell task

To demonstrate our evaluation pipeline, we first focus on the human immune cell integration task (Supplementary Note 3). This task comprises ten batches representing donors from five datasets with cells from peripheral blood and bone marrow on which Scanorama (embedding), FastMNN (embedding), scANVI and Harmony performed best.

**Fig. 2:**
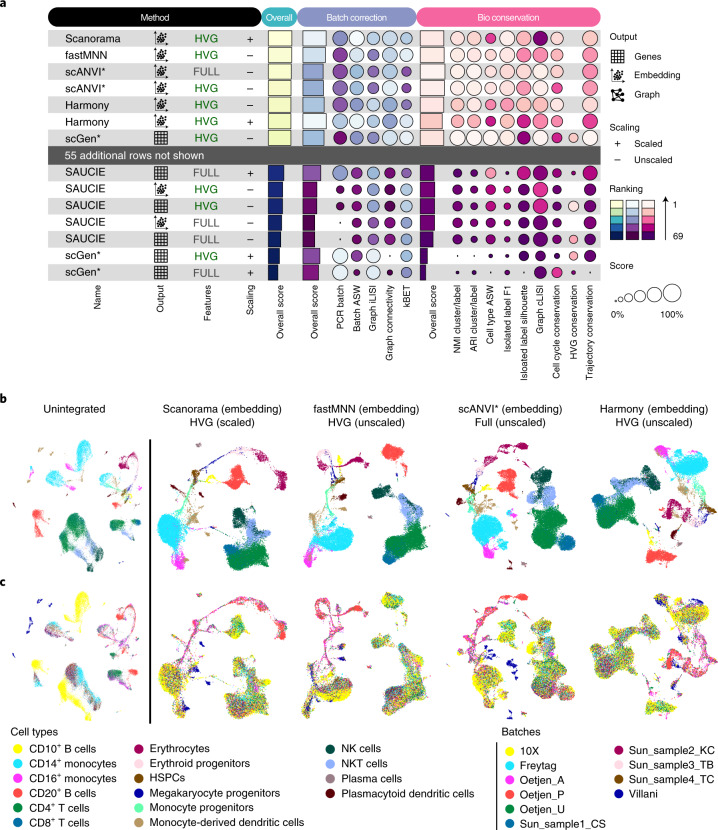
Benchmarking results for the human immune cell task. **a**, Overview of top and bottom ranked methods by overall score for the human immune cell task. Metrics are divided into batch correction (blue) and bio-conservation (pink) categories. Overall scores are computed using a 40/60 weighted mean of these category scores (see Methods for further visualization details and Supplementary Fig. 2 for the full plot). **b**,**c**, Visualization of the four best performers on the human immune cell integration task colored by cell identity (**b**) and batch annotation (**c**). The plots show uniform manifold approximation and projection layouts for the unintegrated data (left) and the top four performers (right).

Comparing the metric results (Fig. 2a) to the integrated data plots (Fig. 2b,c) shows a consistent picture: all high-performing methods successfully removed batch effects between individuals and platforms while conserving biological variation at the cell-type and subtype levels. kBET and iLISI batch removal metrics separate the top performers: Scanorama scored higher in these metrics as it integrated the Villani (Smart-seq2), Oetjen et al.^30^ batches and 10X batches better compared to scANVI and FastMNN, which showed residual 10X batch structure in CD14^+^ monocytes. Additionally, scANVI exhibited residual Oetjen batch structure in erythrocytes and separated the Villani batch. This scANVI behavior is expected given the method is not designed for full-length Smart-seq2 data. Further, Harmony exhibited the lowest isolated label F1 bio-conservation score among top performers. The isolated labels in this task were CD10^+^ B cells, erythroid progenitors, erythrocytes and megakaryocyte progenitors, which are exclusive to the Oetjen et al.^30^ batches. While Harmony kept each isolated cell label together, it overlapped these populations.

**Extended Data Fig. 1:**
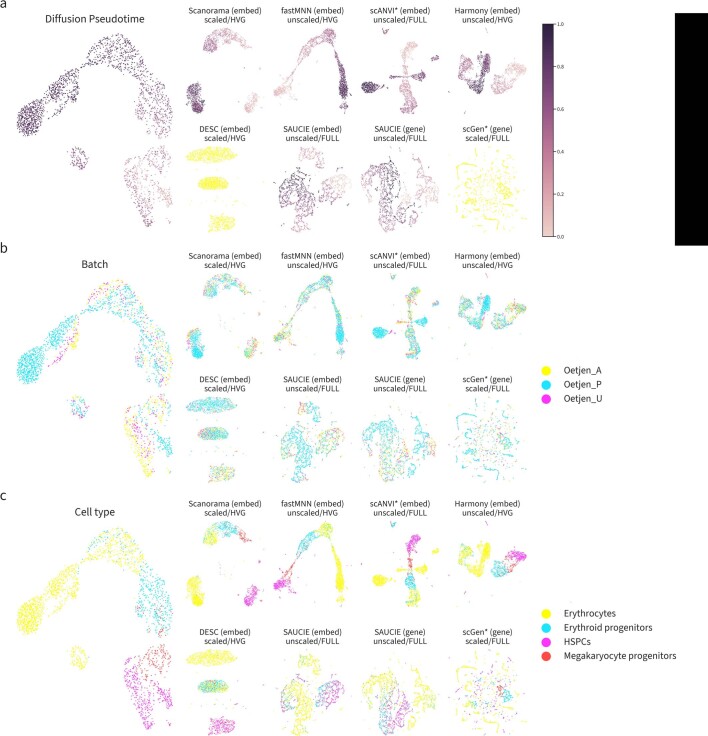
Trajectories of the best and worst performers on the immune cell human integration task ordered by overall score on the set of cells belonging to the erythrocyte lineage. UMAP plots for the unintegrated data (left), the top 4 performers (upper rows a, b and c), and the worst 4 performers (lower rows a and b). Plots are colored by (**a**) diffusion pseudotime, (**b**) batch labels, and (**c**) cell identity annotations.

**Extended Data Fig. 2:**
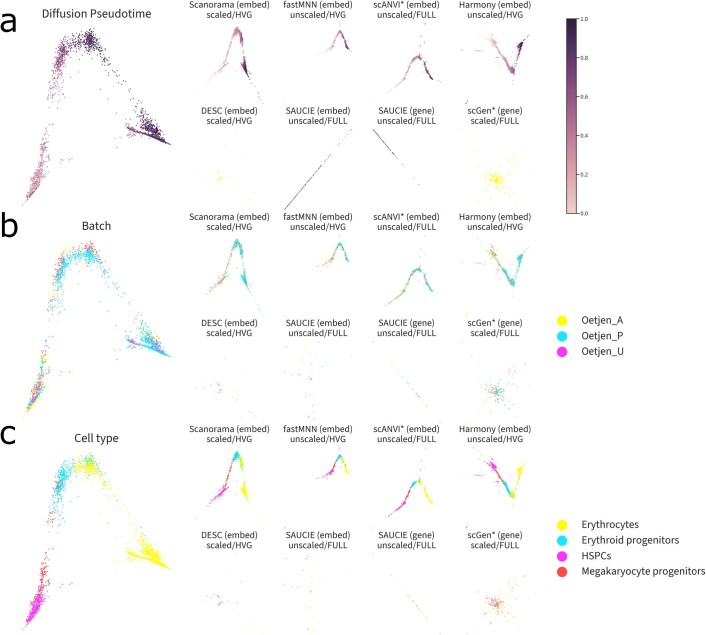
Diffusion maps of diffusion pseudotime (dpt) trajectories on integrated immune cell human data of the best and worst performers ordered by overall score. Diffusion maps of erythrocyte lineage cells of the 4 best (upper rows a, b and c) and 4 worst (lower rows a, b and c) integration methods, ordered by the overall score. Plots are colored by (**a**) diffusion pseudotime, (**b**) batch labels, and (**c**) cell identity annotations. In cases where it wasn’t possible to compute a trajectory due to disconnected clusters, all cells are colored yellow in (a).

**Extended Data Fig. 3:**
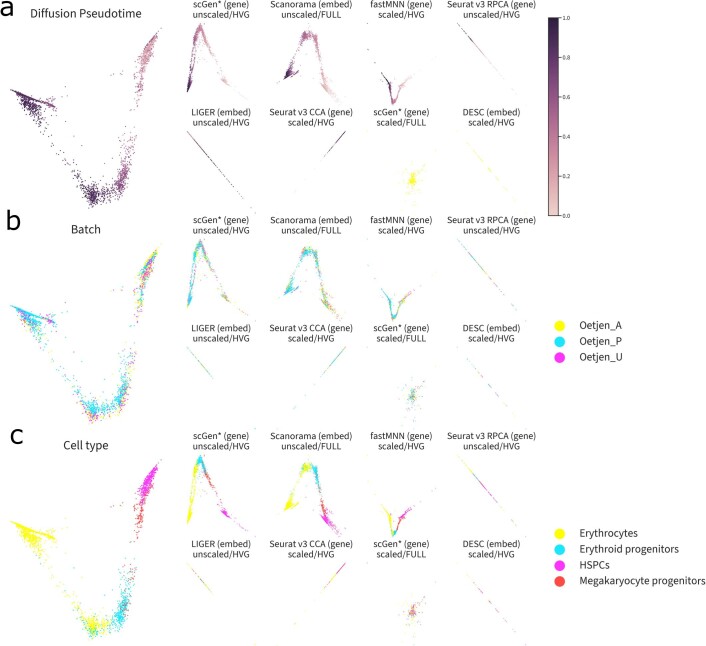
Diffusion maps of diffusion pseudotime (dpt) trajectories on integrated immune cell human data of the best and worst performers ordered by trajectory score. Diffusion maps of erythrocyte lineage cells of the 4 best (upper row a, b and c) and 4 worst (lower row a, b and c) integration methods, ordered by the overall score. Plots are colored by (**a**) diffusion pseudotime, (**b**) batch labels, and (**c**) cell identity annotations. In cases where it wasn’t possible to compute a trajectory due to disconnected clusters, all cells are colored yellow in (a).

We also focused on the conservation of trajectories. In this integration task, we assessed erythrocyte development from hematopoietic stem and progenitor cells via megakaryocyte progenitors and erythroid progenitors to erythrocytes (Extended Data Figs. 1–3 and Supplementary Fig. 1). All of the top-performing methods exhibited high trajectory conservation scores, whereas DESC (on scaled/HVG data), scGen (on scaled/full feature data) and Seurat v3 CCA (on scaled/HVG data), produced poor conservation of this trajectory due to overclustering (DESC), merging of cell types (Seurat v3 CCA) or lack of relevant biological latent structure (scGen). Notably, Seurat v3 CCA introduced an unexpected branching structure into the trajectory in diffusion map space (Extended Data Fig. 3).

### Balancing batch removal and biological variance conservation

Considering the results of the five scRNA-seq and two simulation tasks (Supplementary Note 3 and Supplementary Figs. 2–15), we found that the varying complexity of tasks affects the ranking of integration methods: while Seurat v3 and Harmony perform well on simpler real data tasks and some simulations, Scanorama and scVI performed particularly well on more complex real data. Only scGen and scANVI, which additionally use cell-type information to improve integration results, performed well across tasks. In general, the simulations contained less nuanced biological variation but exhibited clearly defined, often strong, batch effects. Specifically, simulation task 1 posed little difficulty to most methods independent of preprocessing decisions (Supplementary Figs. 4 and 11). Similarly, the widely used pancreas integration task contains distinct cell-type variation and batch effects; thus, even methods that perform poorly overall, performed well on this task (Supplementary Figs. 6 and 13 and Supplementary Note 3).

**Fig. 3:**
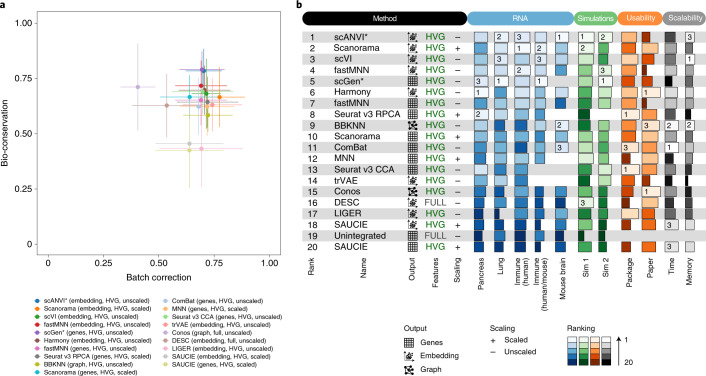
Overview of benchmarking results on all RNA integration tasks and simulations, including usability and scalability results. **a**, Scatter plot of the mean overall batch correction score against mean overall bio-conservation score for the selected methods on RNA tasks. Error bars indicate the standard error across tasks on which the methods ran. **b**, The overall scores for the best performing method, preprocessing and output combinations on each task as well as their usability and scalability. Methods that failed to run for a particular task were assigned the unintegrated ranking for that task. An asterisk after the method name (scANVI and scGen) indicates that, in addition, cell identity information was passed to this method. For ComBat and MNN, usability and scalability scores corresponding to the Python implementation of the methods are reported (Scanpy and mnnpy, respectively).

**Extended Data Fig. 4:**
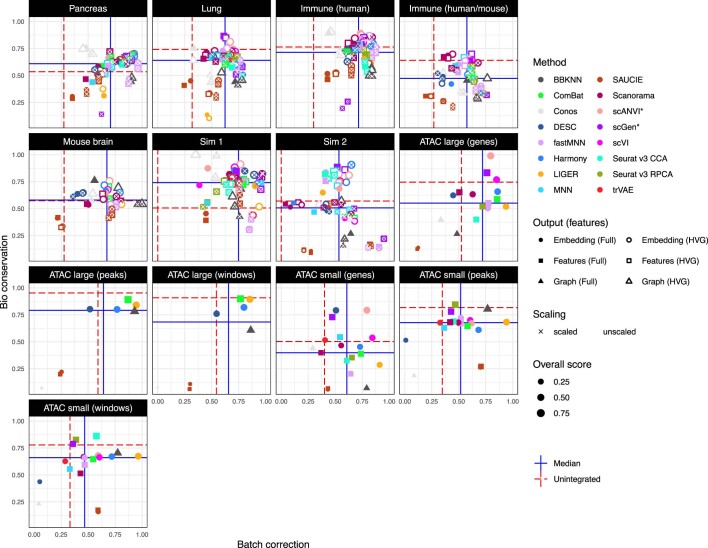
Scatter plots summarizing integration performance on all tasks. Overall batch correction score (x-axis) versus overall bio-conservation score (y-axis). Each point is an individual integration run. Point color indicates method, size the overall score and shape the output type (embedding, features, graph). Filled points use the full feature set while unfilled points use selected highly variable genes. Points marked with a cross use scaled features. Horizontal and vertical lines indicate reference points. Red dashed lines show performance calculated on the unintegrated dataset and solid blue lines the median performance across methods for each dataset.

Particularly in more complex integration tasks, we observed a tradeoff between batch effect removal and bio-conservation (Fig. 3a and Supplementary Data 1). While methods such as SAUCIE, LIGER, BBKNN and Seurat v3 tend to favor the removal of batch effects over conservation of biological variation, DESC and Conos make the opposite choice, and Scanorama, scVI and FastMNN (gene) balance these two objectives. Other methods strike different balances per task (Extended Data Fig. 4). This tradeoff is particularly noticeable where biological and batch effects overlap. For example, in the lung task, three datasets sample two distinct spatial locations (airway and parenchyma). Particular cell types such as endothelial cells perform different functions in these locations (for example, gas exchange in the parenchyma). While Seurat v3 and BBKNN integrated across the locations to merge these cells, providing a broad cell-type overview, Scanorama preserved the spatial variation in endothelial cells and other cell types that have functional differences across locations (Supplementary Note 3). Methods that use cell identity information (scGen and scANVI) must be considered separately in this tradeoff. These methods preserved biological variation most strongly. Yet, performance depended on the resolution of the cell identity labels: if specific biological variation is not encoded in cell identity labels (for example, spatial location in lung endothelial cells), scGen in particular will remove biological variation confounded with batch effects. However, if this variation is encoded (for example, neutrophil states in the lung), scGen and scANVI are the only methods that are able to preserve cell state differences that are each present only in a single batch.

The most challenging batch effects across the integration tasks were due to species, sampling locations and single-nucleus versus single-cell data. These batch effect contributors can also be interpreted as biological signals rather than technical noise. While the top-performing methods across integration tasks were largely unable to integrate across these effects (unless they received cell identity annotations; Supplementary Figs. 10, 14 and 15), LIGER, BBKNN and Seurat v3 RPCA were successful. These integration results, however, often remove biological variation along with the batch effect, showcasing the aforementioned tradeoff between batch removal and bio-conservation. This effect was particularly noticeable for the immune cell human and mouse and mouse brain tasks. For immune cells, only LIGER, BBKNN, Seurat v3 RPCA and scGen integrated across species, but removed nuanced biological variation to varying degrees. While LIGER and BBKNN retained only broad cell-type variation on integration, Seurat v3 RPCA conserved more distinct cell identities, but merged neutrophils with various progenitor populations. Even scGen, the top-performing integration output for this task, separated CD4^+^ and CD8^+^ T cells as well as erythrocyte progenitors and erythrocytes, while otherwise integrating mouse and human cells as expected (Supplementary Figs. 10 and 16 and Supplementary Note 3). When subtle cell states were not annotated in the data, we found that Scanorama, scANVI, scVI and Harmony could, however, integrate across strong batch effects from single nuclei and single cells while retaining biological variation on spatial locations and rare cell types (see the mouse brain task in Supplementary Note 3).

Methods that favor bio-conservation and output corrected expression matrices tended to better conserve cell state variation. Indeed, Scanorama (gene), ComBat and MNN consistently performed well at conserving cell-cycle variance and HVGs in the integrated data. Trajectory structure was slightly better conserved in the overall high-performing methods Scanorama, scGen and FastMNN, while poor performers were consistent across label-free metrics (Supplementary Figs. 1–3, Extended Data Fig. 3 and Supplementary Data 1). These methods placed cells in the expected order per batch and reconstructed the global trajectory structure on human immune cells (Supplementary Fig. 17). Additionally, scVI also performed well per batch in the human and mouse immune cell task, but did not generate an integrated continuum of states across human and mouse erythrocyte development in a single trajectory. Thus, while local trajectory structure was well-represented, the global trajectory structure was not robustly conserved (Supplementary Fig. 18). Even methods that did integrate datasets across species failed to reconstruct a consistent global trajectory structure (scGen and FastMNN) or poorly reflected the trajectory (LIGER). Overall, performing an integrated trajectory across species is challenging due to the strong species batch effect.

### Scaling shifts integration performance toward batch removal

Given the lack of best-practice for preprocessing raw data for data integration, we assessed whether integration methods perform better with HVG selection or scaling. Comparing the performance between integration runs that only differed in one preprocessing parameter, we found that HVG selection generally outperformed data integration of the full gene set across RNA and simulation tasks: for HVGs, 74% of comparisons had a higher overall score; 81% had better batch removal and 66% had better bio-conservation scores. Notable exceptions were trajectory and cell-cycle conservation scores, which tended to favor full feature integration runs.

We also found that whether or not a method performs better with previous scaling depends on the method of choice (Fig. 3b). Independent of the method, scaling resulted in higher batch removal scores (79% of comparisons) but lower bio-conservation (72% of comparisons). This observation is consistent with unscaled data performing better in our label-free conservation metrics. Although scaling aided integration across species in several methods, it did not lead to a better conservation of the trajectory, as even the best trajectory-conserving methods did not integrate perfectly across species in the human or mouse task.

### scANVI, Scanorama and scVI perform best for scRNA-seq

To evaluate overall performance of data integration methods across scRNA-seq and simulation tasks, methods can be ranked by their overall scores. Assuming there is a single, optimal way in which to run an integration method, we ranked methods by their top-performing preprocessing combination, which also indicated to users how best to run each integration method (Fig. 3b). The optimal preprocessing combinations of scGen, BBKNN, Scanorama, trVAE, scVI, scANVI, Seurat v3 CCA, FastMNN, Harmony and SAUCIE were consistent across tasks. Conos, which incorporates HVG selection and scaling within its method, performed slightly better on full feature input with scaling applied depending on the task. In comparison, the performance of MNN, ComBat and Seurat RPCA was better using HVG selection, with scaling having little effect on the output except a slightly improved performance in tasks with stronger batch effects. The performance of DESC and LIGER was not consistently better with a particular preprocessing combination, although preprocessing did affect their performance in some tasks.

Given that the complexity of a task affects the appropriateness of a method, we ranked methods excluding simulations, based only on real data tasks that better represent the challenges typically faced by analysts. Overall, the embeddings output by Scanorama, scANVI and scVI perform best, whereas SAUCIE and DESC perform poorly. These results are remarkably consistent across tasks for integrating real data. Of note, the corrected gene-expression matrix from scGen was ranked first most frequently, but it was penalized for not running on the 1 million mouse brain task in 4 days on a CPU. In contrast, Harmony ranked outside the top third of methods for more complex real data tasks, but was favorable for simulations and real data with less complex biological variation.

The methods with a higher level of abstraction tended to rank higher (in particular comparing Scanorama and FastMNN’s embeddings and corrected expression matrix output). In general, methods based on mutual nearest neighbors to find anchors between batches (for example, Scanorama and FastMNN) tended to perform well. Similarly, the deep learning-based methods that were aided by cell annotations produced integrated outputs that could integrate even across the strongest batch effects while conserving biological variation. Other autoencoder-based frameworks such as scVI and trVAE tended to perform better in tasks with more cells and complex batch structure. This was particularly noticeable for scVI, as trVAE did not scale to tasks of this size without graphical processing unit (GPU) hardware.

### scATAC-seq integration performance depends on feature space

Several of the benchmarked data integration methods have been used to integrate datasets across modalities^13,18^. With the growing availability of datasets, removing batch effects within scATAC-seq data is also becoming an application of interest. To test whether performance of scRNA-seq integration methods transfers to scATAC-seq data, we integrated three datasets of chromatin accessibility for mouse brain generated by different technologies (Methods).

In contrast to gene expression that is only defined on genes, chromatin accessibility is measured across the whole genome and can thus be represented in different feature spaces. To evaluate the impact of feature spaces on data integration, we preprocessed each of our scATAC-seq datasets into peaks, windows and genes (that is, gene activity; Methods). In each feature space, we considered two integration scenarios: the small integration scenario with three balanced batches (one batch from each dataset), and the large integration scenario with 11 nested batches from the three datasets of very different sizes (proportion of cells per dataset of 5%, 20% and 75%; Supplementary Data 2). To restrict the feature space, we used only the most variable peaks, windows or genes that overlap between datasets (Methods and Supplementary Note 3). In summary, we evaluated the performance of 19 data integration outputs on six scATAC-seq tasks (Table 1) using 11 evaluation metrics (feature-level metrics were not applicable, see Supplementary Table 2).

**Fig. 4:**
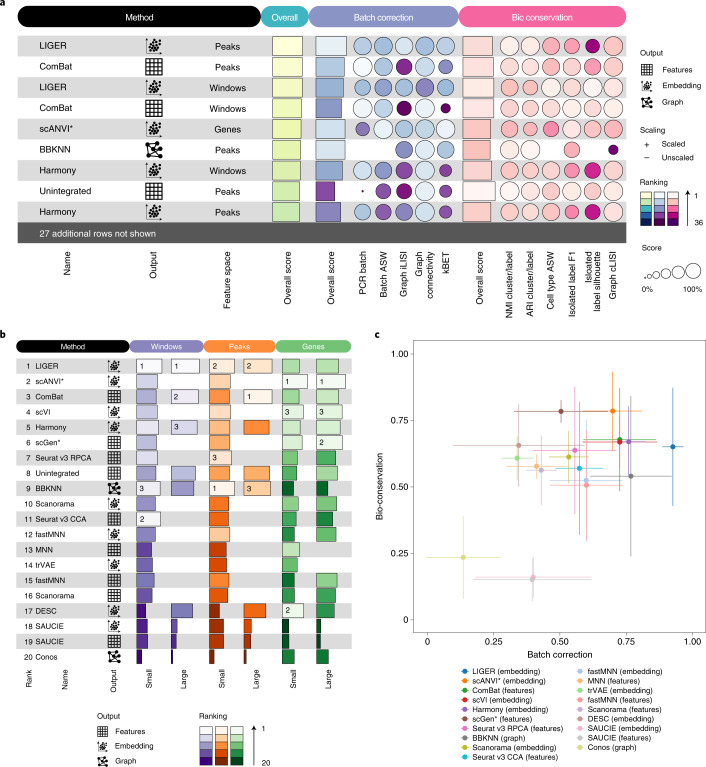
Benchmarking results for mouse brain ATAC tasks. **a**, Overview of top ranked methods by overall score for the combined large ATAC tasks. Metrics are divided into batch correction (blue) and bio-conservation (pink) categories. Overall scores are computed using a 40:60 weighted mean of these category scores (see Extended Data Fig. 5 for the full plot). **b**, The overall scores for the best performing methods on each task. Methods that failed to run for a particular task were assigned the unintegrated ranking for that task. Methods ranking below unintegrated are not suitable for integrating ATAC batches. **c**, Scatter plot summarizing integration performance on all ATAC tasks. The *x* axis shows the overall batch correction score and the *y* axis shows the overall bio-conservation score. Each point is an average value per method with the error bars indicating a standard deviation. All methods are indicated by different colors.

**Extended Data Fig. 5:**
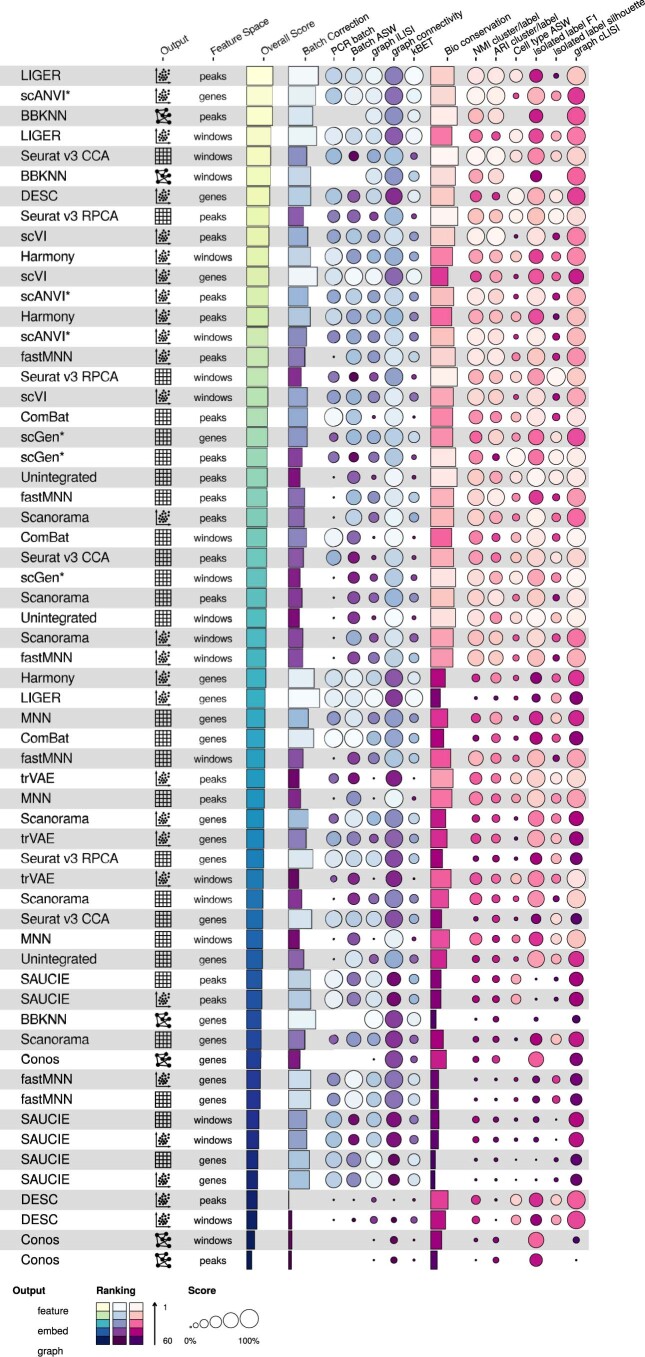
Benchmarking results for all small mouse brain tasks for all feature spaces based on scATAC-seq. Metrics are divided into batch correction (blue, purple) and bio conservation (pink) categories (see Methods for further visualization details). Overall scores are computed by a 40:60 weighted mean of these category scores. Methods that failed to run are omitted.

**Extended Data Fig. 6:**
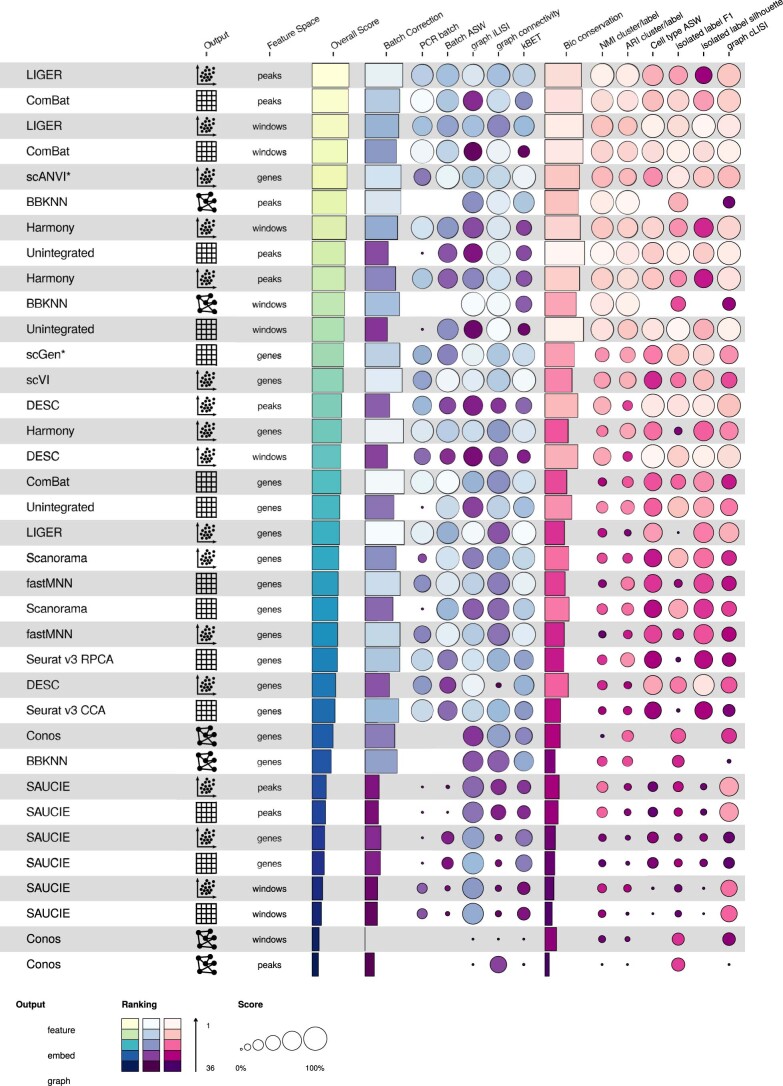
Benchmarking results for all large mouse brain tasks for all feature spaces based on scATAC-seq. Metrics are divided into batch correction (blue, purple) and bio conservation (pink) categories (see Methods for further visualization details). Overall scores are computed by a 40:60 weighted mean of these category scores. Methods that failed to run are omitted.

Overall, most of the methods performed poorly for batch correction across ATAC tasks (Fig. 4, Extended Data Figs. 5 and 6, Supplementary Figs. 19–30 and Supplementary Note 3). Indeed, many methods worsened the data representation: only 27% of integration outputs performed better than the best unintegrated result (on peaks, Fig. 4a and Extended Data Figs. 5 and 6) compared to 85% on RNA tasks. Gene activities were particularly poorly suited to represent scATAC-seq data. Even unintegrated data in gene activity space lacked biological variation in cell identities compared to the same data on peaks or windows. This is also reflected in the poorer bio-conservation scores when comparing unintegrated data between feature spaces. Although features overlap between gene activities and scRNA-seq data, of the methods that performed well on RNA data only scANVI, scVI and scGen consistently performed well on this feature space (Fig. 4b). Indeed, the mean bio-conservation score for integration outputs on gene activity space is substantially lower than on peaks and windows (genes 0.39; peaks 0.61; windows 0.59); although removal of biological variance leads to stronger batch removal (mean batch removal score on genes 0.66; peaks 0.50; windows 0.47).

Focusing on peaks and windows, which represent more informative feature spaces for scATAC-seq data, LIGER performed consistently well (Fig. 4b and Supplementary Figs. 19–22). Although ComBat was ranked among the top methods overall (Fig. 4b), the method underperformed in the small ATAC tasks (Supplementary Figs. 19 and 21) and partially failed to resolve nested batch effects in the large integration task (Supplementary Figs. 26 and 28). Several other methods, such as Seurat v3 RPCA and BBKNN (Fig. 4b), also performed well (and scored better on bio-conservation), especially in the small ATAC tasks (Supplementary Figs. 19 and 21). Yet, these often left batch structure within cell-type clusters and thus failed to fully integrate batches (Supplementary Figs. 25–28 and Supplementary Note 3). In contrast, LIGER and Harmony, which focus on batch removal over bio-conservation (Fig. 4c), fully merged batches within cell-type clusters. This trend could also be seen on the large ATAC peak and window tasks, which proved prohibitively large for most methods due to poor scaling with the number of features (Extended Data Fig. 8).

While LIGER and Harmony’s focus on batch removal indicates that scATAC-seq data integration requires a stronger focus on the removal of batch effects, these two methods balance batch effect removal and bio-conservation differently. LIGER performs stronger batch removal than Harmony, although it leaves some batch structure within cerebellar granule cells on large ATAC tasks. In contrast, Harmony comparatively focuses more on the conservation of biological variation, but still partially overlaps smaller neuronal subtype clusters (Fig. 4c). LIGER, however, also created an artificial biological substructure in the integrated data from a single batch when this was not apparent in unintegrated batches (small peak and window tasks, Supplementary Figs. 25 and 27).

### Scalability and usability

**Extended Data Fig. 7:**
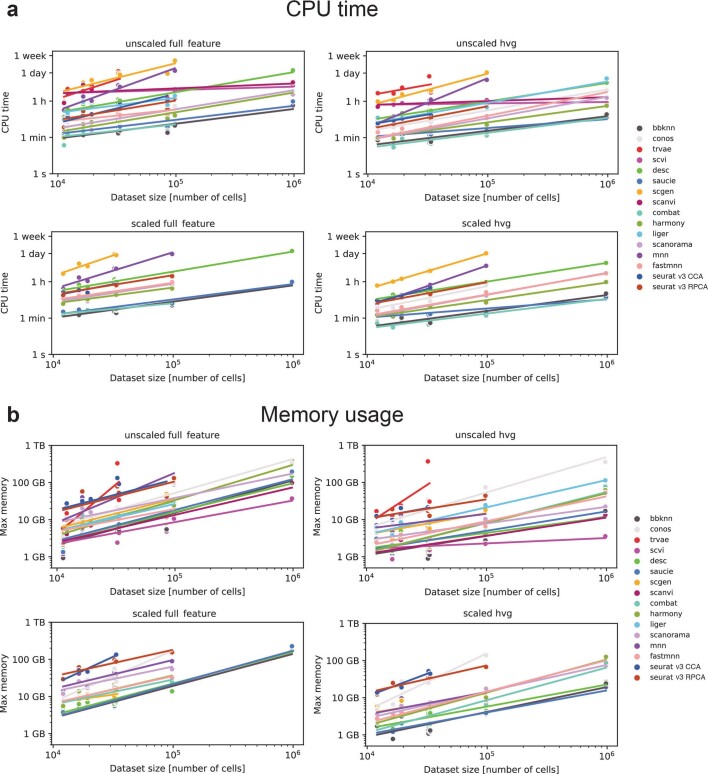
Scalability of each data integration method, separated by preprocessing procedure. (**a**) CPU time for each method (colored dots) and data integration task. (**b**) Maximum memory usage for each method and scenario. Colored lines denote linear fit of log-scaled time or memory vs log-scaled dataset size for each data integration method and pre-processing combination. ATAC task results were included as unscaled full feature runs, and integration runs on peaks and windows feature spaces were excluded.

Monitoring the CPU time and peak memory use reported by our Snakemake pipeline (Extended Data Fig. 7 and Methods), we found that ComBat, BBKNN and SAUCIE performed best in terms of runtime and scVI, scANVI and BBKNN are the most memory efficient. The runtime of scVI and scANVI did not increase with the dataset size due to a heuristic that was suggested to scale training epochs with the number of data points. Given runtime and memory limitations imposed in our benchmark, trVAE could not integrate datasets with >34,000 cells, while Seurat v3, MNN and scGen failed to integrate datasets with >100,000 cells (Supplementary Data 3). As trVAE and scGEN are optimized for GPU infrastructure, their computational burden could be alleviated by using a different computational setup. Furthermore, MNN scaled least favorably in CPU time, while scGEN and trVAE used most CPU time on the tasks we tested.

As expected, using more features led to both longer runtimes and higher memory usage. In contrast, data scaling had little influence on CPU time, but reduced data sparsity when scaling did increase peak memory use.

**Extended Data Fig. 8:**
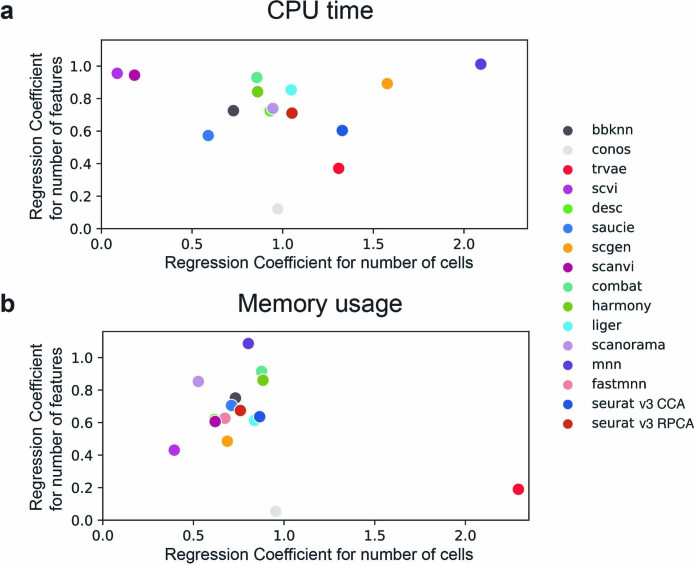
Scalability of each data integration method in terms of number of cells and features. (**a**) Regression coefficients for number of cells and features on CPU time for each method. (**b**) Regression coefficients for number of cells and features on maximum memory usage for each method. Each dot denotes the regression coefficient of the linear fit of log-scaled time or memory vs log-scaled number of cells + log-scaled number of features for each data integration method. All unscaled RNA and ATAC data were modeled to determine the coefficients.

Poor method scalability particularly affected scATAC-seq integration, which typically has a larger feature space. In particular, MNN scales poorly to larger feature spaces both in CPU time and memory usage, while Conos is the least affected (Extended Data Fig. 8). Overall, only seven out of 16 methods could be run on the large ATAC integration tasks for peaks and windows (with >94,000 features). This poor scalability directly hampers the usability of integration methods for this modality.

**Extended Data Fig. 9:**
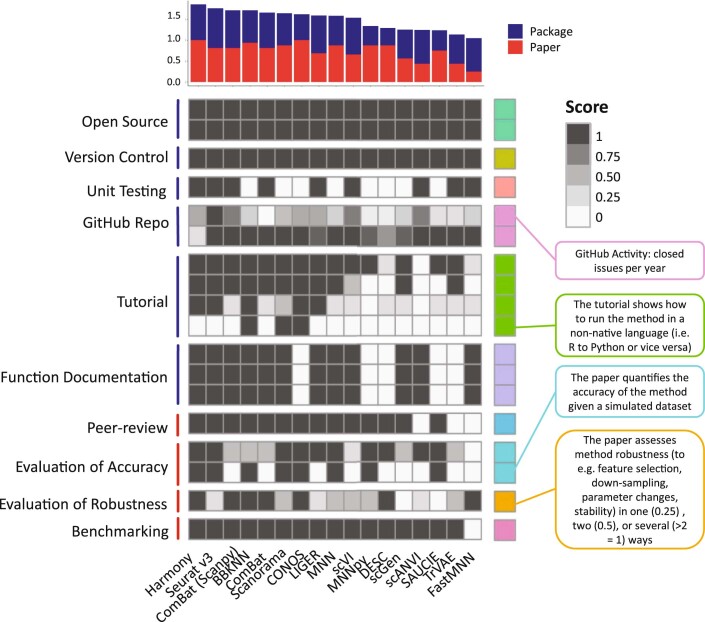
Usability assessment of data integration methods. The usability of each data integration method was assessed via ten categories (labels on the left, see Methods) that consider criteria related to the implementation of the methods (package; dark blue) and information included in the original publications (paper; red). Each score is plotted as a heatmap, and methods are ordered by overall usability score. This score is computed as the sum of the partial average package and paper usability scores, and plotted on top in a barplot. On the right-hand side, criteria with poor scores across methods are highlighted for each category. For the Package scores of ComBat and MNN, we separately considered the original R implementation and the Python implementation that was used in this benchmark. Usability was assessed on December 17th, 2020.

We assessed the usability of methods building on criteria previously applied to evaluate trajectory inference methods^31^ (Methods and Extended Data Fig. 9). Most of the methods are easy to use given the availability of tutorials, function documentation and open source code. However, the activity of the GitHub repository and published evidence on the robustness of the method and its accuracy on real and simulated data are distinguishing factors. Overall, Harmony, Seurat v3 and BBKNN have the best usability for new users. In contrast, DESC, scANVI and trVAE are lacking in usability at the time of writing as they lack function documentation or high-quality tutorials.

## Discussion

We benchmarked 16 integration methods with four preprocessing combinations on 13 integration tasks via 14 metrics that measure tradeoffs between batch integration and conservation of biological variance. Overall, we observed that method performance is dependent on the complexity of the integration task for RNA and simulation scenarios. For example, the use of Harmony is appropriate for simple integration tasks with distinct batch and biological structure; however, this method typically ranks outside the top three when used for complex real data scenarios, which is in agreement with recent benchmarks on simpler batch structures^9,28^. In contrast, on more complex integration tasks, Scanorama (embeddings) and scVI worked well. Methods that used cell annotations to integrate batches (scGen and scANVI) performed well across tasks.

Our overall rankings were based on metrics measuring different aspects of integration success (for an overview, see the website and Supplementary Figs. 31–39). For example, while certain bio-conservation metrics prioritized clearly separated cell clusters, others measured continuous cellular variation such as trajectories and the cell-cycle, or evaluated gene-level output. This diversity of metrics further ensured that, even for integrated graph outputs, it was possible to measure three batch removal and three bio-conservation metrics (Supplementary Table 2). Thus, no individual method ranked highly only by optimizing a single metric, for example, BBKNN, for which the underlying optimization function is similar to the graph iLISI metric. Our metric aggregation approach follows best practices for robust ranking in machine learning tasks^32^ and indeed produced consistent overall rankings when compared to alternatives^31^ (overall rank correlation, Spearman’s *R* > 0.96 for all tasks).

As expected, we observed a consistent tradeoff between bio-conservation and batch effect removal. While BBKNN and Seurat v3 tended to remove batch variation, scANVI and scGen prioritized bio-conservation. Learning a more regularized implicit latent space of each batch mediated stronger batch removal while also removing biological variation. For example, Seurat v3 CCA removed variation within cells from a single batch that otherwise showed substructure in unintegrated data (lung task in Supplementary Note 3).

Scaling the input data typically shifted results toward better batch removal but worse bio-conservation, while HVG selection improved overall performance. Notably, only metrics that measured particular functions or pathways (for example, cell cycle) performed better with full gene sets. This suggests that biological functions are better captured in integrated data if the relevant gene sets are included in the integration.

scATAC-seq batch effects were only consistently overcome by LIGER and Harmony, which prioritize batch removal over conservation of biological variation. Notably, these methods performed particularly well on the peak and window feature space, which conserves cell-type structure better than gene activity features. In general, the choice of feature space strongly determines the tradeoff between batch removal and bio-conservation on scATAC-seq data: peak and window feature spaces conserve biological variation, while gene activity reduces variation between cells but also between batches. Thus, scANVI, which strongly focuses on bio-conservation, is the top-performing method on gene activities. While alternative choices of gene activity scoring may improve this feature space^33,34^, our results indicate that peaks or windows are better suited to integrate and analyze scATAC-seq data. In general, methods that perform well on RNA tasks tend to perform poorly on scATAC-seq data as these often focus on bio-conservation. This is particularly noticeable for the methods that use PCA or singular value decomposition dimensionality reduction (FastMNN, Scanorama, Conos and SAUCIE), indicating that covariance may not sufficiently capture the nonlinear variation in this data modality. Instead, dimensionality reduction approaches designed for scATAC-seq data^35^ in combination with an MNN approach as implemented in FastMNN or Scanorama may represent a promising avenue for future integration approaches for this modality.

In general, deep learning methods showed variable performance: while scANVI, scGen and scVI were top performers, trVAE, DESC and SAUCIE performed poorly. Notably, while scGen and scANVI benefited from cell identity labels to perform well across tasks, scVI and trVAE performed better with increasing cell numbers and batch complexity. scVI performed particularly well when the task contained complex batch effects (for example, microwell-seq, single-cell and single nuclei, or scATAC-seq data) and sufficient numbers of cells were present to fit these effects. With more tunable parameters, deep learning methods are more complex than other benchmarked methods and are more likely to require larger input data and separate hyperparameter optimization for optimal performance; however, this also gives them the flexibility to fit complex batch effects. For scVI, scANVI and scGen, a parameter set optimized for general data integration was used (extracted from the respective tutorials). In contrast, SAUCIE and DESC were optimized for simpler tasks such as clustering, and trVAE was optimized for the more general and difficult task of perturbation modeling. Thus, it is unsurprising that DESC only performed well in the simulated tasks (and the small ATAC gene task) with a clear, simple cluster structure. These parameterization choices also affect method scalability: while SAUCIE and DESC were quick to run, trVAE could not be run on the larger, complex tasks without GPU hardware. Parameter optimization, while out of scope here, is likely to improve the performance of any integration method (for example, see DESC parameter optimization in Supplementary Fig. 40). scVI and scANVI also performed well integrating data from full-length protocols as well as with binary scATAC-seq data, although these data violate the noise model assumption of the method. As the availability of data and accessibility of GPU hardware increases, we expect the performance of neural network methods to overtake that of their counterparts, as has occurred in the field of imaging^36,37^.

**Fig. 5:**
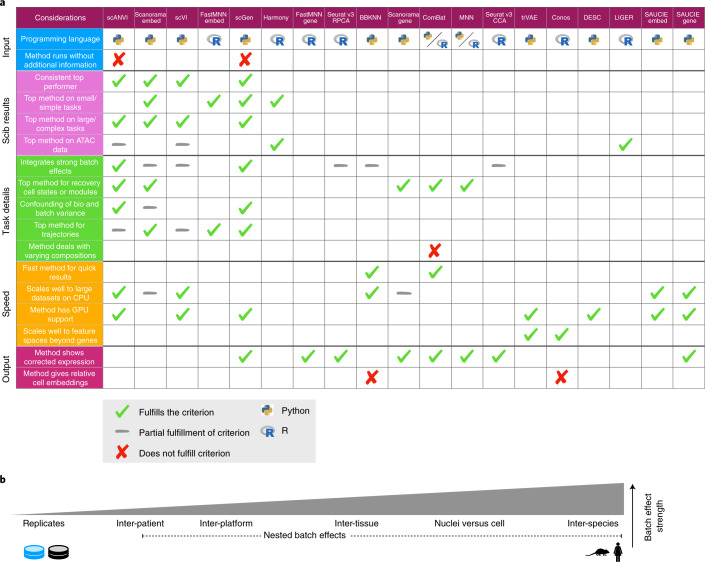
Guidelines to choose an integration method. **a**, Table of criteria to consider when choosing an integration method, and which methods fulfill each criterion. Ticks show which methods fulfill each criterion and gray dashes indicate partial fulfillment. When more than half of the methods fulfill a criterion, we instead highlight the methods that do not by a cross; hence blank spaces denote a cross except in the three rows with labeled crosses. Method outputs are ordered by their overall rank on RNA tasks. Python and R symbols indicate the primary language in which the method is programmed and used. Considerations are divided into the five broad categories (input, scIB results, task details, speed and output), which cover usability (input, output), scalability (speed) and expected performance (scIB results, task details). If not otherwise specified, criteria relate to RNA results. As a dataset specific alternative, method selection can be guided by running the scIB pipeline to test all methods on a user-provided dataset. **b**, Schematic of the relative strength of batch effect contributors in our study.

As a general conclusion, we would advise to choose an integration method according to three criteria: usability, scalability and expected performance (Fig. 5a). While all methods were found usable, their output type can limit the potential downstream applications of integrated data. For example, integrated graphs provide neither relative distances between cells nor corrected gene-expression values that may be required for scoring functional gene programs or performing trajectory inference.

Considering scalability, one might want to rapidly test how integration affects a dataset and thus opt for BBKNN. In contrast, larger datasets may require methods to scale well with the number of cells or features (particularly for scATAC-seq tasks), and availability of GPU infrastructure may direct method choice toward deep learning approaches.

The expected performance of an integration method can derive from the overall results of this study, and from details of the task for which the integration is needed. If cell identity labels are known, it is always beneficial to integrate scRNA-seq batches via scANVI or scGen (for example, as in the recent heart cell atlas^38^). In the absence of labels, given no further information on the integration task, we recommend the top-performing integration methods Scanorama and scVI, especially for sufficiently large datasets. For (smaller) tasks with distinct biological signal, Harmony may be useful. Accounting for task details, the remaining considerations can be divided into five criteria: (1) the strength of the expected batch effect, (2) the need to discern nuanced cell states or recover gene modules, (3) the degree of confounding between batch and biological signals, (4) the existence of continuous cellular phenotypes (trajectories) and (5) compositional shifts in the data (Fig. 5a). We can qualitatively evaluate the strength of various batch effect contributors by the challenge that our diverse set of integration tasks presented to the benchmarked methods (Fig. 5b). Methods that can remove strong batch effects also tend to remove nuanced biological signals or require cell identity labels obtained via per-batch data processing. Thus, if the aim is to find rare cell types and nuanced biological variation, we recommend Scanorama. However, if a broad overview of the data in the presence of strong batch effects is required, we recommend BBKNN or Seurat v3 for smaller datasets. Given sufficient numbers of cells, scVI has shown that it is able to remove strong batch effects while only sacrificing minimal biological variation. Alternatively, scANVI and scGen succeed in integrating across strong batch effects while retaining most nuanced biological variation if this is encoded into the cell identity labels these methods use.

In the presence of particularly strong batch effects, it is worth considering whether removing such an effect is desirable. In the present study, we have defined what we consider batch effect and biological variation per task, yet the distinction between the two is not always straightforward. Effects such as spatial location, species or tissue can be regarded as batch or biology. Moreover, retaining batch effects in a dataset to preserve all nuanced biological variation may be preferable. Here, statistical models can be used to directly analyze raw data relying on harmonized cell annotations while also accounting for batch effects. This type of modeling may also be appropriate across large, aggregated datasets^39^, for which sufficiently powerful data integration methods do not yet exist.

Our benchmarking study will help analysts to navigate the space of available integration methods, and guide developers toward building more efficient methods. Based on the trends we have reported, users can select suitable preprocessing and integration methods for exploratory, integrated data analysis. To enable in-depth characterization of method performance on specific tasks, we have provided the reproducible scIB-pipeline Snakemake pipeline and the scIB python module for users to easily benchmark their particular integration scenario. In addition, we expect that this work will become a reference for method developers, who can build on the presented scenarios and metrics to assess the performance of their newly developed methods on atlas-level data integration tasks.

## Methods

### Datasets and preprocessing

We benchmarked data integration methods on 13 integration tasks: 11 real data tasks and two simulation tasks. For the real data tasks, we downloaded 23 published datasets (see Supplementary Data 2 for a per-batch overview of datasets). All scRNA-seq datasets were quality controlled and normalized in the same way according to published best practices^7^. Specifically, we used scran pooling normalization^40^ (v.1.10.2 unless otherwise specified) and log_+1_ transformation on count data. For data solely available in transcripts per million or reads per kilobase of transcript, per million mapped reads units, we performed log_+1_ transformation without any further normalization. As the datasets typically contained different cell identity annotations we mapped these annotations by matching annotation names, overlaps of data-driven marker gene sets and manual clustering and annotation of cell identities per batch.

For the simulation tasks, data were simulated using the Splatter package^41^ to evaluate data integration methods in a controlled setting. All of our data processing scripts are publicly available as Jupyter notebooks and R scripts at github.com/theislab/scib-reproducibility. For further details on datasets, please see the Supplementary Information.

### Integration methods

We ran the 16 selected data integration methods according to default parameterizations obtained from available tutorials, paper methods or by directly contacting method authors. For further details on how each method was run, please see the Supplementary Information.

### Metrics

We grouped the metrics into two broad categories: (1) removal of batch effects and (2) conservation of biological variance. The latter category is further divided into conservation of variance from cell identity labels, and conservation of variance beyond cell identity labels. Scores from the first category include principal component regression (batch), ASW (batch), graph connectivity, graph iLISI and kBET. In the second category, label conservation metrics include NMI, ARI, ASW (cell-type), graph cLISI, isolated label F1 and isolated label silhouette; label-free conservation metrics include cell-cycle (CC) conservation, HVG conservation and trajectory conservation.

The metrics were run on different output types (Supplementary Table 2). For example, metrics that run on *k*NN graphs can be run on all output types after preprocessing. Similarly, metrics that run on joint embeddings can also be run on corrected feature outputs. Preprocessing was performed in Scanpy (v.1.4.5 commit d69832a). *k*NN graphs were computed using the neighbors function where *k* = 15 unless otherwise specified. Where a joint embedding was available, this graph was computed using Euclidean distances on this embedding, whereas distances were computed on the top 50 principal components where a corrected feature matrix was output.

#### NMI

NMI compares the overlap of two clusterings. We used NMI to compare the cell-type labels with Louvain clusters computed on the integrated dataset. The overlap was scaled using the mean of the entropy terms for cell-type and cluster labels. Thus, NMI scores of 0 or 1 correspond to uncorrelated clustering or a perfect match, respectively. We performed optimized Louvain clustering for this metric to obtain the best match between clusters and labels. Louvain clustering was performed at a resolution range of 0.1 to 2 in steps of 0.1, and the clustering output with the highest NMI with the label set was used. We used the scikit-learn^27^ (v.0.22.1) implementation of NMI.

#### ARI

The Rand index compares the overlap of two clusterings; it considers both correct clustering overlaps while also counting correct disagreements between two clusterings^42^. Similar to NMI, we compared the cell-type labels with the NMI-optimized Louvain clustering computed on the integrated dataset. The adjustment of the Rand index corrects for randomly correct labels. An ARI of 0 or 1 corresponds to random labeling or a perfect match, respectively. We also used the scikit-learn^27^ (v.0.22.1) implementation of the ARI.

#### ASW

The silhouette width measures the relationship between the within-cluster distances of a cell and the between-cluster distances of that cell to the closest cluster^43^. Averaging over all silhouette widths of a set of cells yields the ASW, which ranges between −1 and 1. The ASW is commonly used to determine the separation of clusters where 1 represents dense and well-separated clusters, while 0 or −1 corresponds to overlapping clusters (caused by equal between- and within-cluster variability) or strong misclassification (caused by stronger within-cluster than between-cluster variability), respectively.

To evaluate data integration outputs, we used (1) the classical definition of ASW to determine the silhouette of the cell labels (cell-type ASW) and (2) a modified approach to measure batch mixing. Both metrics were computed on the embeddings provided by integration methods or the PCA of expression matrices in case of feature output. For the bio-conservation score (1), the ASW was computed on cell identity labels and scaled to a value between 0 and 1 using the equation:

$${\mathrm{cell}}\,{\mathrm{type}}\,{\mathrm{ASW}} = ({\mathrm{ASW}}_{\mathrm{C}} + 1)/2,$$

where *C* denotes the set of all cell identity labels.

For the batch mixing score (2), we consider the absolute silhouette width, *s*(*i*), on batch labels per cell *i*. Here, 0 indicates that batches are well mixed, and any deviation from 0 indicates a batch effect:

$$s_{{\mathrm{batch}}}(i) = |s(i)|.$$

To ensure higher scores indicate better batch mixing, these scores are scaled by subtracting them from 1. As we expect batches to integrate within cell identity clusters, we compute the batchASW_*j*_ (ref. ^11^) score for each cell label *j* separately, using the equation:

$${\mathrm{batch}}\,{\rm{ASW}}_j = \frac{1}{{|C_j|}} {\sum \nolimits_{i \in C_j}} {1 - s_{{\mathrm{batch}}}(i)} ,$$

where *C*_*j*_ is the set of cells with the cell label *j* and |*C*_*j*_| denotes the number of cells in that set.

To obtain the final batchASW score, the label-specific batchASW_*j*_ scores are averaged:

$${\mathrm{batch}}\,{\rm{ASW}} = \frac{1}{{|M|}} {\sum \nolimits_{j \in M}} {{\mathrm{batch}}\,{\rm{ASW}}_j} .$$

Here, *M* is the set of unique cell labels.

Overall, a batchASW of 1 represents ideal batch mixing and a value of 0 indicates strongly separated batches. We used the scikit-learn^27^ (v.0.22.1) implementation to compute these scores.

#### Principal component regression

Principal component regression, derived from PCA, has previously been used to quantify batch removal^11^. Briefly, the *R*^2^ was calculated from a linear regression of the covariate of interest (for example, the batch variable *B*) onto each principal component. The variance contribution of the batch effect per principal component was then calculated as the product of the variance explained by the *i*th principal component (PC) and the corresponding *R*^2^(PC_*i*_|*B*). The sum across all variance contributions by the batch effects in all principal components gives the total variance explained by the batch variable as follows:

$${\mathrm{Var}}\left( {C|B} \right) = \mathop {\sum }\limits_{i = 1}^G {\mathrm{Var}}\left( {C|{\mathrm{PC}}_i} \right) \times R^2\left( {{\mathrm{PC}}_i|B} \right),$$

where Var(*C*|PC_*i*_) is the variance of the data matrix *C* explained by the *i*th principal component.

#### Graph connectivity

The graph connectivity metric assesses whether the *k*NN graph representation, *G*, of the integrated data directly connects all cells with the same cell identity label. For each cell identity label *c*, we created the subset *k*NN graph *G*(*N*_*c*_;*E*_*c*_) to contain only cells from a given label. Using these subset *k*NN graphs, we computed the graph connectivity (GC) score using the equation:

$${\mathrm{GC}} = \frac{1}{{|C|}}\mathop {\sum}\nolimits_{c \in C} {\frac{{\left| {{\mathrm{LCC}}\left( {G\left( {N_c;E_c} \right)} \right)} \right|}}{{\left| {N_c} \right|}}} .$$

Here, *C* represents the set of cell identity labels, *|*LCC()*|* is the number of nodes in the largest connected component of the graph and *|N*_*c*_*|* is the number of nodes with cell identity *c*. The resultant score has a range of (0;1], where 1 indicates that all cells with the same cell identity are connected in the integrated *k*NN graph and the lowest possible score indicates a graph where no cell is connected. As this score is computed on the *k*NN graph, it can be used to evaluate all integration outputs.

#### kBET

The kBET algorithm (v.0.99.6, release 4c9dafa) determines whether the label composition of a *k* nearest neighborhood of a cell is similar to the expected (global) label composition^11^. The test is repeated for a random subset of cells, and the results are summarized as a rejection rate over all tested neighborhoods. Thus, kBET works on a *k*NN graph.

We computed *k*NN graphs where *k* = 50 for joint embeddings and corrected feature outputs via the Scanpy preprocessing steps (previously described). To test for technical effects and to account for cell-type frequency shifts across datasets, we applied kBET separately on the batch variable for each cell identity label. Using the kBET defaults, a *k* equal to the median of the number of cells per batch within each label was used for this computation. Additionally, we set the minimum and maximum thresholds of *k* to 10 and 100, respectively. As *k*NN graphs that have been subset by cell identity labels may no longer be connected, we computed kBET per connected component. If >25% of cells were assigned to connected components too small for kBET computation (smaller than *k* × 3), we assigned a kBET score of 1 to denote poor batch removal. Subsequently, kBET scores for each label were averaged and subtracted from 1 to give a final kBET score.

We noted that *k*-nearest-neighborhood sizes can differ between graph-based integration methods (for example, Conos and BBKNN) and methods in which the *k*NN graph is computed on an integrated embedding. This difference can affect the test outcome because of differences in statistical power across neighborhoods. Thus, we implemented a diffusion-based correction to obtain the same number of nearest neighbors for each cell irrespective of integration output type (Supplementary Note 1). This extension of kBET allowed us to compare integration results on *k*NN graphs irrespective of integration output format.

#### Graph LISI

The LISI, a diversity score, was proposed to assess both batch mixing (iLISI) and cell-type separation (cLISI)^21^. LISI scores are computed from neighborhood lists per node from integrated *k*NN graphs. Specifically, the inverse Simpson’s index is used to determine the number of cells that can be drawn from a neighbor list before one batch is observed twice. Thus, LISI scores range from 1 to *N*, where *N* is the total number of batches in the dataset.

Typically, neighborhood lists to compute LISI scores are extracted from weighted *k*NN graphs with *k* = 90 nearest neighbors at a fixed perplexity of $p = \frac{1}{3}k$. These nearest neighbor graphs are constructed using Euclidean distances on PCA or other embeddings. In contrast, integrated graphs that are output by methods such as Conos or BBKNN typically contain far fewer than *k* = 90 neighbors. Running LISI metrics with differing numbers of nearest neighbors per node results in differing sensitivities per neighborhood and thus skews any comparison with graph-based integration outputs. Thus, the original LISI score is not applicable to graph-based outputs.

To extend LISI graph-based integration outputs, we developed graph LISI, which uses the integrated graph structure as an embedded space for distance calculation. The calculated graph distances are then used to determine a consistent number of nearest neighbors per node. We used the shortest path lengths computed via a custom scalable reimplementation of Dijkstra’s algorithm^44^ as a graph-based distance metric (see Supplementary Note 2 for details). Our graph LISI extension produces consistent metric values with the standard LISI implementation for non-graph-based integration outputs (Supplementary Fig. 41). Additionally, we sped up graph LISI scoring via a fast, parallel C^++^ implementation that scales to millions of cells.

As LISI scores range from 1 to *B* (where *B* denotes the number of batches), indicating perfect separation and perfect mixing, respectively, we rescaled them to the range 0 to 1. For iLISI and cLISI this involved a two-step process. First, we computed the median across neighborhoods per method: ${\mathrm{cLISI}} = {\mathrm{median}}\,f(x),x \in X$; ${\mathrm{iLISI}} = {\mathrm{median}}\,g(x),x \in X$. Second, we rescaled the LISI scores as follows: ${\mathrm{cLISI}}:f(x) = \frac{{B - x}}{{B - 1}}$, where a 0 value corresponds to low cell-type separation and ${\mathrm{iLISI}}:g(x) = \frac{{x - 1}}{{B - 1}}$, where a 0 value corresponds to low batch integration.

#### Isolated label scores

We developed two isolated label scores to evaluate how well the data integration methods dealt with cell identity labels shared by few batches. Specifically, we identified isolated cell labels as the labels present in the least number of batches in the integration task. The score evaluates how well these isolated labels separate from other cell identities.

We implemented the isolated label metric in two versions: (1) the best clustering of the isolated label (F1 score) and (2) the global ASW of the isolated label. For the cluster-based score, we first optimize the cluster assignment of the isolated label using the F1 score across louvain clustering resolutions ranging from 0.1 to 2 in resolution steps of 0.1. The optimal F1 score for the isolated label is then used as the metric score. The F1 score is a weighted mean of precision and recall given by the equation:

$$F_1 = 2 \times \frac{{\mathrm{precision} \times {\mathrm{recall}}}}{{{\mathrm{precision}} + {\mathrm{recall}}}}.$$

It returns a value between 0 and 1, where 1 shows that all of the isolated label cells and no others are captured in the cluster. For the isolated label ASW score, we compute the ASW of isolated versus nonisolated labels on the PCA embedding (ASW metric above) and scale this score to be between 0 and 1. The final score for each metric version consists of the mean isolated score of all isolated labels.

#### HVG conservation

The HVG conservation score is a proxy for the preservation of the biological signal. If the data integration method returned a corrected data matrix, we computed the number of HVGs before and after correction for each batch via Scanpy’s highly_variable_genes function (using the ‘cell ranger’ flavor). If available, we computed 500 HVGs per batch. If fewer than 500 genes were present in the integrated object for a batch, the number of HVGs was set to half the total genes in that batch. The overlap coefficient is as follows:

$${\mathrm{overlap}}\left( {X,Y} \right) = \frac{{\left| {X \cap Y} \right|}}{{{\mathrm{min}}\left( {\left| X \right|,\left| Y \right|} \right)}},$$

where *X* and *Y* denote the fraction of preserved informative genes. The overall HVG score is the mean of the per-batch HVG overlap coefficients.

#### Cell-cycle conservation

The cell-cycle conservation score evaluates how well the cell-cycle effect can be captured before and after integration. We computed cell-cycle scores using Scanpy’s score_cell_cycle function with a reference gene set from Tirosh et al.^45^ for the respective cell-cycle phases. We used the same set of cell-cycle genes for mouse and human data (using capitalization to convert between the gene symbols). We then computed the variance contribution of the resulting *S* and *G*2/*M* phase scores using principal component regression (Principal component regression), which was performed for each batch separately. The differences in variance before, Var_before_, and after, Var_after_, integration were aggregated into a final score between 0 and 1, using the equation:

$${\mathrm{CC}}\,{\mathrm{conservation}} = 1 - \frac{{\left| {{\mathrm{Var}}_{{\mathrm{after}}} - {\mathrm{Var}}_{{\mathrm{before}}}} \right|}}{{{\mathrm{Var}}_{{\mathrm{before}}}}}{{{\mathrm{.}}}}$$

In this equation, values close to 0 indicate lower conservation and 1 indicates complete conservation of the variance explained by cell cycle. In other words, the variance remains unchanged within each batch for complete conservation, while any deviation from the preintegration variance contribution reduces the score.

#### Trajectory conservation

The trajectory conservation score is a proxy for the conservation of the biological signal. We compared trajectories computed after integration for certain clusters that had been manually selected during the data preprocessing step. Trajectories were computed using diffusion pseudotime implemented in Scanpy (sc.tl.dpt). We assumed that trajectories found in the unintegrated data for each batch gave the most accurate biological signal. Therefore, the starting cell of the trajectory, after integration, was defined by selecting the most extremal cell from the cell-type cluster that contained the starting cells of the pre-integration diffusion pseudotime, which was based on the first three diffusion components (see the immune cell task description for more details). Only cells from the largest connected component of the neighborhood graph were considered.

We computed Spearman’s rank correlation coefficient, *s*, between the pseudotime values before and after integration (using the function pd.series.corr() in the Pandas^46^ package; v.1.1.1). The final score was scaled to a value between 0 and 1 using the equation

$${\rm{trajectory}}\, {\rm{conservation}} = {({{s}} + 1)}/{2}.$$

Values of 1 or 0 correspond to the same order of cells on the trajectory before and after integration or the reverse order, respectively. In cases where the trajectory could not be computed, which occurs when *k*NN graphs of the integrated data contain many connected components, we set the value of the metric to 0.

#### Ranking and metric aggregation

Metrics were run on the integrated and unintegrated AnnData^47^ objects. We selected the metrics for evaluating performance based on the type of output data (Supplementary Table 2). For example, metrics based on corrected embeddings (Silhouette scores, principal component regression and cell-cycle conservation) were not run where only a corrected graph was output.

The overall score, *S*_overall*,i*_, for each integration run *i* was calculated by taking the weighted mean of the batch removal score, *S*_batch,*i*_, and the bio-conservation score, *S*_batch,*i*_, following the equation:

$$S_{{\mathrm{overall}},i} = 0.6 \times S_{{\mathrm{bio}},i} + 0.4 \times S_{{\mathrm{batch}},i}.$$

In turn, these partial scores were computed by averaging all metrics that contribute to each score via:

$$\begin{array}{l}S_{{\mathrm{bio}},i} = \frac{1}{{\left| {M_{{\mathrm{bio}}}} \right|}}\mathop {\sum}\nolimits_{m_j \in M_{{\mathrm{bio}}}} {f\left( {m_j\left( {X_i} \right)} \right),\,{{{\mathrm{and}}}}} \\ S_{{\mathrm{batch}},i} = \frac{1}{{\left| {M_{{\mathrm{batch}}}} \right|}}\mathop {\sum}\nolimits_{m_j \in M_{{\mathrm{batch}}}} {f\left( {m_j\left( {X_i} \right)} \right)} .\end{array}$$

Here, *X*_*i*_ denotes the integration output for run *i* and *M*_bio_ and *M*_batch_ denote the set of metrics that contribute to the bio-conservation and batch removal scores, respectively. Specifically, *M*_bio_ contains the NMI cluster/label, ARI cluster/label, cell-type ASW, isolated label F1 and silhouette, graph cLISI, cell-cycle conservation, HVG conservation and trajectory conservation metrics, while *M*_batch_ contains the PCR batch, batchASW, graph iLISI, graph connectivity and kBet metrics. To ensure that each metric is equally weighted within a partial score and has the same discriminative power, we min–max scaled the output of every metric within a task using the function *f*(), which is given by:

$$f\left( Y \right) = \frac{{Y - {\mathrm{min}}(Y)}}{{{\mathrm{max}}(Y) - {\mathrm{min}}(Y)}}.$$

Notably, using *z* scores (previously used for trajectory benchmarking^31^) instead of min–max scaling gives similar overall rankings (Spearman’s *R* > 0.96 for all tasks; using scipy.stats.spearmanr from Scipy^48^ v.1.4.1). Our metric aggregation scheme follows best practices for ranking methods in machine learning benchmarks by taking the mean of raw metric scores before ranking^32^. Using this approach, we were able to compute comparable overall performance scores even when different numbers of metrics were computed per run.

Overall method rankings across tasks (for example, Fig. 3b) were generated from the overall scores for each method in each task (without considering simulation tasks). We ranked the methods in each task and computed an average rank across tasks. Methods that could not be run for a particular task were assigned the same rank as unintegrated data on this task. For scRNA-seq tasks, we chose the best performing combination of features (HVG or full features) and scaling flavors for each integration method, and then ranked these from best- to worst-performing to give a final ranking per task. By taking the mean of these per-task rankings we ordered the methods by overall performance across tasks.

### Benchmarking setup

All integration runs were performed using our Snakemake pipeline. Methods were tested with scaled and unscaled data as input, using the full feature (gene/open chromatin window or peak) set or only HVGs. Where HVGs were used, the top 2,000 were selected using a custom method, which selected HVGs in a manner unaffected by batch variance. Specifically, we initially built the hvg_batch function on top of the highly_variable_genes function from Scanpy. Using the standard function from Scanpy, we obtained the top 2000 HVGs per batch with the cell_ranger flavor. The list of HVGs was ranked first by the number of batches in which the genes were highly variable and second by the mean dispersion parameter across batches; the top 2,000 were then selected. This hvg_batch function is freely available as part of the scIB module. Scaled data have zero mean and unit variance per gene; this was performed by calculating *z* scores of the expression data using Scanpy’s sc.pp.scale function applied separately to each batch (scale_batch function in scIB). HVG selection and scaling were not applied in the ATAC tasks, as these are not typical steps in an ATAC workflow.

Data integration runs were performed with 24 cores and 48 threads available to each method (although methods were required to detect available cores without being passed this information); 16 GB of memory per core and 131 GB of shared swap memory were available. Thus, up to 323 GB of memory was available for each run. The runtime limit was set to 4 d (96 h) for RNA runs and 2 d (48 h) for ATAC runs. Some methods ran out of time or memory and were assigned NA values for the respective integration task. The integration methods were run in separate conda environments for R and Python methods to ensure no clashes in dependencies. Details on how to set up these environments can be found on the scIB GitHub repository (www.github.com/theislab/scib). We converted between R and Python data formats using anndata2ri (www.github.com/theislab/anndata2ri) and conversion functions in LIGER and Seurat.

### Usability assessment

We assessed the usability of integration methods, via an adapted objective scoring system. A set of ten categories were defined (adapted from Saelens et al.^31^) to comprehensively evaluate the user-friendliness of each method (Extended Data Fig. 9 and Supplementary Data 6). The first six categories, grouped under a Package score (open source, version control, unit testing, GitHub repository, tutorial and function documentation), assess the quality of the code, its availability, the presence of a tutorial to guide users through one or more examples, GitHub issue activity and responsiveness and (ideally) usage in a nonnative language (that is, from Python to R or vice versa). The other four categories, belonging to a Paper score (peer review, evaluation of accuracy, evaluation of robustness and benchmarking), assess whether the method was published in a peer-reviewed journal, how the paper evaluated the accuracy and robustness of the method, and the inclusion of a comparison with other published algorithms in the paper. Each category evaluated one or multiple related aspects. The mean scores for each category were averaged (mean) to compute two partial scores (Package and Paper), which were summed up to one final usability score. Supplementary Data 6 reports scores and references collected, to the best of our knowledge, for each usability term considered. In particular, we sought information from multiple sources, such as GitHub repositories, Bioconductor vignettes, Readthedocs documentation, original manuscripts and supplementary materials (last updated on 17 December 2020). When multiple package versions were available, we considered only the documentation corresponding to the package version used in this benchmarking. For two integration methods, ComBat and MNN, we computed two separate Package scores corresponding to their original R packages and to the Python implementations that were used in this benchmark.

The GitHub scores were calculated using information downloaded from the GitHub API using the gh R package (last updated on 16 October 2020). This included basic information about the repository itself as well as details about posted issues and comments. From this information we calculated two scores that measure issue activity and issue responsiveness. The activity score was calculated as:

$${\mathrm{activity}} = {\mathrm{log}}_{10}\left(\frac{{{\mathrm{number}}\,{\mathrm{of}}\,{\mathrm{closed}}\,{\mathrm{issues}}}}{{{\mathrm{repository}}\,{\mathrm{age}}\,{\mathrm{in}}\,{\mathrm{years}}}} + 1\right)$$

To get the response score we first calculated a first response time for each issue. We defined the response time as the time until either there was a comment from someone other than the issue author or the issue was closed. If the issue was still open it was ignored. For each repository we then calculated the median time until first response (in days) and the response score was calculated as:

$${\mathrm{response}} = 30 - ({\mathrm{median}}\,{\mathrm{response}}\,{\mathrm{time}})$$

Subtracting from 30 means faster responses get higher scores and makes sure that any repository with a median response faster than a month gets a positive score.

Both scores were rescaled between zero and one to get the final values.

### Scalability assessment

The scalability of all data integration tools was assessed according to CPU time and peak memory use. For each run of the Snakemake pipeline, we used the Snakemake benchmarking function to measure time and peak memory use (max PSS). To score time and memory usage, we used a linear regression model to fit time and memory versus the number of cells on a log-scale separately for each method and each preprocessing combination (completed with curve_fit from scipy.optimize, scipy v.1.3.0). The fit results are shown in Extended Data Fig. 7. Each fit had a slope and an intercept calculated as follows:

$$f(x) = a \times {\mathrm{log}}(x) + b + \varepsilon .$$

These values were used to compute each area under the curve (AUC) where *A* = 10^4^ and *B* = 10^6^, which corresponded to the approximate range of data task sizes in our study. To derive a scalability score from these areas, we scaled all AUCs by the area of the rectangle that covered all curves. Specifically, we chose the width as the difference of the log-scaled bounds and the height *C* as 10^8^ s (≅3 years or ≅24 days on 48 cores) and 10^7^ MB (≅10 TB), respectively:

$${\mathrm{AUC}}_{{\mathrm{scaled}}} = \frac{{0.5 \times ({\mathrm{log}}(B) - {\mathrm{log}}(A)) \times (f(B) + f(A))}}{{({\mathrm{log}}(B) - {\mathrm{log}}(A)) \times {\mathrm{log}}(C)}} = \frac{1}{2} \times \frac{{f(B) + f(A)}}{{{\mathrm{log}}(C)}}.$$

Methods that scale well have a low AUC and, consequently, a low scaled AUC. To obtain a consistent scoring scheme, we inverted the scaled AUCs:

$$s = 1 - {\mathrm{AUC}}_{{\mathrm{scaled}}}.$$

Finally, we reported the scalability scores for CPU time and peak memory use per method and preprocessing combination.

To evaluate how integration methods scale with increasing numbers of features, we fitted further linear regression models with CPU time and memory respectively as the dependent variable and both the number of cells and the number of features on a log-scale as the independent variables, as follows:

$$f(x) = \beta _0 + \beta _1 \times {\mathrm{log}}(N) + \beta _2 \times {\mathrm{log}}(F) + \varepsilon ,$$

where *f*(*x*) denotes the log-scaled CPU time or memory consumption, *N* denotes the number of cells in the task and *F* denotes the number of features. This model was fit separately for each method using the ordinary least squares fit function ‘ols’ from the statsmodels.formula.api module (statsmodels v.0.11.1) on unscaled data (using both full feature and HVG preprocessed data, as well as all ATAC results). We reported the regression coefficients for both number of cells, *β*_1_, and number of features, *β*_2_, to compare scalability between methods (Extended Data Fig. 8).

### Visualization

Inspired by the code of Saelens et al.^31^, we implemented two plotting functions in R. The first visualization displays each integration task separately and shows the complete list of tested integration runs ranked by the overall performance score. Individual and aggregated scores are represented by circles and bars, respectively. The color scheme indicates the overall ranking of each method.

The second visualization provides an overall view of the best performing flavors of each integration method. The overall performance scores for the optimal preprocessing combination for each method for all tasks is shown as a horizontal bar chart. Methods were ranked as detailed in the Ranking and metric aggregation section above and bars were shaded by rank. Moreover, we displayed two partial usability scores related to package and paper, two scalability scores related to time and memory consumption, and the overall scores obtained in the two simulation tasks (although these scores were not used for the ranking). Again, bar lengths represented scores and the color scheme indicated the ranking.

### Results website

Additional results and supplementary figures are available in an interactive format at https://theislab.github.io/scib-reproducibility/. This website was produced using the rmarkdown package (v.2.3)^49^ in R (v.4.0.0). Visualizations were created with ggplot2 (v.3.3.2)^50^ and interactive tables with the reactable package (v.0.2.2). The drake workflow manager (v.7.12.5)^51^ was used to build the website and the environment was managed using renv (v.0.11.0). Source code for the website is available at https://github.com/theislab/scib-reproducibility/tree/main/website.

### Reporting Summary

Further information on research design is available in the Nature Research Reporting Summary linked to this article.

## Online content

Any methods, additional references, Nature Research reporting summaries, source data, extended data, supplementary information, acknowledgements, peer review information; details of author contributions and competing interests; and statements of data and code availability are available at 10.1038/s41592-021-01336-8.

## Supplementary information

Supplementary Information Supplementary Methods, Figs. 1–42, Tables 1 and 2, Notes 1–3 and References.

Reporting Summary

Supplementary Data 1 Metric results for all benchmarking runs.

Supplementary Data 2 Summary statistics of data integration tasks.

Supplementary Data 3 Completion status of all attempted integration method runs with error message summaries.

Supplementary Data 4 Detailed breakdown of datasets used in the immune cell human and human/mouse tasks.

Supplementary Data 5 Markers used to reannotate the immune cell tasks with harmonized labels.

Supplementary Data 6 Usability metric results and explanation for each value.

Supplementary Data 7 Overview of cellular identities in the pancreas and mouse brain integration tasks.

## Data Availability

We reprocessed the following public datasets for our integration tasks: pancreas GSE81076, GSE85241, GSE86469, GSE84133, GSE81608 (Gene Expression Omnibus (GEO)) and E-MTAB-5061 (ArrayExpress); immune cell bone marrow GSE120221 and GSE107727 (GEO); immune cell peripheral blood 10X data from https://support.10xgenomics.com/single-cell-gene-expression/datasets/3.0.0/pbmc_10k_v3, GSE115189, GSE128066 and GSE94820 (GEO); in addition to the Mouse Cell Atlas datasets of bone marrow and peripheral blood downloaded from https://figshare.com/articles/MCA_DGE_Data/5435866. For the lung integration task, the Drop-seq data were available from GEO (GSE130148), while the 10X data were obtained directly from the authors. For mouse brain (RNA), we obtained the raw count matrix for the scRNA-seq dataset from GEO (GSE110823), the annotated count matrix (10X Genomics protocol) from Zeisel et al. (http://mousebrain.org; file name L5_all.loom) and the count matrices per cell type (Drop-seq protocol) from Saunders et al. (http://dropviz.org/; DGE by Region section). Fluorescence-activated cell-sorted mouse brain tissue data (Smart-seq2 protocol, myeloid and nonmyeloid cells, including the annotation file ‘annotations_FACS.csv’) from Tabula Muris were obtained from figshare (https://figshare.com/projects/Tabula_Muris_Transcriptomic_characterization_of_20_organs_and_tissues_from_Mus_musculus_at_single_cell_resolution/27733). For the mouse brain (ATAC) integration, we used FASTQ files from Fang et al. (six samples, single-nucleus ATAC-seq protocol; retrieved from http://data.nemoarchive.org/biccn/grant/cemba/ecker/chromatin/scell/raw/) and Cusanovich et al. (four samples, combinatorial indexing scATAC-seq protocol; GEO accession number GSE111586) and we retrieved fragment and index files from a 10X Genomics dataset for fresh adult mouse brain cortex (sample retrieved from https://support.10xgenomics.com/single-cell-atac/datasets/1.2.0/atac_v1_adult_brain_fresh_5k). Our reprocessed versions of these datasets are publicly available as preprocessed Anndata objects on Figshare (10.6084/m9.figshare.12420968^52^). The output data from all metric runs are available in Supplementary Data 1.

## Extended data

are available for this paper at 10.1038/s41592-021-01336-8.

## Code availability

Notebooks and R scripts used for data preprocessing, visualization and the code for the website is available at https://github.com/theislab/scib-reproducibility. The preprocessing, integration and evaluation metric functions with relevant parameterizations have been made available is our scIB Python package at https://github.com/theislab/scib. Our benchmarking workflow is provided as a reproducible Snakemake pipeline at https://github.com/theislab/scib-pipeline. All integration and evaluation metric outputs can be viewed on our scIB website at https://theislab.github.io/scib-reproducibility/.
