# Supplementary material for: Benchmarking atlas-level data integration in single-cell genomics
Source: Nat Methods. 2021 Dec 23;19(1):41–50. doi: 10.1038/s41592-021-01336-8 (PMC8748196; doi:10.1038/s41592-021-01336-8)
Supplement: Supplementary file 1 — Supplementary Methods, Figs. 1–42, Tables 1 and 2, Notes 1–3 and References. [file 41592_2021_1336_MOESM1_ESM.pdf]

nature portfolio

https://doi.org/10.1038/s41592-021-01336-8

Supplementary information

Benchmarking atlas-level data integration in single-cell genomics

In the format provided by the authors and unedited

Benchmarking atlas-level data integration in single-cell genomics - Supplementary Information

Luecken MD[1], Büttner M[1], Chaichoompu K[1], Danese A[1], Interlandi M[2], Mueller MF[1], Strobl DC[1], Zappia L[1,3], Dugas M[4], Colomé-Tatché M[1,5,6]*, Theis FJ[1,3,5]*

[1] Institute of Computational Biology, Helmholtz Zentrum München, German Research Center for Environmental Health, Neuherberg, Germany

[2] Institute of Medical Informatics, University of Münster, Münster, Germany

[3] Department of Mathematics, Technische Universität München, Garching bei München, Germany

[4] Institute of Medical Informatics, Heidelberg University Hospital, Heidelberg, Germany

[5] TUM School of Life Sciences Weihenstephan, Technical University of Munich, Freising, Germany

[6] Biomedical Center (BMC), Physiological Chemistry, Faculty of Medicine, Ludwig Maximilian University of Munich, Planegg-Martinsried, Germany

*Correspondence: maria.colome@bmc.med.lmu.de; fabian.theis@helmholtz-muenchen.de

Supplementary Information **5**
Datasets and preprocessing 5
Pancreas integration task 5
Immune cell integration tasks (human and mouse) 6
Lung atlas integration task 7
Mouse brain integration task (RNA) 8
Mouse brain integration task (ATAC) 10
Simulations 12
Integration methods 13
ComBat 13
Matching mutual nearest neighbors (MNN) 13
FastMNN 14
Scanorama 14
scVI 15
scANVI 15
Batch-balanced k-nearest neighbors (BBKNN) 16
Clustering on network of samples (Conos) 17
Seurat v3 (CCA and RPCA) 17
Harmony 18
LIGER 19
scGen 19
Transformer Variational Autoencoder (trVAE) 20
SAUCIE 21
DESC 21
Supplementary Figures **23**
Erythrocyte development trajectories on immune (human) 23
Benchmarking metric results 24
Immune (human) 24
Immune (human/mouse) 25
Simulation 1 26
Simulation 2 27
Pancreas 28
Lung 29
Mouse brain 30
Embeddings 31
Immune (human) 31
Immune (human/mouse) 33
Simulation 1 34

Simulation 2 35
Pancreas 36
Lung 37
Mouse brain 38
Trajectories across species 39
Benchmarking metric results for ATAC tasks 41
Mouse brain (ATAC) - windows 41
Mouse brain (ATAC) - peaks 43
Mouse brain (ATAC) - gene activity 45
Embeddings for ATAC tasks 47
Mouse brain (ATAC) - windows 47
Mouse brain (ATAC) - peaks 49
Mouse brain (ATAC) - gene activity 51
Metric rank distributions 53
Immune (human) 53
Immune (human/mouse) 54
Simulation 1 55
Simulation 2 56
Pancreas 57
Lung 58
Mouse brain 59
Mouse brain (ATAC) - all feature spaces 60
Parameter optimization - Pancreas task 62
iLISI comparison 63
Trajectories - addendum 64

Supplementary Table 1: Data integration methods 65

Supplementary Table 2: Metrics runs 70

Supplementary Note 1: Extending kBET for fair assessment of graph-based integration results 71

Supplementary Note 2: Graph LISI extends LISI to graph-based integration results 73

Supplementary Note 3: Detailed analysis of Integration tasks 75

3.1 Immune cells 75
3.2 Simulation 1 78
3.3 Simulation 2 79
3.4 Pancreas 80
3.5 Lung atlas 82
3.6 Mouse brain (RNA) 84

3.7 Mouse brain ATAC tasks 87
3.7.1 Small and large ATAC tasks based on 5k-bp window matrices 88
3.7.2 Small and large ATAC tasks based on peak matrices 89
3.7.3 Small and large ATAC tasks based on gene activity matrices 90

Supplementary References 91

Supplementary Information

Datasets and preprocessing

Pancreas integration task

We used six publicly available human pancreas datasets. Specifically, we used a pre-annotated collection of four datasets from the Satija lab[1–4] (retrieved from https://satijalab.org/seurat/v3.0/integration.html on 28/08/2019) with accession codes GSE81076, GSE85241, GSE86469 (GEO), and E-MTAB-5061 (ArrayExpress). The two additional human pancreas datasets were provided in a pre-annotated format by the Hemberg lab[5,6] (https://hemberg-lab.github.io/scRNA.seq.datasets/human/pancreas/ retrieved on 28/08/2019); their GEO accession codes are GSE84133 and GSE81608. It should be noted that only the SMARTseq2 (E-MTAB-5061)[4] and the inDrop (GSE84133)[6] datasets contained integer count data, whereas the CEL-seq (GSE81076) and CEL-seq2 (GSE85241) datasets were adjusted by the authors to represent count-like data[2,7]. We normalized all datasets that contained count data with scran pooling[8] in a joint normalization run. This excluded the dataset from Xin *et al.*[5] which was provided in normalized units of RPKM. Finally, all datasets were log+1-transformed. In total, there were 16,382 cells in the pancreas integration task with 18,771 genes. Each dataset was treated as a batch, except for the inDrop dataset[6], in which each donor was treated as a batch.

Immune cell integration tasks (human and mouse)

The immune cell task contained immune cells from eight datasets comprising human and mouse cells from bone marrow and peripheral blood. Bone marrow datasets were retrieved from Oetjen et al.[9] (three human donors), Dahlin et al.[10] (four mouse samples), and the Mouse Cell Atlas[11] (MCA; three mouse samples). For peripheral blood data, mouse samples were downloaded from the MCA[11] (six samples) and human samples were obtained from 10X Genomics[12], Freytag *et al.*[13], Sun *et al.*[14] and Villani *et al.*[15]. Details on the retrieval location of datasets, the different protocols used, and ways in which samples were chosen for analysis can be found in **Supplementary Data 4**.

Quality control was performed separately for each sample. Sample-specific thresholds were chosen for the number of genes, the fraction of mitochondrial counts, and the number of UMI counts per cell. Datasets for which count data were available were individually normalized by scran pooling[8]. This excludes the data of Villani *et al.*[15], which included only TPM values. All datasets were log+1-transformed in Scanpy (version 1.4.4 commit bd5f862)[16].

To create a consistent set of cell identity annotations across datasets, we harmonized the existing labels and annotated cells from datasets in which no labels were available. First, the label sets suggested by the MCA and Oetjen *et al.*[9], were harmonized by string matching. In the second step, we collected a number of cell identity markers from the literature (**Supplementary Data 5**) and tested them, first on the pre-annotated samples, and then on the remaining samples. This procedure allowed us to refine the annotation by adding a second layer of cell labels. Where necessary, we performed sub-clustering to improve the annotations. Finally, if the annotations could not be mapped due to coarse labeling, we removed cell populations.

We created two integration tasks from the immune cell data: one containing only human samples, and one containing both human and mouse samples. The human task included cross-tissue integration of immune cells from many donors; the combined task added the complexity of cross-species integration. To integrate human and mouse data into a single data object, we mapped mouse genes (MGI symbol) to their human counterparts (HGNC symbol) using the R package biomaRt (version 2.38.0)[17]. We retained only those genes that were mapped in all batches: 8,135 genes in total. The human integration task contained 33,506 cells (with 12,303 genes), whereas the combined task contained 97,952 cells. Sample IDs were used as batches for data integration.

To test the conservation of trajectories following data integration, we considered the process of erythropoiesis in the human and mouse bone marrow datasets. Specifically, we extracted HSPCs, MPs, EPs, and mature erythrocytes for each batch. We generated a trajectory for each sample using Scanpy's diffusion maps[18] and diffusion pseudotime[19] functions. The root cell for pseudotime analysis was selected from the HSPCs cluster upon evaluation of the diffusion components. Specifically, we selected the cell that was assigned the maximum or minimum value of the first three diffusion components as the root cell.

Lung atlas integration task

Single-cell expression data for the lung integration task was retrieved from the work of Vieira Braga et al.[20], who created a lung atlas that includes samples from three labs that were generated using Drop-seq and 10X Chromium. The Drop-seq data was available from GEO under accession code GSE130148, while the 10X data was obtained directly from the authors in a SoupX-corrected count matrix. We used three healthy datasets from Vieira Braga et al.[20]: the 10X and Drop-seq transplant datasets, along with 10X lung biopsy data. Nasal brush and lung

brush samples were not included in the integration task, as suggested by the original authors, due to the cell identity populations being distinct from the other three datasets. However, we did include lung biopsy data, which comes from a distinct spatial location (the airways) relative to the location of transplant samples (the parenchyma). Following quality control filtering, the data contained 16 donors, with one sample per donor, and 32,472 cells with 15,148 genes.

Data were normalized by scran pooling[8], which was applied to individual datasets. As the 10X datasets and the Drop-seq dataset contained different cell annotations, the annotations were harmonized using fuzzy string matching and overlaps of marker genes determined by a t-test performed in Scanpy[16] (version 1.4.5 commit d69832a). Where annotations could not be mapped due to coarse labeling or where cell populations corresponded to filtered-out datasets, the cell populations were removed (annotations: *Mesothelium*, *Transformed epithelium*, *Ciliated (Nasal)*, *Goblet 1 (Nasal)*, *Goblet 2 (Nasal)*, and *Smooth Muscle Cells*). Donor IDs were used as batches for data integration.

Mouse brain integration task (RNA)

The mouse brain RNA task consisted of four publicly available scRNA-seq and snRNA-seq mouse brain studies[21–24], in which additional information on cerebral regions was provided. We obtained the raw count matrix for the snRNA-seq dataset (SPLiT-seq protocol) of Rosenberg *et al.*[21] (GEO accession ID: GSE110823), the annotated count matrix (10X Genomics protocol) from Zeisel *et al.*[22] (http://mousebrain.org; file name L5_all.loom, downloaded on 09/09/2019), and the count matrices per cell type (Drop-seq protocol) from Saunders *et al.*[24] (http://dropviz.org/; *DGE by Region* section, downloaded on 30/08/2019). FACS-sorted mouse brain tissue data (Smart-seq2 protocol, myeloid and non-myeloid cells, including the annotation file "annotations_FACS.csv") from Tabula Muris[23] were obtained from figshare[25]

(https://figshare.com/projects/Tabula_Muris_Transcriptomic_characterization_of_20_organs_and_tissues_from_Mus_musculus_at_single_cell_resolution/27733; retrieved 14/02/2019).

We harmonized cluster labels via fuzzy string matching, attempting to preserve the original annotation wherever possible. Specifically, we annotated 10 major cell types (neurons, astrocytes, oligodendrocytes, oligodendrocyte precursor cells, endothelial cells, brain pericytes, ependymal cells, olfactory ensheathing cells, macrophages, and microglia).

From Saunders *et al.*[24], we used the additional annotation data table to obtain 585 reported cell types (annotation.BrainCellAtlas_Saunders_version_2018.04.01.txt, retrieved from http://dropviz.org/ on 30/08/2019). Among these cell types, some were annotated as *endothelial tip*, *endothelial stalk* and *mural,* which had no correspondence in other datasets. Thus, we re-annotated these cell types as follows: Louvain clustering (default resolution parameter 1.0) was applied to cluster cells; gene expression profiling was conducted using the *rank_genes_groups* function in Scanpy (t-test); and microglia (*C1qa*), oligodendrocytes (*Plp1*), astrocytes (*Gfap* and *Clu*), and endothelial cells (*Flt1*) were assigned using marker gene expression.

In addition, we harmonized brain region information where possible. In total, we annotated 15 different brain regions (the amygdala, hippocampus, thalamus, hypothalamus, cortex, olfactory bulb, striatum, cerebellum, midbrain, medulla, substantia nigra, entopeduncular nucleus, globus pallidus and nucleus basalis, pons and spinal cord). It must be noted that Rosenberg *et al.*[21] inferred brain regions; thus, 66,648 cells in this dataset were not assigned to a brain region (marked as *Unknown* in the data).

Finally, we applied scran normalization[8] separately to each dataset and log+1-transformed the count matrices. In total, this mouse brain integration task contained 978,734 cells with 14,858 genes. Datasets were treated as batches for data integration.

Mouse brain integration task (ATAC)

The mouse brain ATAC task consists of three scATAC-seq datasets. We used FASTQ files from Fang *et al.*[26,27] (six samples, single nucleus ATAC-seq protocol; retrieved from http://data.nemoarchive.org/biccn/grant/cemba/ecker/chromatin/scell/raw/) and Cusanovich *et al.*[28] (four samples, combinatorial indexing scATAC-seq protocol; GEO accession number GSE111586) and we retrieved fragment and index files from a 10X Genomics dataset for fresh adult mouse brain cortex (sample retrieved from https://support.10xgenomics.com/single-cell-atac/datasets/1.2.0/atac_v1_adult_brain_fresh_5k on 19/08/2020). We aligned the data from Cusanovich et al. and Fang et al. on the GRCm38 reference genome (https://cf.10xgenomics.com/supp/cell-atac/refdata-cellranger-atac-mm10-1.2.0.tar.gz) using BWA[29] (version 0.7.17; for paired-end reads: bwa mem -w 2000). We used SAMtools[30] (version 1.10) to filter low-quality alignments with Q score below 30 and converted the bam files into fragment and index files using bam2bed from BEDTools[31] (version 2.29.0) and tabix from HTSlib[30] (version 1.10.2).

We generated 5000 bp non-overlapping windows using epiScanpy[32] and called peaks for each sample using MACS2[33] (--nomodel --keep-dup all --extsize 200 --shift -100). As the peaks called per dataset differ, we used the union of the three peak files, filtered out peaks that were too short (<300bp), segmented peaks that were too long (>1000bp), and filtered redundant peaks to obtain a common set of unique peaks for all datasets. Finally, we built count matrices and conducted the quality control steps for the different data scenarios using epiScanpy[32] (version 0.2.2). We built gene activity matrices from the filtered peak matrices using epiScanpy. The gene activity matrices were then further filtered to remove low quality cells, the datasets

were concatenated, and the resulting matrix was finally normalized and log-transformed. The gene activity matrices corresponded to the number of open peaks at the gene body and the promoter (5 kb upstream of the TSS) for every gene, using the Ensembl transcripts from gencode vM23 primary annotation as reference.

The integration task was run on two data scenarios (small and large datasets) using windows, peaks and gene activity count matrices. The small integration task consisted of the 10X Genomics dataset, together with one sample from Cusanovich *et al.*[28] (WholeBrainA_62216) and one sample from Fang *et al.*[26] (CEMBA180305_2B), which had been downsampled to 4000 cells each, to balance the numbers of cells among batches. After pre-processing, the numbers of cells and features for the small integration tasks slightly varied between tasks, resulting in 110,724 windows x 10,761 cells, 94,088 peaks x 11,597 cells, and 3,429 genes x 11,270 cells. The large integration task consisted of four samples from Cusanovich *et al.*[28] (WholeBrainA_62816, WholeBrainA_62216, PreFrontalCortex_62216, Cerebellum_62216), six samples from Fang *et al.*[26] (CEMBA180305_2B, CEMBA180306_2B, CEMBA180226_1A, CEMBA180227_1A, CEMBA180308_3B, CEMBA180312_3B), and the 10X Genomics dataset. Thus, the combined data consisted of 84,813 cells in total, and the numbers of features per task was 118,723 windows, 96,924 peaks, and 3,580 genes after pre-processing.

We annotated the cells in seven major cell types using existing annotations and marker genes (astrocytes: Aldh1l1, Gfap, Gja1, S100b, Slc01c1; endothelial cells: Pecam1, Vcam1, Sele, Kdr; excitatory neurons: Slc17a7, Slc17a8, Satb2, Arpp21, Neurod1, Neurod2; inhibitory neurons: Gad1, Gad2, Grik1, Spock3, Sst, Pvalb, Ngnf, Vip; microglia: Cd68, Cd14, Fcgr1, Ms4a7, Fcer1a, Cd163, Ly6c1, Fcna, Lgals2, Chi3l3, Mac1, Itgam, S100a8, S100a9, Tlr2, Tlr4, Cd80, Mertk; oligodendrocytes: Mag, Mog, Man1, Gm21984, Olig1, Olig2). Cerebellar granule cells were

distinguished from other neurons based on tissue annotations and pre-existing cell type annotation.

Simulations

We generated synthetic datasets using an extended version of the *Splat* simulation method available in the Splatter package[34] (version 1.10.0). The standard *Splat* model produces batches with equal cell group proportions and expected library sizes. In order to modify these factors, we first generated a larger dataset in which each batch had an equal number of cells and each group was present in equal proportions. We then used a downsampling procedure to remove cells from each batch until the desired cell group proportions were obtained. The desired difference in the number of counts per cell between batches was achieved using the *downsampleMatrix* function from the DropletUtils package[35,36] (version 1.6.1) to downsample the resulting counts matrix. Basic quality control, involving the removal of cells >2 median absolute deviations below the median of counts per cell or number of expressed genes per cell within each batch, was then performed on the simulated data using the *quickPerCellQC* function in the Scater package[37] (version 1.14.6). Genes expressed in <1% of cells in the whole simulation were also removed. This resulted in an integration task consisting of 12,097 cells and 9,799 genes in six batches.

To create the nested batch effect simulation scenario, we added a step between adjusting cell group proportions and downsampling counts in order to create a sub-batch structure. For each sub-batch, we used the *Splat* model to simulate a second count matrix with the same number of cells as the sub-batch but a lower expected library size and no cell group structure. We then added this noise matrix to the counts for cells in that sub-batch. Quality control for the nested batch scenario was performed at the sub-batch level, and sub-batches were used as batch IDs

for integration. The nested batch integration task consisted of 19,318 cells and 10,000 genes in 16 nested sub-batches (four sets of four sub-batches).

Integration methods

We ran each method according to defaults provided by the authors and contacted them if errors were encountered. These default parameterizations and accompanying method descriptions are detailed below.

ComBat

ComBat[38] is a batch correction method developed for bulk gene expression microarray data. It uses a linear mixed effect model that fits the batch effect's contribution both to the mean expression and the variance in expression. We ran ComBat as it is implemented in Scanpy (version 1.4.5 commit d69832a) via the *combat* function. ComBat returns a corrected gene expression or open chromatin matrix. For the usability assessment, we considered both the original version implemented in R (https://bioconductor.org/packages/sva/) and the Scanpy implementation (**Extended Data Fig. 9**). The two versions thus have the same *Paper* score, but differ in their *Package* scores.

Matching mutual nearest neighbors (MNN)

MNN first detects mutual nearest neighbors in two datasets (or batches) and then infers a projection of the second dataset into the first dataset, which serves as a reference hyperplane[39]. This integrated dataset serves as a new reference to iteratively integrate more datasets. We ran MNN using the *mnn_correct* function from mnnpy (https://github.com/chriscainx/mnnpy version 0.1.9.5). The default parameters were used, including an additional cosine normalization of the

input matrix. MNN returns a corrected gene expression or open chromatin matrix. For the usability assessment, we considered both the original version implemented in R (https://bioconductor.org/packages/batchelor/) and the Python implementation from mmnpy (**Extended Data Fig. 9**). The two versions thus have the same *Paper* score, but differ in their *Package* scores.

FastMNN

FastMNN is a variant of the MNN method that has been modified for improved speed and robustness. Instead of finding nearest neighbours in the original feature space a multi-sample PCA is performed and this embedding is used for MNN integration. We ran the *fastMNN* function from the batchelor Bioconductor package (version 1.4.0, https://bioconductor.org/packages/batchelor/) with default parameters. The primary output of FastMNN is a joint embedding but a corrected expression or open chromatin matrix is also returned. We considered both outputs for benchmarking but note that the method authors suggest that the reconstructed features should not be used for quantitative analysis. There is no associated publication for FastMNN as it is an extension of MNN and thus it scores poorly on the Paper usability metrics, although it performs well for package usability.

Scanorama

The Scanorama algorithm is a further extension to the MNN method based on the concept of panoramic stitching. It finds similar cells across datasets using a k-nearest neighbor search, and then reduces the connections to a set of mutual nearest neighbors[40]. Subsequently, all data points are embedded in a joint hyperplane. In the absence of a clear tutorial, we ran Scanorama

(version 1.4) via the *correct_scanpy* function with the option *return_dimred=True* to obtain a joint embedding as well as a corrected expression or open chromatin matrix.

scVI

The scVI model combines a variational autoencoder (a neural network) with a hierarchical Bayesian model[41]. The negative binomial distribution is used to describe the gene expression of each cell, conditioned on the batch variable and unobserved factors such as differences in sensitivity between measurements. Thus, scVI takes into account fixed and random effects in the data. The output of scVI is a low-dimensional representation in latent space (an embedding). Notably, scVI expects a raw count matrix as input; this was not always available in our integration tasks. We ran scVI (version 0.6.6) using the parameterizations from the *scanpy_pbmc3k* (https://scvi.readthedocs.io/en/stable/tutorials/scanpy.html) and the *harmonization* (https://scvi.readthedocs.io/en/stable/tutorials/harmonization.html) tutorial notebooks. This parameterization includes a model with negative binomial reconstruction loss, a 30-dimensional latent space, 128 nodes in the hidden layer, and n_layers = 2. After consulting with the authors, the model was trained for 400*(20,000/*N*) epochs where *N* is the size of the dataset, while implementing a maximum of 400 epochs for small datasets. scVI returns a joint embedding of cells from all batches.

scANVI

scANVI extends the scVI model by including semi-supervised learning on provided cell type labels. Originally, scANVI was created to transfer existing cell labels to unannotated cells in an scVI-style integration. However, by training the model with all cell type labels, scANVI is able to inform the latent space with these labels and provide an optimized integration. It should be

noted that this is not the intended way of running scANVI (which is with only partial cell type information), but it is potentially the best way to generate an optimal embedding.

We ran scANVI (scVI version 0.6.6) using the parameterizations from harmonisation-CitePure -scANVI (https://github.com/chenlingantelope/HarmonizationSCANVI/blob/master/notebooks/harmonization-CitePure-SCANVI.ipynb). We pretrain the model with scVI as described in the scVI section and subsequently apply scANVI. After consultation with the method authors, we adapted the number of epochs for scANVI training to scale with the number of iterations for the initialisation with scVI. These adaptations were consistent across all runs of scANVI and were not chosen or optimized based on any data that was used for benchmarking. scANVI returns a joint embedding of cells from all batches.

Batch-balanced k-nearest neighbors (BBKNN)

BBKNN[42] first computes a k-nearest neighbor graph within each batch. It then computes the k-nearest neighbors of all cells to all other batches. The resulting graph contains a number of irrelevant connections across cell types; therefore, BBKNN computes a connectivity score for each pair of cells similar to the UMAP algorithm[43]. The symmetrized connectivity score represents the connection of each pair of cells. Thus, BBKNN ultimately returns a weighted neighborhood graph. Notably, BBKNN requires at least some cell types to be shared across batches. We ran BBKNN (version 1.3.5) using the *bbknn* function with mainly default parametrization of k = 3 neighbors within each batch. We adapted the default parameterization to large data scenarios with ≥100,000 cells to use k = 25. This parametrization prevents the global network from becoming too large, but accounts for structure being found on a larger scale for larger datasets.

Clustering on network of samples (Conos)

The Conos model constructs a joint graph of all batches in a two step process[44]. First, Conos creates pairwise connections across batches to initialize connections between identical cell types. Specifically, common principal component analysis or joint non-negative matrix factorization is used to create a joint space for cell-cell similarity computation. The cell-cell similarity scores serve as weights of the connections across datasets. Second, the number of inter-batch connections is reduced by a mutual nearest neighbor approach, and connections within a batch are down-weighted by 0.1 to account for the inherently higher cell-cell similarities of cells from the same cell type within a dataset. Ultimately, Conos returns a corrected neighborhood graph. We ran Conos (version 1.3.0) as described in the online tutorial *scanpy_integration* (https://github.com/hms-dbmi/conos/blob/master/vignettes/scanpy_integration.md). This tutorial includes HVG selection, scaling, and PCA runs per batch in Seurat. Given that Conos objects require these slots filled in order to run, we regarded the aforementioned steps as part of the Conos method. Any preprocessing combinations that we benchmark were conducted prior to the HVG selection and scaling performed within the function.

Seurat v3 (CCA and RPCA)

The original Seurat v3 data integration algorithm uses canonical correlation analysis (CCA) to construct a shared subspace of two batches[45]. The algorithm subsequently identifies mutual nearest neighbors across the two datasets, which are called “anchor points”. A projection vector is then inferred from the anchor points to integrate the two datasets in a common reference hyperplane. The same projection vectors serve to integrate new cell populations without mutual

neighbors. Integrating multiple datasets involves pairwise computation of anchor points followed by hierarchical clustering based on the distance between the datasets. The resulting tree defines the integration order to iteratively construct the common corrected data matrix.

For large, complex datasets, the creating an aligned low dimensional space via CCA can become computationally infeasible. As an alternative, reciprocal PCA (RPCA) has been suggested by the authors to improve runtimes while maintaining similar integration results. RPCA involves the projection of one dataset into the PCA space of another from which mutual nearest neighbours can be identified. The remaining steps are the same for both CCA and RPCA Seurat v3 implementations, effectively yielding two different versions of the Seurat v3 integration method.

We ran Seurat v3 using CCA and RPCA alignments (version 3.1.1) according to Seurat's integration tutorial (https://satijalab.org/seurat/v3.0/integration.html). As RPCA requires separate PCA embedding in Seurat and this only runs with prior scaling, all data input is additionally scaled by Seurat per batch for RPCA runs. After discussion with the method's authors, modifications were made to the standard parametrization to allow the passage of HVGs from Scanpy directly to the method. Seurat v3 returns a corrected gene expression or open chromatin matrix.

Harmony

The Harmony[46] algorithm initializes all datasets in PCA space along with the batch variable and alternately iterates over two complementary steps until convergence. First, it employs maximum diversity clustering, which penalizes overcorrection and pushes clusters with the same cells apart. Second, batch effects are accounted for by a linear mixture model. Thus, Harmony returns a corrected embedding. We ran Harmony (version 1.0) according to its tutorial

(http://htmlpreview.github.io/?https://github.com/immunogenomics/harmony/blob/master/docs/SeuratV3.html). As Harmony requires scaling and PCA to be run within Seurat, we regarded these steps as part of the Harmony method. Thus, any scaling or HVG selection we benchmarked occurred upstream of the scaling performed by Harmony as part of its standard workflow.

LIGER

LIGER performs integrative non-negative matrix factorization to integrate diverse batches. This approach consists of factorizing each batch expression matrix into a dataset-specific factor matrix and a shared factor matrix. The shared factor matrix is used as a joint embedding for cells across batches. We ran LIGER (version 0.4.2) with the default parameters (k = 20 and lambda = 5), as suggested in the online tutorial (https://macoskolab.github.io/liger/walkthrough_pbmc.html). This LIGER tutorial includes scaling without zero-centering and HVG selection. The custom scaling function is used as LIGER cannot accept negative input values; thus, testing our preprocessing decisions for scaling would go against the best practices for the tool. As LIGER does not give the user the flexibility to easily run alternative data scaling, we considered the LIGER scaling function to be part of the method; consequently, we only assessed the effect of HVG selection with this method.

scGen

The scGen model is a variational autoencoder paired with latent space vector arithmetics. It is designed to model single cell perturbations by projecting the data into a latent space and fitting linear vectors that explain the difference between perturbation conditions. Via vector arithmetics, data from different conditions can be mapped on top of one another in the latent space before

being projected back into the high-dimensional feature space with the perturbation effect removed. It should be noted that scGen requires cell identity annotations to fit cell-type specific batch correction vectors. Thus, scGen requires more information than other integration methods (except scANVI) to run.

Here, we regard a batch as a perturbation to use scGen to output a batch-corrected gene expression or open chromatin matrix. We ran scGen (version 1.1.5) with default parameters for 100 epochs, as suggested by the pancreas batch removal tutorial (https://nbviewer.jupyter.org/github/M0hammadL/scGen_notebooks/blob/master/notebooks/scgen_batch_removal.ipynb).

Transformer Variational Autoencoder (trVAE)

The trVAE model is a conditional variational autoencoder developed for out-of-sample prediction specifically on perturbations[47]. This method is used for data integration by using the batch label as the perturbation on which the autoencoder is conditioned during training. Conditioning on the batch label, trVAE uses the maximum mean discrepancy measure for distribution matching across batches in the first decoding layer. This layer is used as a joint embedding output for trVAE. We ran trVAE (version 0.0.1) according to the *trVAE_Haber* example notebook (https://nbviewer.jupyter.org/github/theislab/trVAE/blob/master/examples/trVAE_Haber.ipynb).

As trVAE cannot take negative values as input, we omitted scaling when testing our preprocessing decisions for this method. trVAE returns a joint embedding of cells from all batches. While trVAE can also output a corrected gene expression or open chromatin matrix, this output was not tested here as it is a reconstruction of the data with batch effect, unless specifically projected onto the same particular batch as other datasets.

SAUCIE

The SAUCIE model is an autoencoder that enables multitask learning for standard single cell analyses including clustering, visualization and batch effect removal[48]. It uses a maximal mean discrepancy (MMD) correction to match distributions between batches and thus remove batches effects in the data. SAUCIE has been suggested as an analysis approach for both CyTOF and scRNA-seq data.

In the absence of a tutorial, we ran SAUCIE (version 2020-06-04) according to the example notebook provided by the authors in the repository (https://colab.research.google.com/github/KrishnaswamyLab/SingleCellWorkshop/blob/master/exercises/Deep_Learning/notebooks/02_Answers_Exploratory_analysis_of_single_cell_data_with_SAUCIE.ipynb). In accordance with the above notebook, we PCA transform the data and select the first 100 components before running SAUCIE, and inverse transform the PCA output to get back a feature space representation of the data upon integration. Thus, SAUCIE outputs both a joint embedding and a corrected gene expression or open chromatin matrix.

DESC

DESC[49] is an iterative clustering-based deep neural network built on an autoencoder latent representation. It does not require batch labels as input, but they can be used for scaling by batch before training the neural network. DESC's central principle is that the network will learn the batch effects while iteratively improving cluster assignments. Thus, DESC is principally designed to output an optimized clustering of cells, yet it also outputs the embedding which it uses for this cluster assignment. Here, we ignored the clustering output, but used this embedding output as a joint embedding output from DESC.

We scaled the data by batch labels with the function provided by DESC and trained the model on the batch-scaled data. We ran DESC according to parameterizations suggested by the author (https://github.com/eleozzr/desc/issues/28) rather than the tutorial (https://eleozzr.github.io/desc/tutorial.html). As DESC is designed for batch-removed clustering, it is particularly reliant on an optimal clustering resolution parameterization. Thus, the lack of input parameter tuning in this benchmarking study may particularly affect DESC integration evaluation.

Supplementary Figures

Erythrocyte development trajectories on immune (human)

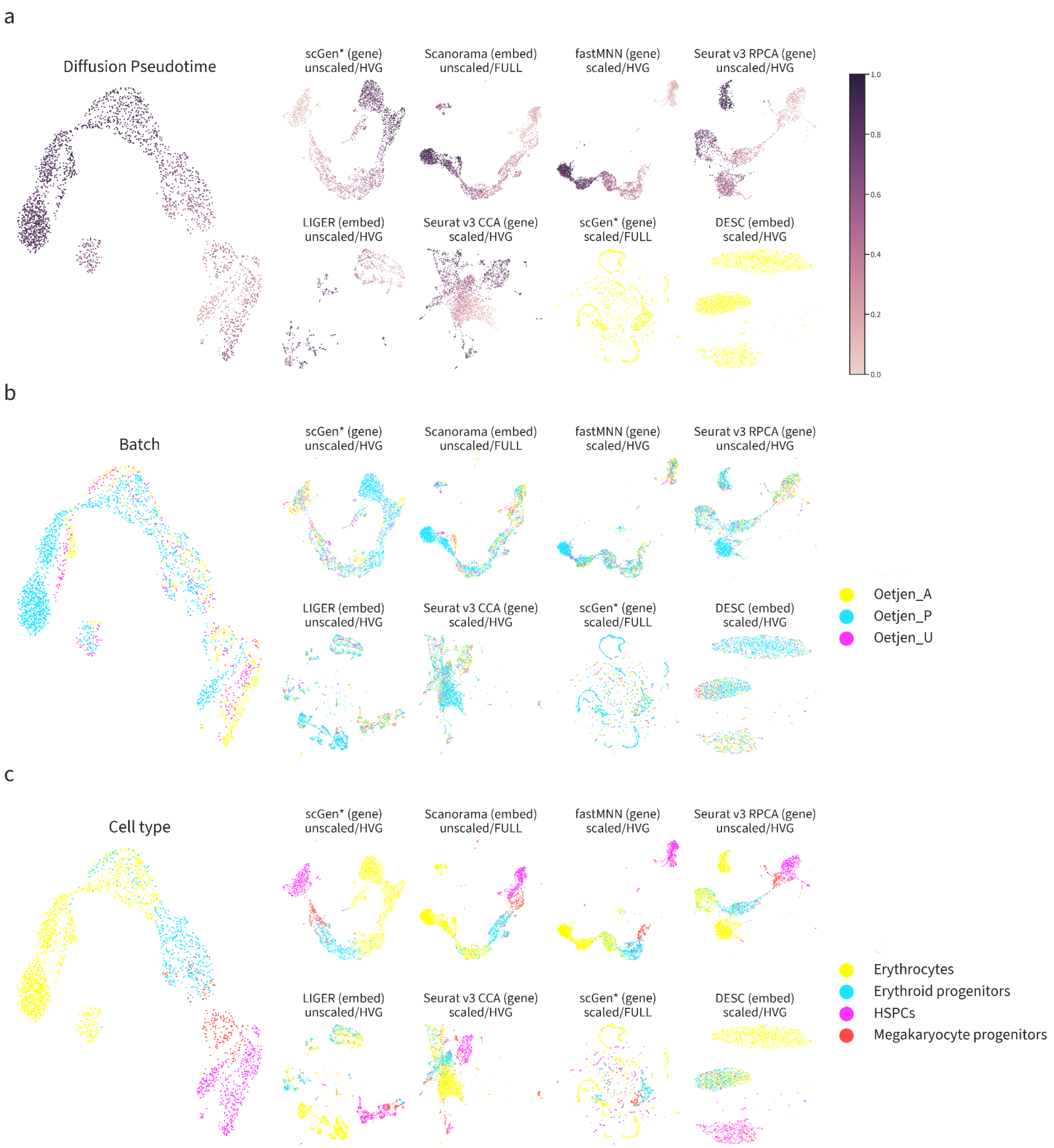

Supplementary Figure 1: Trajectories of the best and worst performers on the immune cell human integration task ordered by trajectory score on the set of cells belonging to

the erythrocyte lineage. The plots show UMAP layouts for the unintegrated data (left), the top 4 performers (upper rows a, b and c), and the worst 4 performers (lower rows a, b and c). Plots are colored by (a) diffusion pseudotime, (b) batch labels, and (c) cell identity annotations. In cases where it wasn't possible to compute a trajectory due to disconnected clusters, all cells are colored yellow in (a)

Benchmarking metric results

Immune (human)

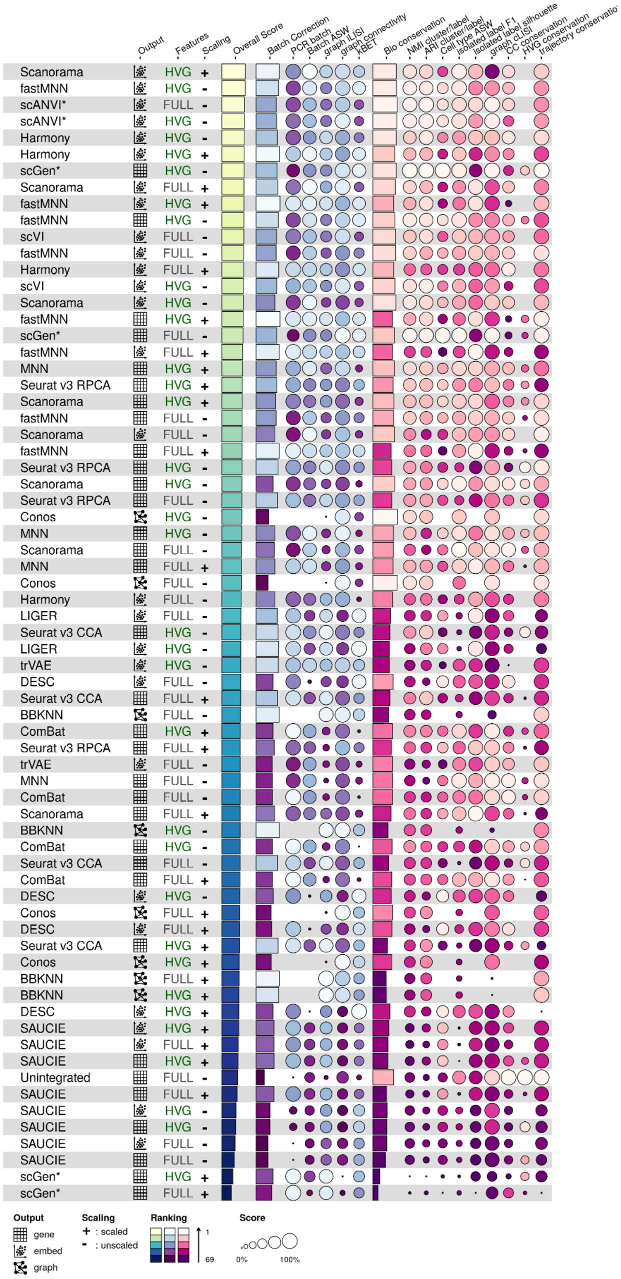

Supplementary Figure 2: Overview of benchmarking results by overall score for the human immune cell task. Metrics are divided into batch correction (blue, purple) and bio conservation (pink) categories. Overall scores are computed by a 40:60 weighted mean of these category scores (see **Methods** for further visualization details).

Immune (human/mouse)

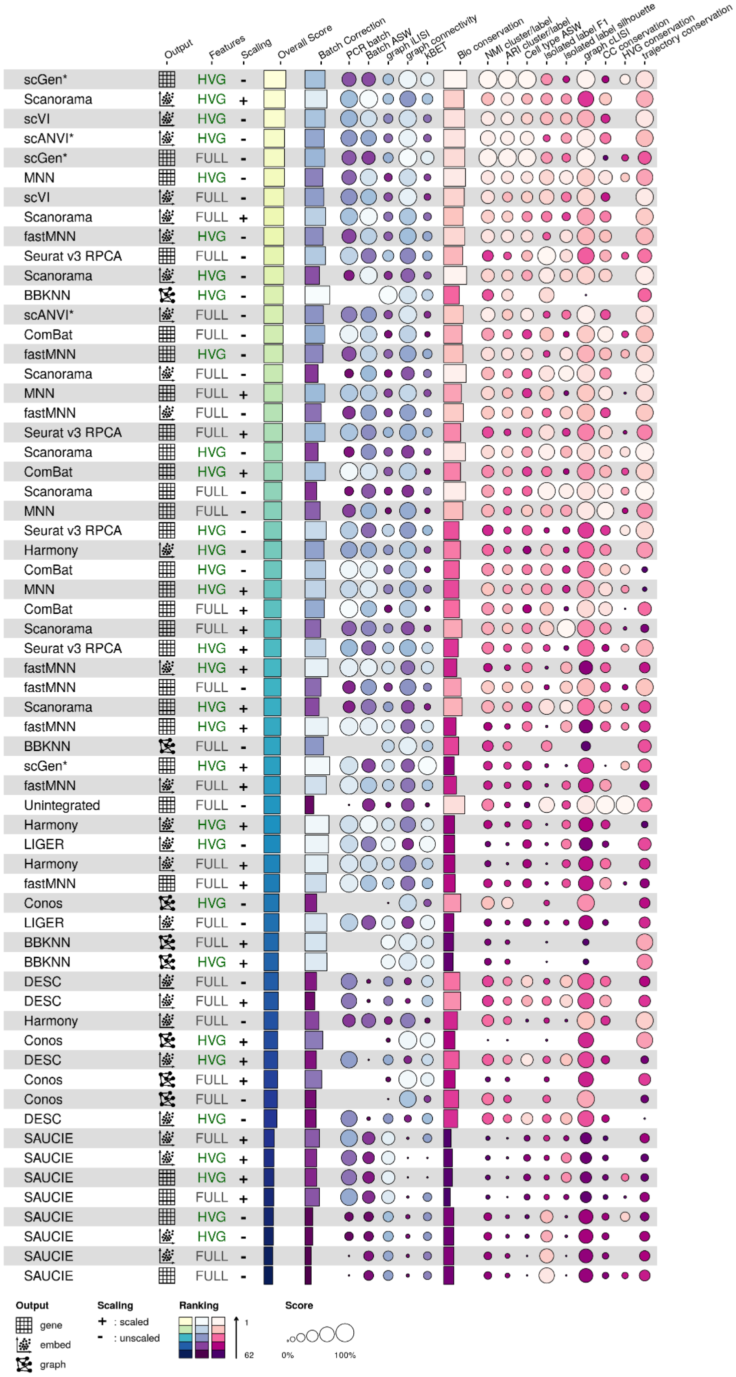

Supplementary Figure 3: Overview of benchmarking results by overall score for the human/mouse immune cell task. Metrics are divided into batch correction (blue, purple) and bio conservation (pink) categories. Overall scores are computed by a 40:60 weighted mean of these category scores (see **Methods** for further visualization details). Methods that failed to run are omitted.

Simulation 1

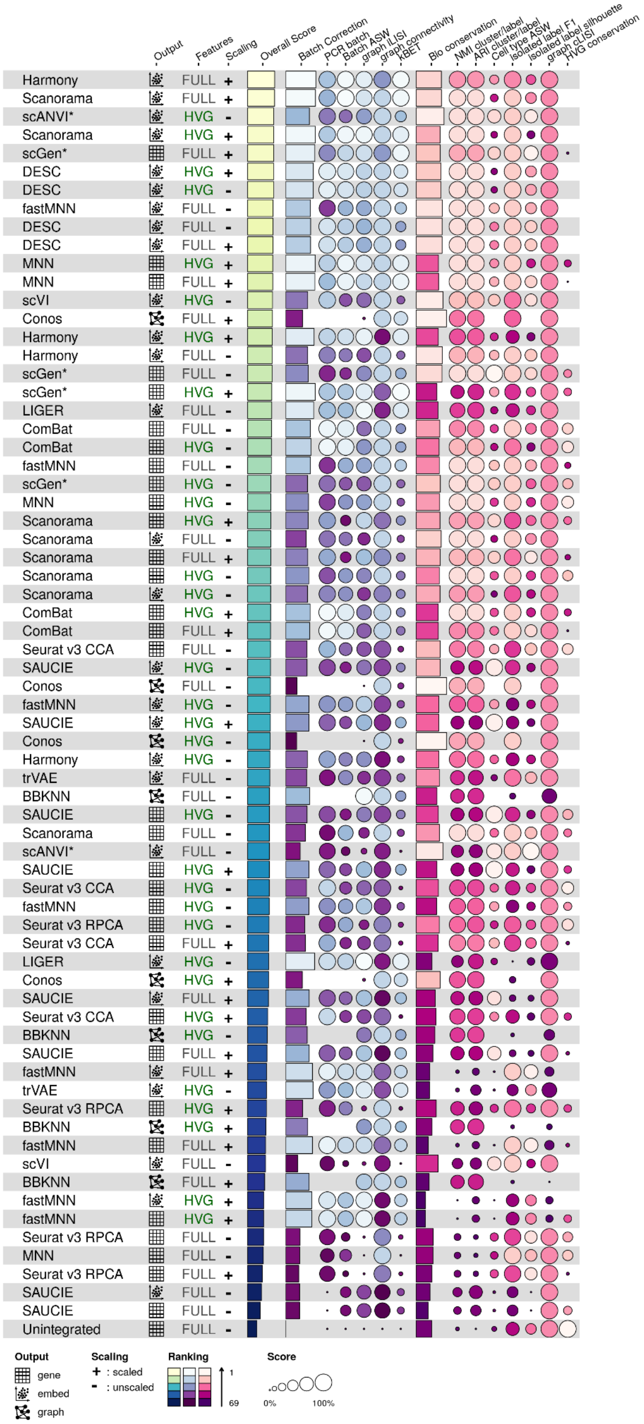

Supplementary Figure 4: Overview of benchmarking results by overall score for the simulation 1 task. Metrics are divided into batch correction (blue, purple) and bio conservation (pink) categories. Overall scores are computed by a 40:60 weighted mean of these category scores (see **Methods** for further visualization details).

Simulation 2

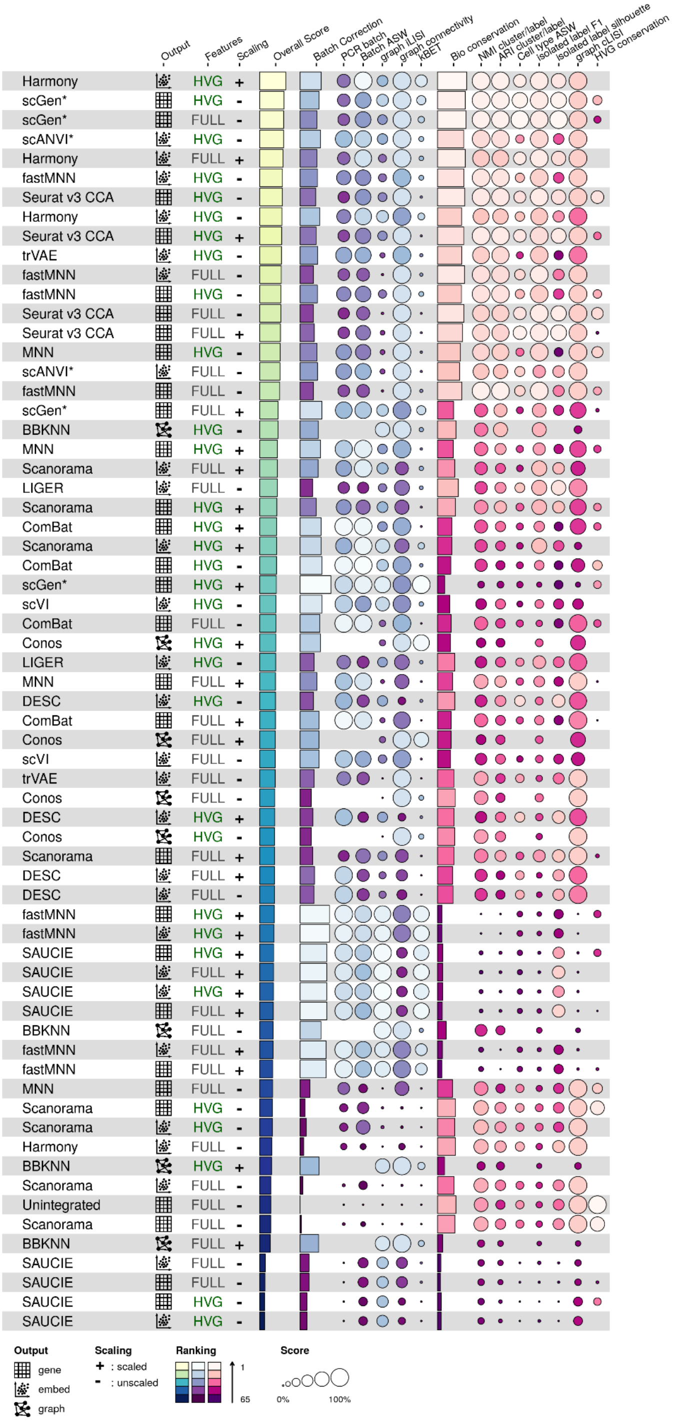

Supplementary Figure 5: Overview of benchmarking results by overall score for the simulation 2 task. Metrics are divided into batch correction (blue, purple) and bio conservation (pink) categories. Overall scores are computed by a 40:60 weighted mean of these category scores (see **Methods** for further visualization details).

Pancreas

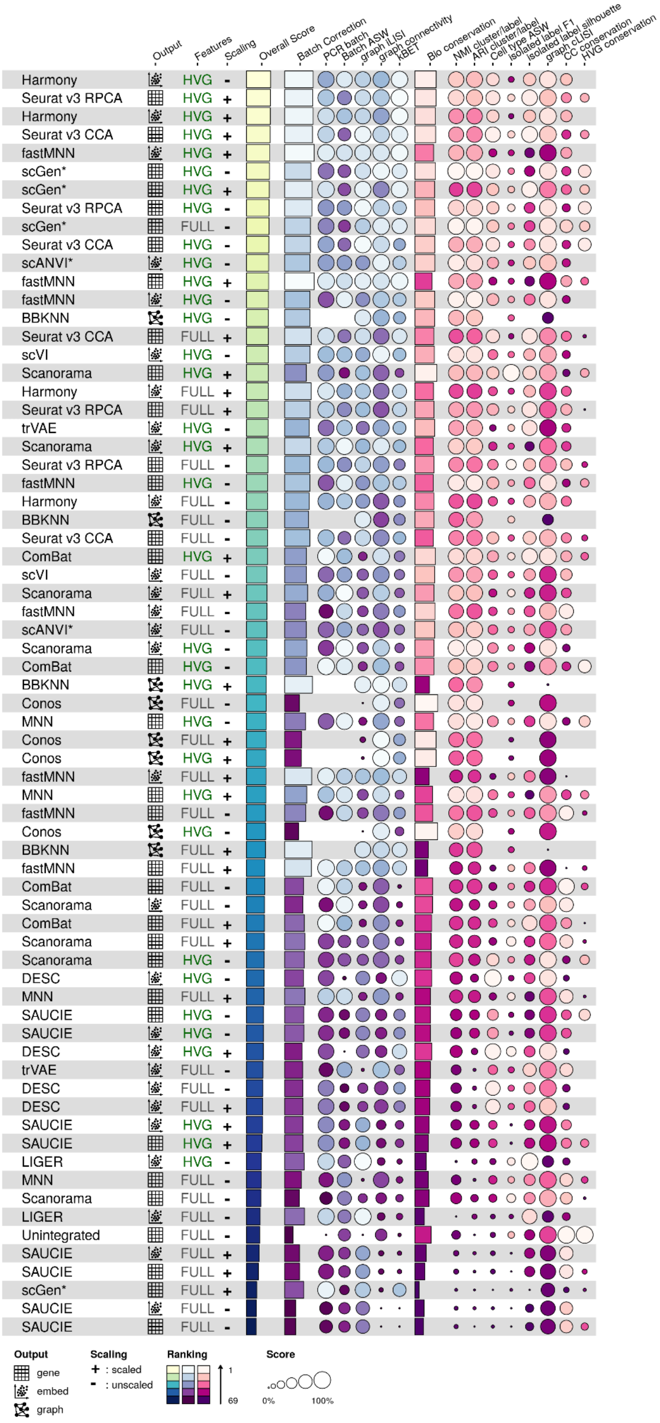

Supplementary Figure 6: Overview of benchmarking results by overall score for the pancreas task. Metrics are divided into batch correction (blue, purple) and bio conservation (pink) categories. Overall scores are computed by a 40:60 weighted mean of these category scores (see **Methods** for further visualization details).

Lung

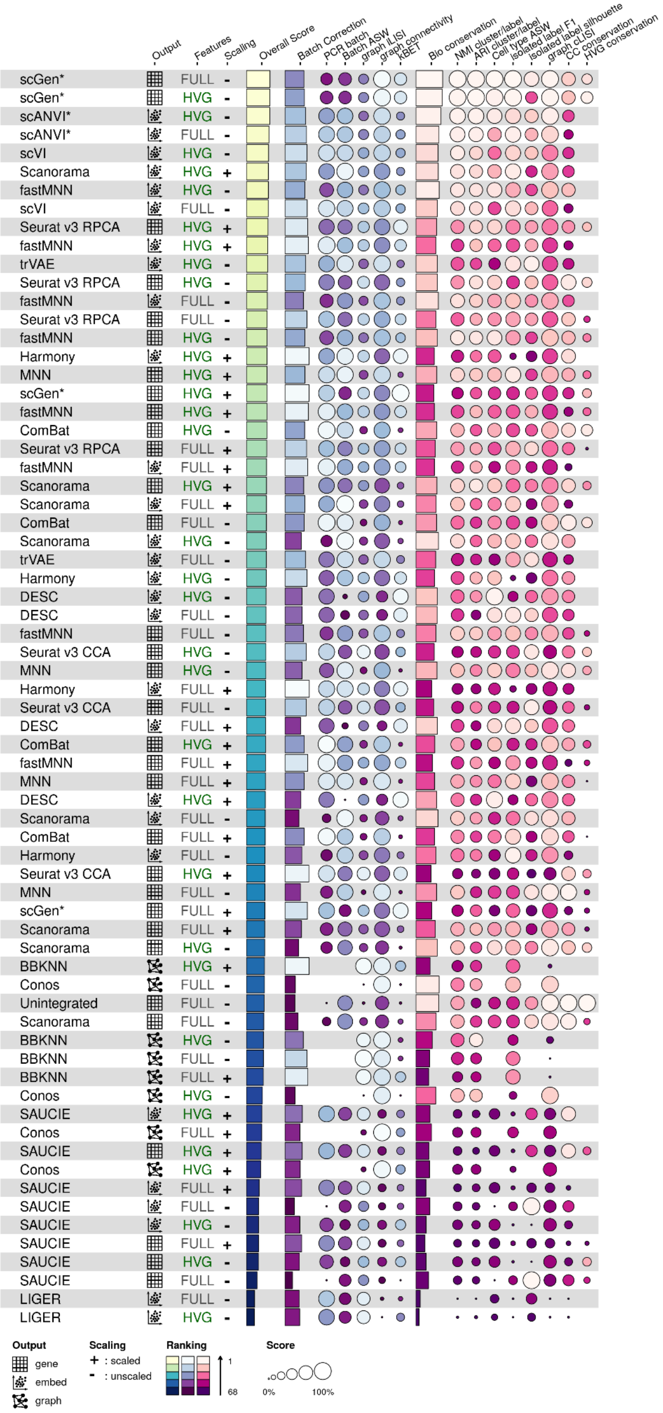

Supplementary Figure 7: Overview of benchmarking results by overall score for the lung atlas task. Metrics are divided into batch correction (blue, purple) and bio conservation (pink) categories. Overall scores are computed by a 40:60 weighted mean of these category scores (see **Methods** for further visualization details). Methods that failed to run are omitted.

Mouse brain

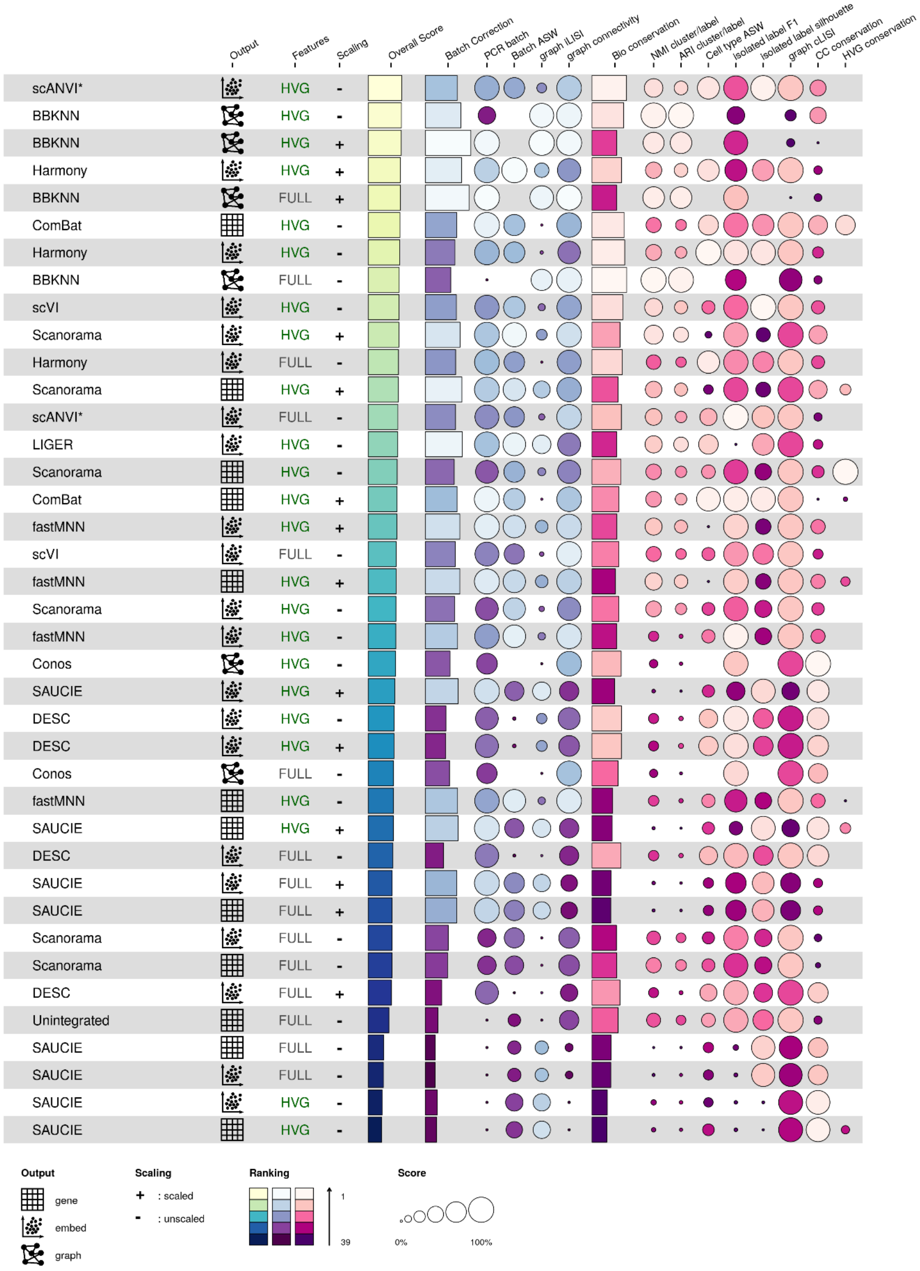

Supplementary Figure 8: Overview of benchmarking results by overall score for the mouse brain RNA task. Metrics are divided into batch correction (blue, purple) and bio conservation (pink) categories. Overall scores are computed by a 40:60 weighted mean of these category scores (see **Methods** for further visualization details). Methods that failed to run are omitted. Note that kBET was not run on this task due to computational limitations.

Embeddings

Immune (human)

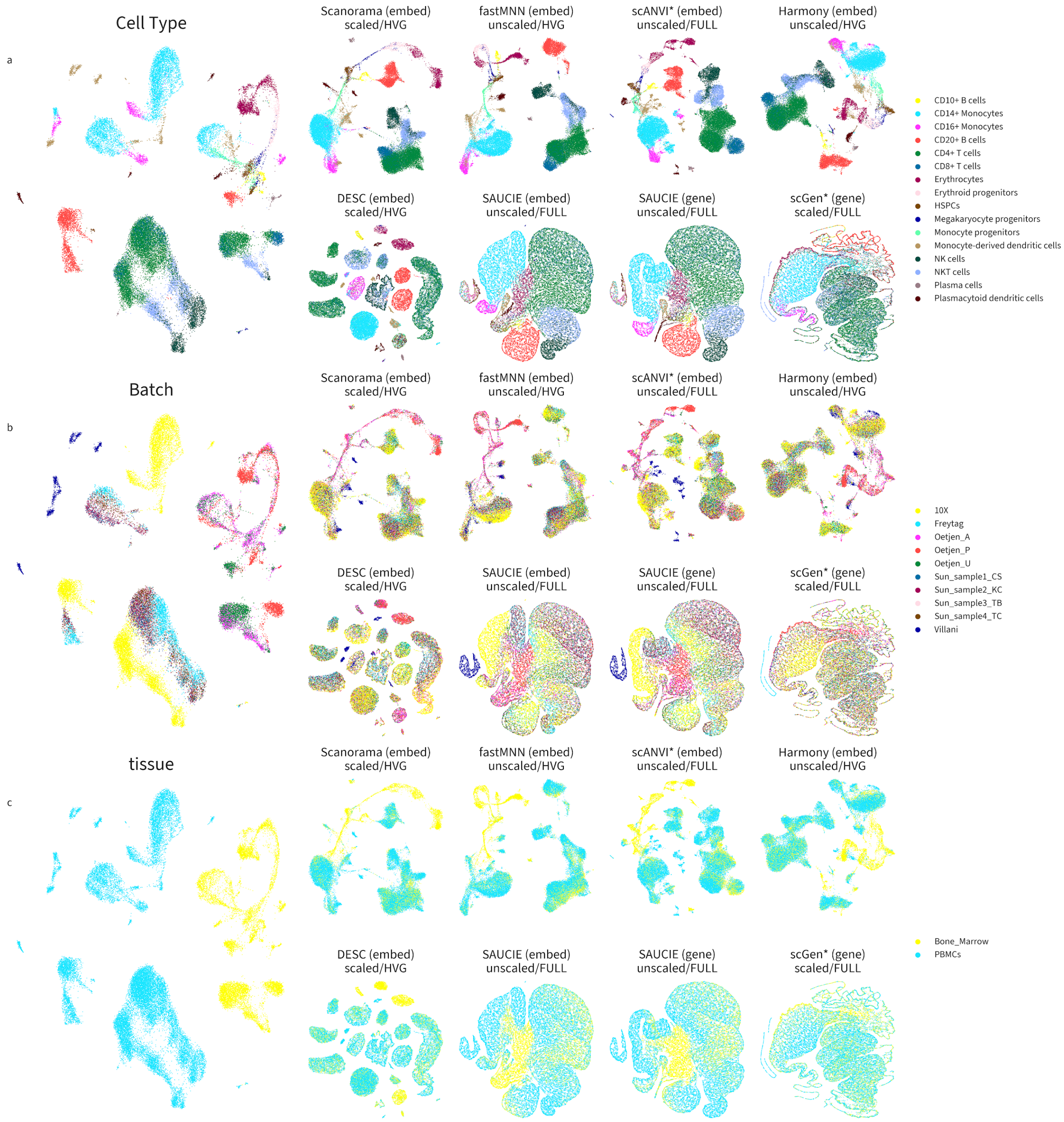

Supplementary Figure 9: Visualization of the best and worst performers on the immune cell human data. The plots show UMAP layouts for the unintegrated data (left), the top 4 performers (upper rows a, b and c), and the worst 4 performers (lower rows a, b and c). Plots are colored by (a) cell identity annotations, (b) batch labels, and (c) tissues.

Immune (human/mouse)

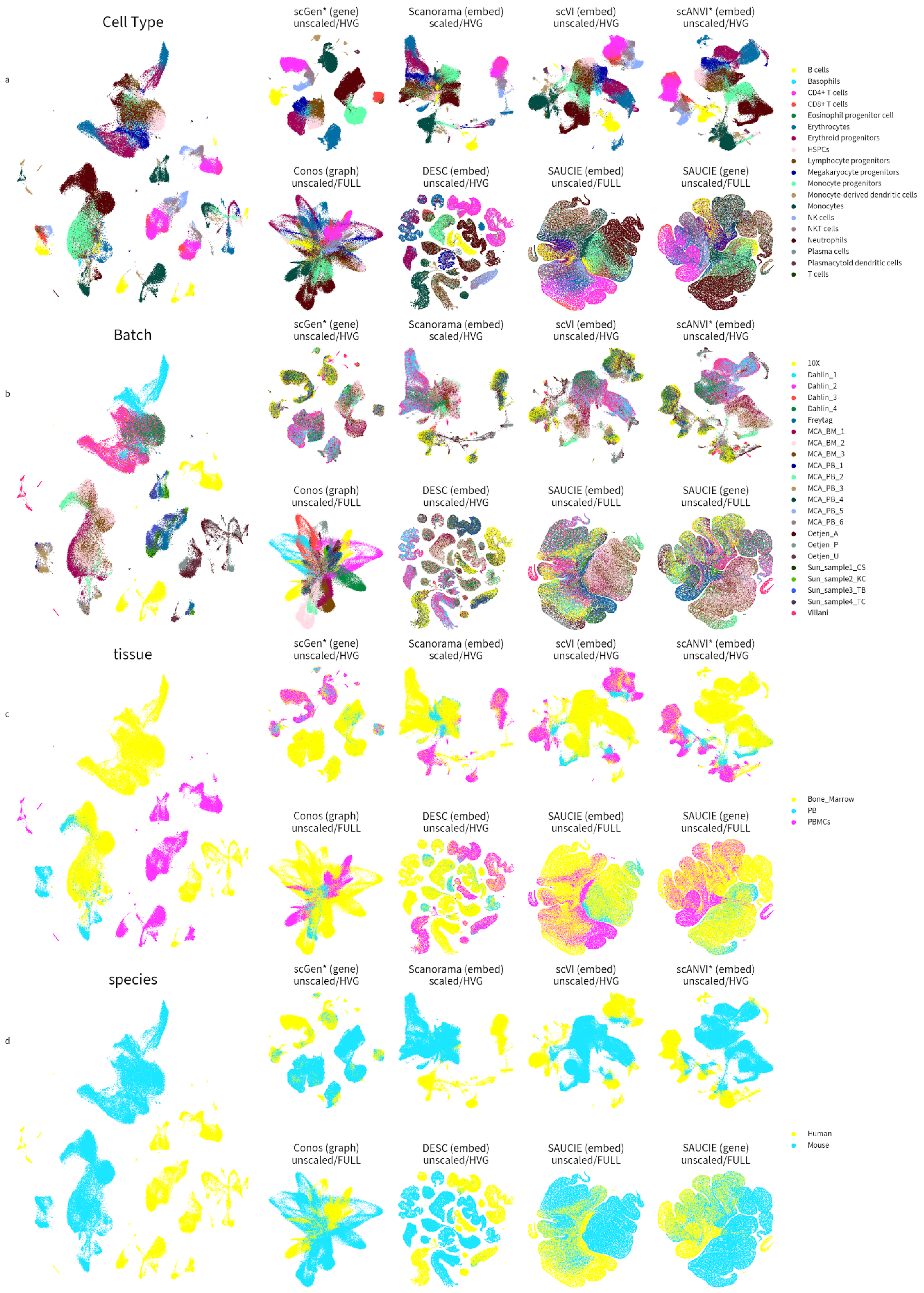

Supplementary Figure 10: **Visualization of the best and worst performers on the immune cell human mouse integration task.** The plots show Force Atlas 2 (Conos) and UMAP (all other methods) layouts for the unintegrated data (left), the top 4 performers (upper rows a, b, c and d), and the worst 4 performers (lower rows a, b, c and d). Plots are colored by (a) cell identity annotations, (b) batch labels, (c) tissues, and (d) species. Tissues are abbreviated as: PB - peripheral blood, PBMCs - peripheral blood mononuclear cells.

Simulation 1

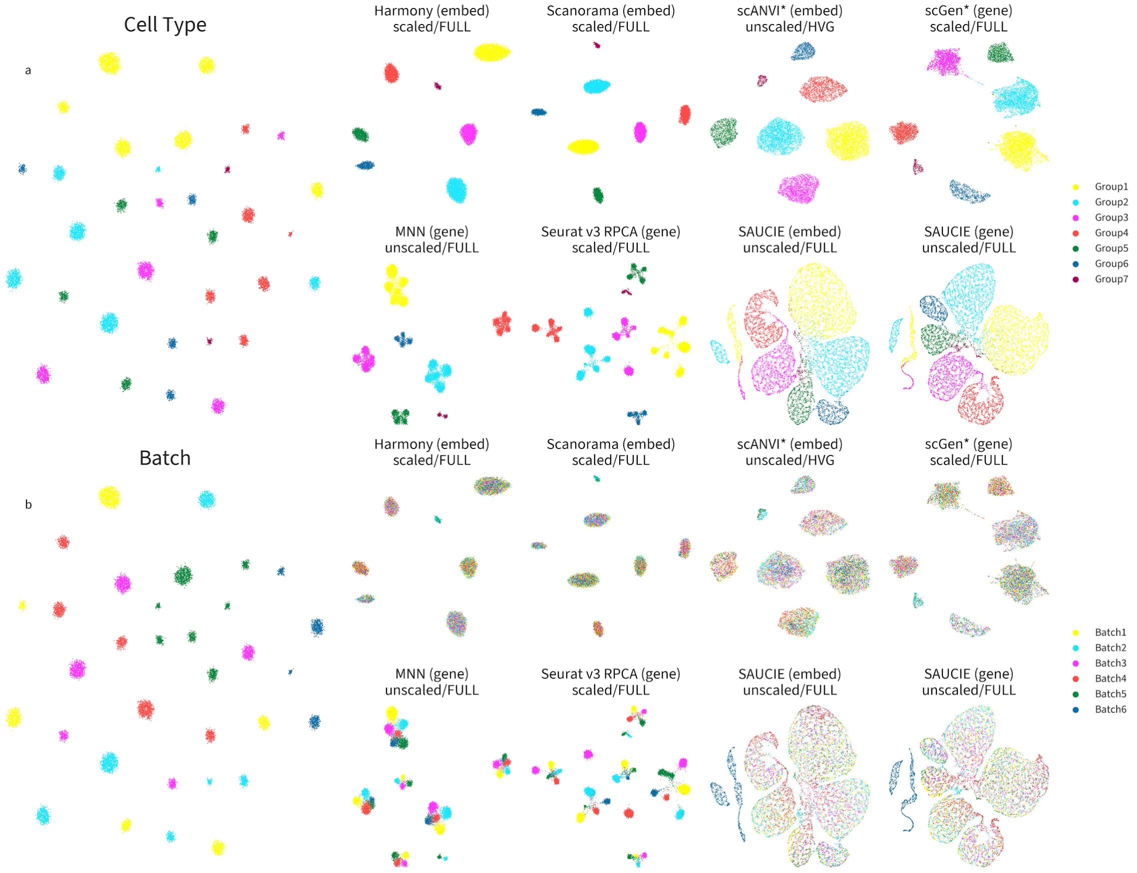

Supplementary Figure 11: Visualization of the best and worst performers on the simulation 1 task. The plots show Force Atlas 2 (Conos) and UMAP (all other methods) layouts for the unintegrated data (left), the top 4 performers (upper rows a and b), and the worst 4 performers (lower rows a and b). Plots are colored by (a) cell identity annotations, and (b) batch labels.

Simulation 2

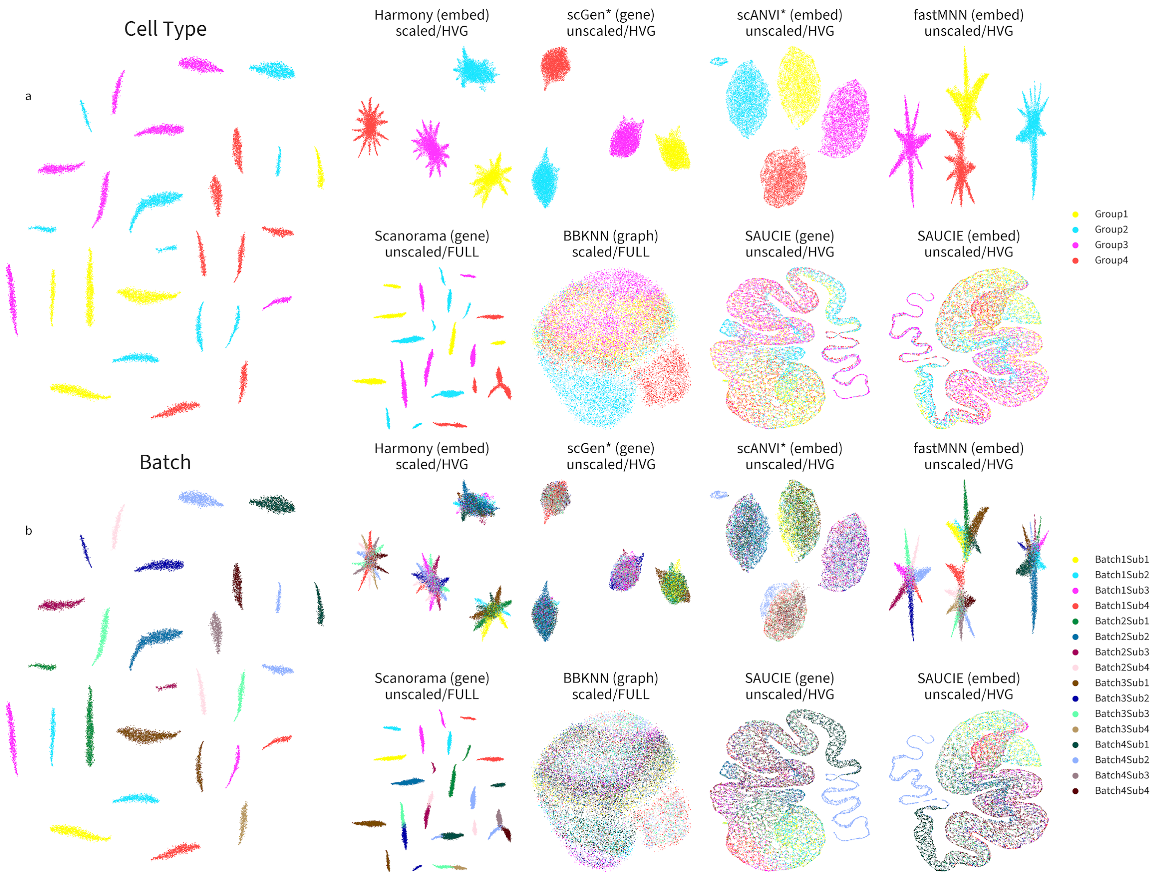

Supplementary Figure 12: Visualization of the best and worst performers on the simulation 2 task. The plots show Force Atlas 2 (Conos) and UMAP (all other methods) layouts for the unintegrated data (left), the top four performers (upper rows a and b), and the worst four performers (lower rows a and b). Plots are colored by (a) cell identity annotations, and (b) batch labels.

Pancreas

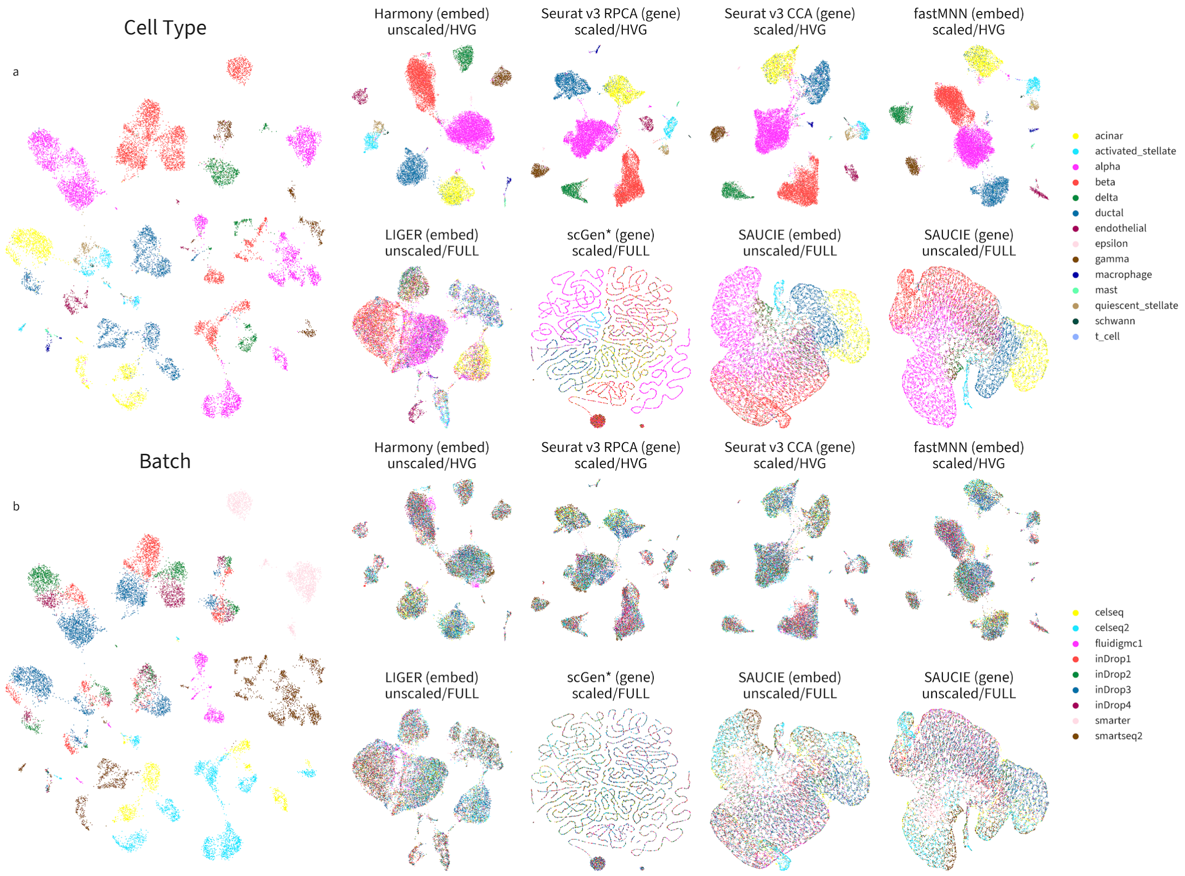

Supplementary Figure 13: Visualization of the best and worst performers on the pancreas integration task. The plots show Force Atlas 2 (Conos) and UMAP (all other methods) layouts for the unintegrated data (left), the top 4 performers (upper rows a and b), and the worst 4 performers (lower rows a and b). Plots are colored by (a) cell identity annotations, and (b) batch labels.

Lung

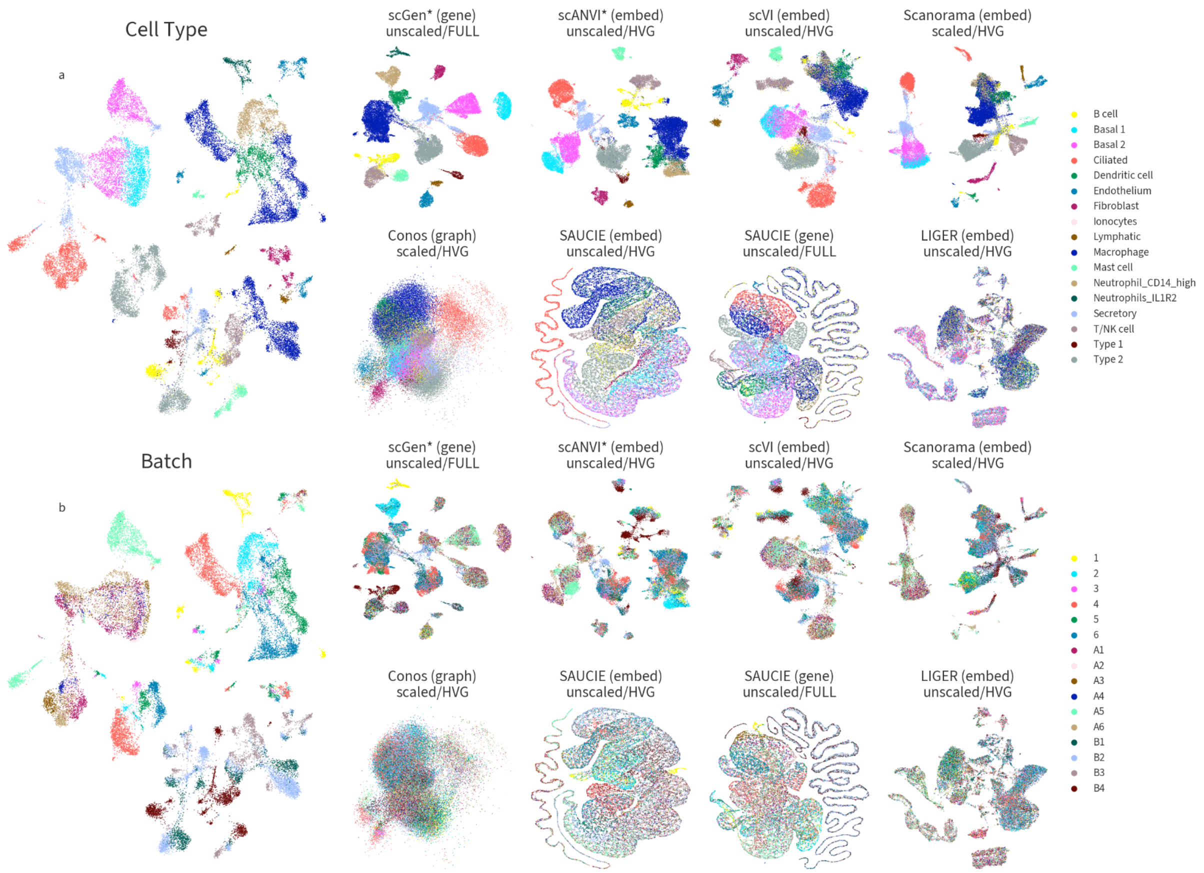

Supplementary Figure 14: Visualization of the best and worst performers on the lung atlas integration task. The plots show Force Atlas 2 (Conos) and UMAP (all other methods) layouts for the unintegrated data (left), the top 4 performers (upper rows a and b), and the worst 4 performers (lower rows a and b). Plots are colored by (a) cell identity annotations, and (b) batch labels.

Mouse brain

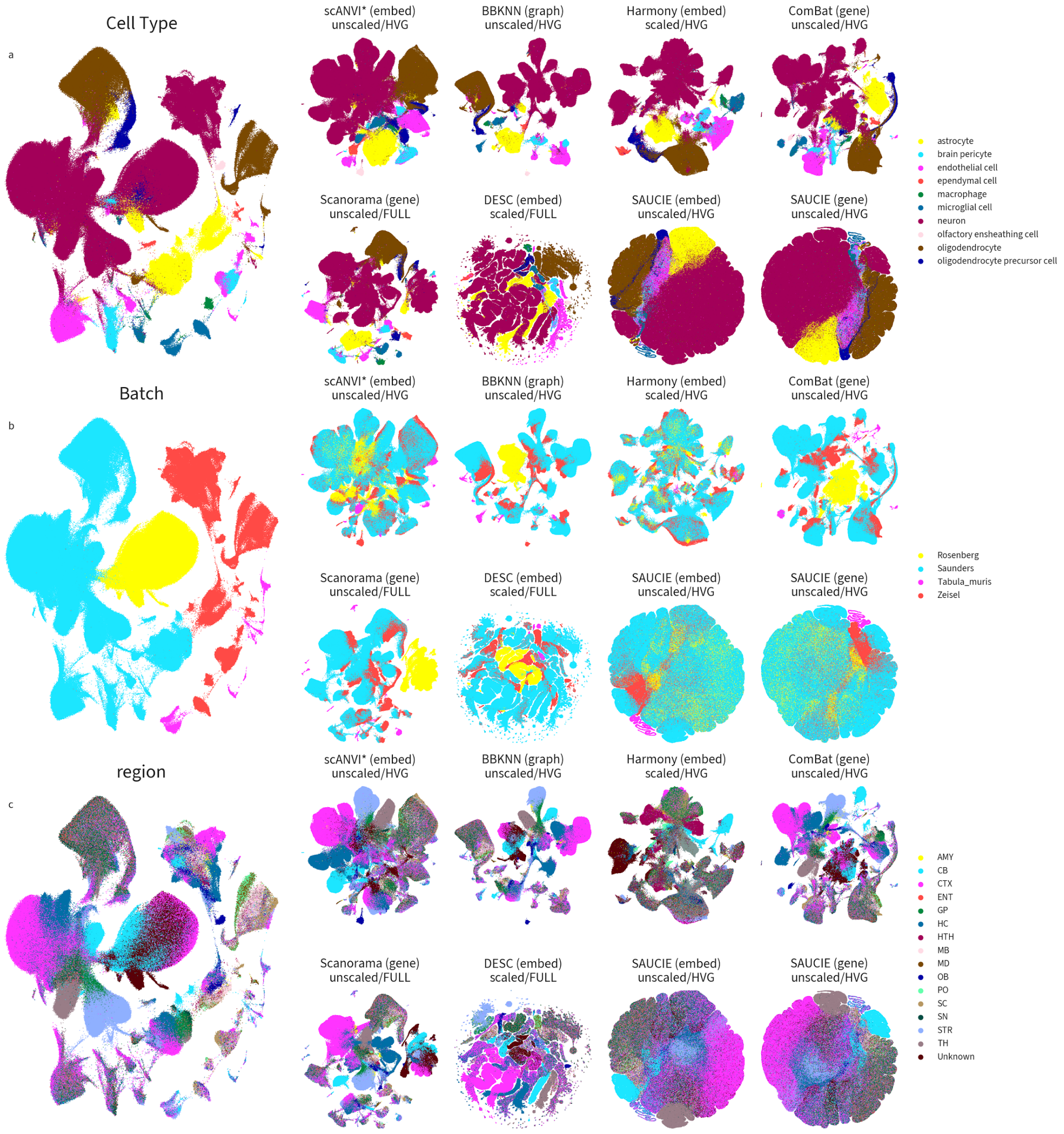

Supplementary Figure 15: Visualization of the best and worst performers on the mouse brain RNA integration task. The plots show Force Atlas 2 (Conos) and UMAP (all other methods) layouts for the unintegrated data (left), the top 4 performers (upper rows a, b, and c), and the worst 4 performers (lower rows a, b, and c). Plots are colored by (a) cell identity annotations, (b) batch labels, and (c) brain regions. Brain regions are abbreviated as: AMY - amygdala, HC - hippocampus, TH - thalamus, HTH - hypothalamus, CTX - cortex, OB - olfactory bulb, STR - striatum, CB - cerebellum, MB - midbrain, MD - medulla, SN - substantia nigra, ENT - entopeduncular nucleus, GP - globus pallidus and nucleus basalis, PO - pons, and SC - spinal cord (unknown regions could not be inferred in the original publication of Rosenberg *et al.*[21]).

Trajectories across species

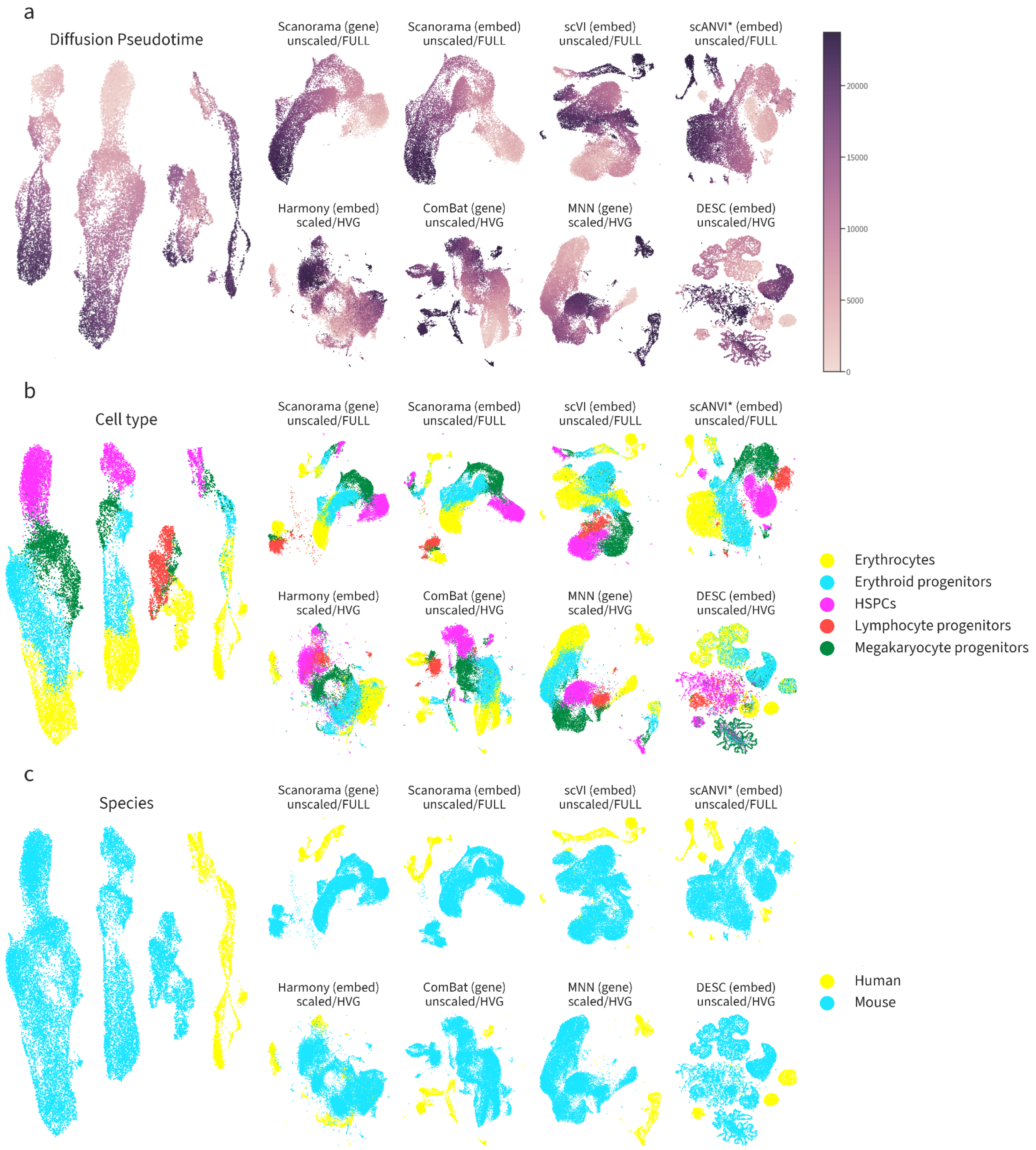

Supplementary Figure 16: Visualization of the best and worst performers on the immune cell human mouse integration task ordered by trajectory score on the set of cells belonging to the erythrocyte lineage. The plots show UMAP layouts for the unintegrated data (left), the top 4 performers (upper rows a, b and c), and the worst 4 performers (lower rows a, b and c). Plots are colored by (a) diffusion pseudotime, (b) cell identity annotations, and (c) species. Plot (a) only shows the cells of the largest connected component, as the trajectory was only computed on those.

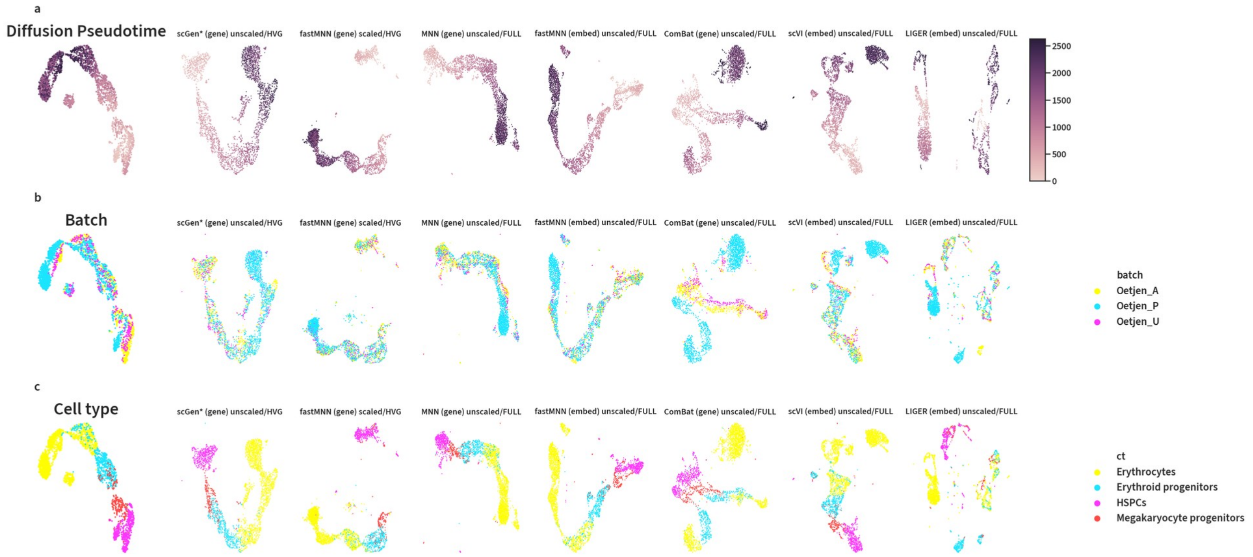

Supplementary Figure 17: Visualization of additional methods on the immune cell human integration task ordered by trajectory score on the set of cells belonging to the erythrocyte lineage. The plots show UMAP layouts for the unintegrated data (left), and integrated data in the remaining plots. Plots are colored by (a) diffusion pseudotime, (b) batch, and (c) cell identity annotations.

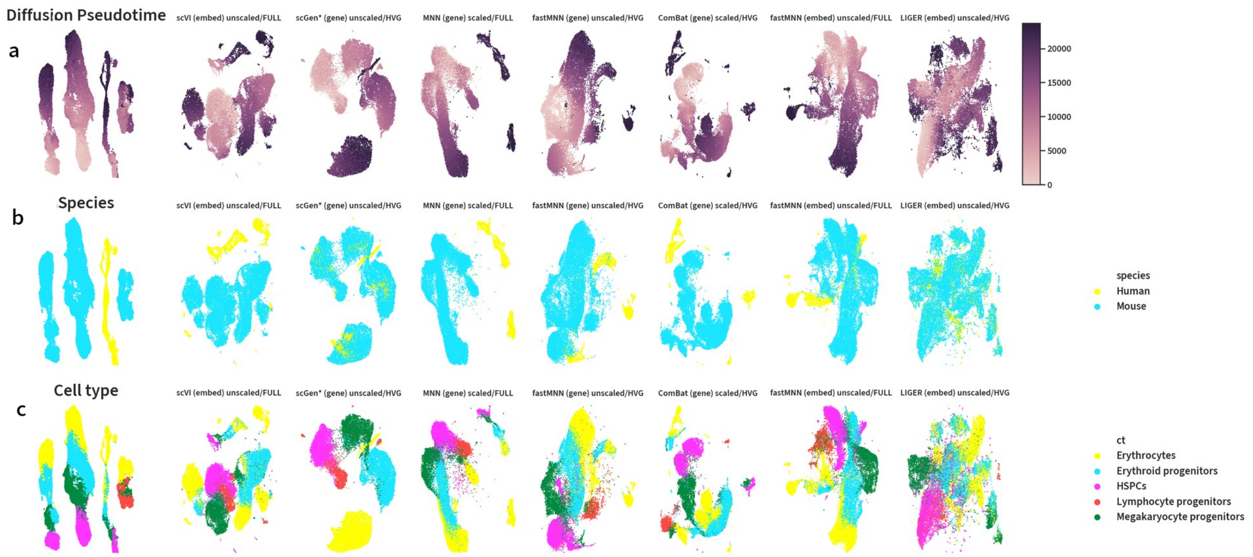

Supplementary Figure 18: Visualization of additional methods on the immune cell human mouse integration task ordered by trajectory score on the set of cells belonging to the erythrocyte lineage. The plots show UMAP layouts for the unintegrated data (left), and integrated data in the remaining plots. Plots are colored by (a) diffusion pseudotime, (b) species, and (c) cell identity annotations.

Benchmarking metric results for ATAC tasks

Mouse brain (ATAC) - windows

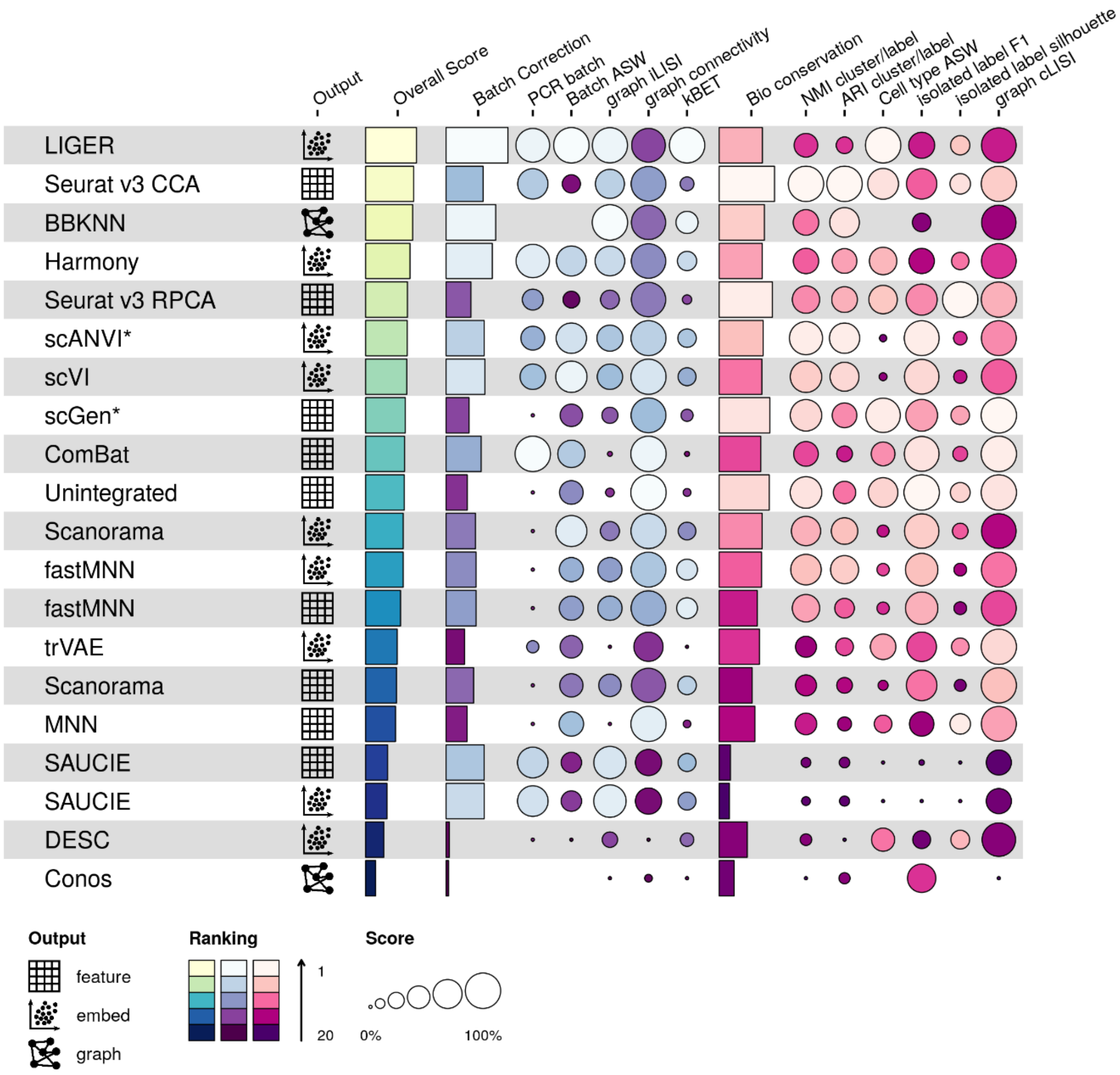

Supplementary Figure 19: Benchmarking results for the small mouse brain task using 5k-bp windows based on scATAC-seq. Metrics are divided into batch correction (blue, purple) and bio conservation (pink) categories (see **Methods** for further visualization details). Overall scores are computed by a 40:60 weighted mean of these category scores. Methods that failed to run are omitted.

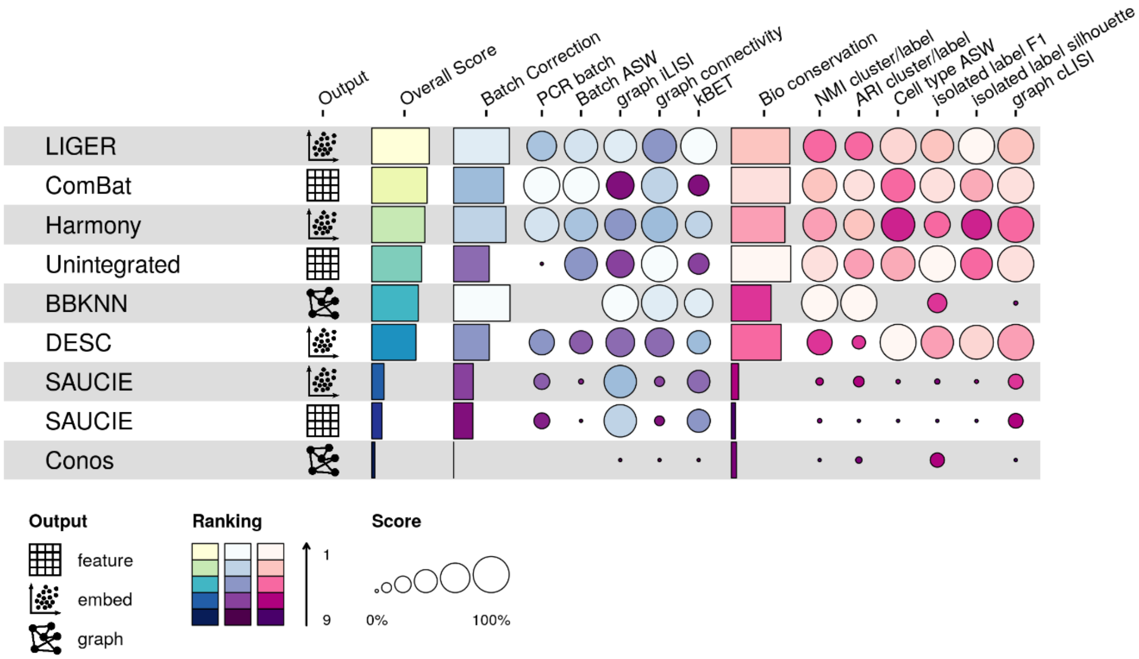

Supplementary Figure 20: Benchmarking results for the large mouse brain task using 5k-bp windows based on scATAC-seq. Metrics are divided into batch correction (blue, purple) and bio conservation (pink) categories (see **Methods** for further visualization details). Overall scores are computed by a 40:60 weighted mean of these category scores. Methods that failed to run are omitted.

Mouse brain (ATAC) - peaks

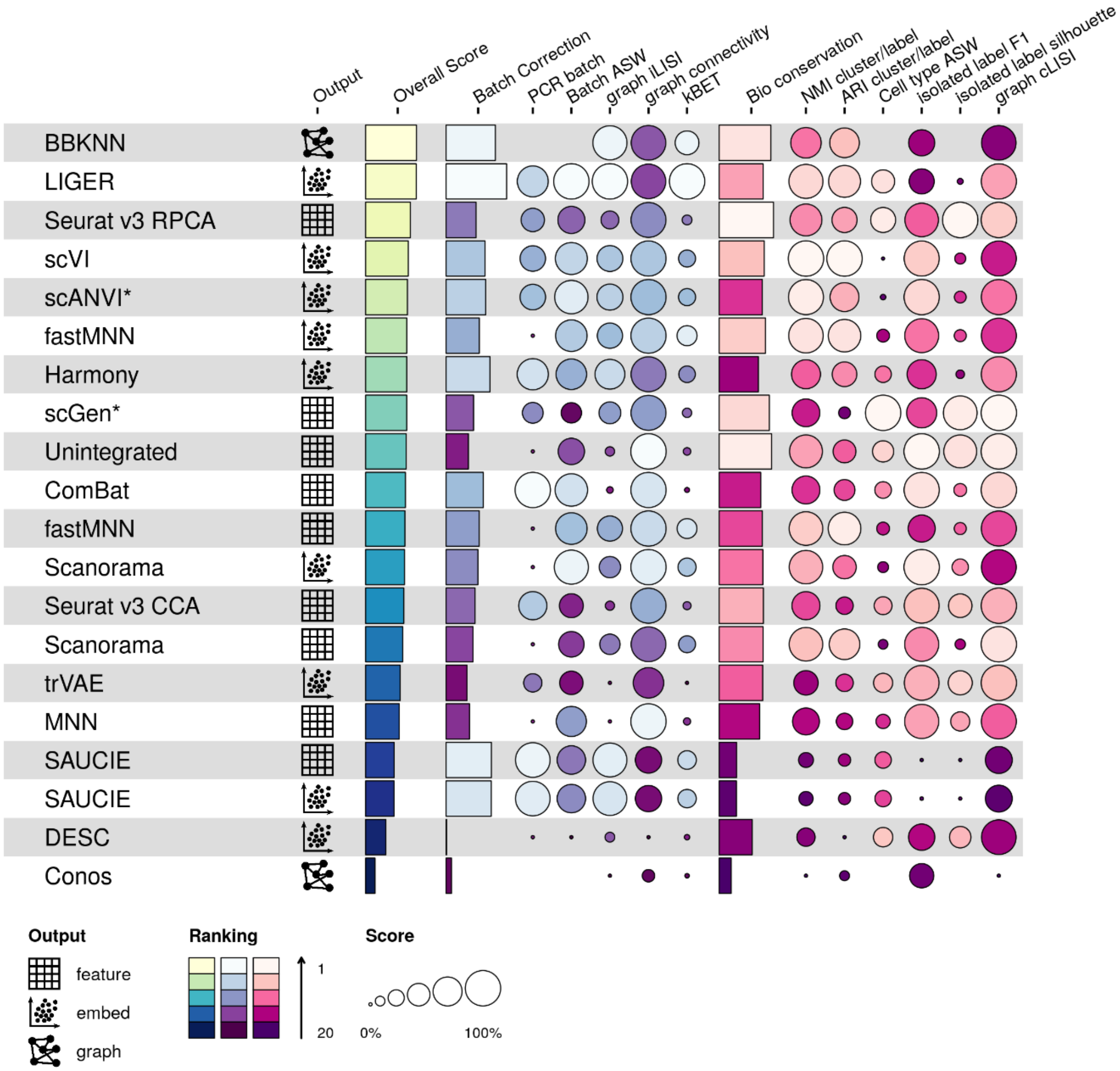

Supplementary Figure 21: Benchmarking results for the small mouse brain task using peaks based on scATAC-seq. Metrics are divided into batch correction (blue, purple) and bio conservation (pink) categories (see **Methods** for further visualization details). Overall scores are computed by a 40:60 weighted mean of these category scores. Methods that failed to run are omitted.

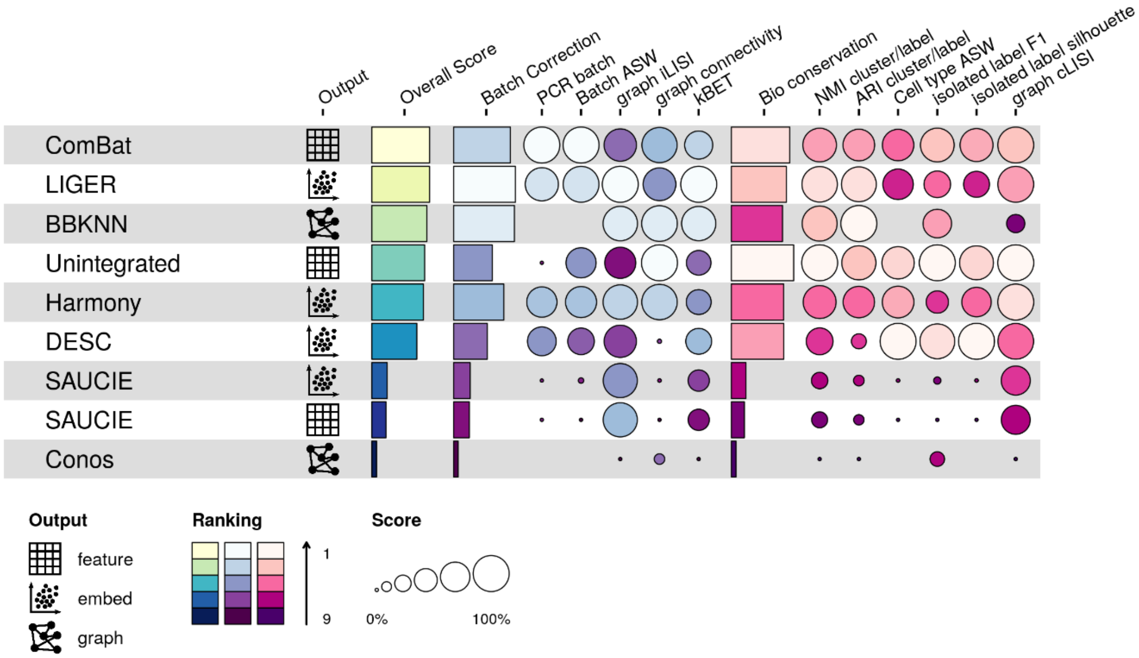

Supplementary Figure 22: Benchmarking results for the large mouse brain task using peaks based on scATAC-seq. Metrics are divided into batch correction (blue, purple) and bio conservation (pink) categories (see **Methods** for further visualization details). Overall scores are computed by a 40:60 weighted mean of these category scores. Methods that failed to run are omitted.

Mouse brain (ATAC) - gene activity

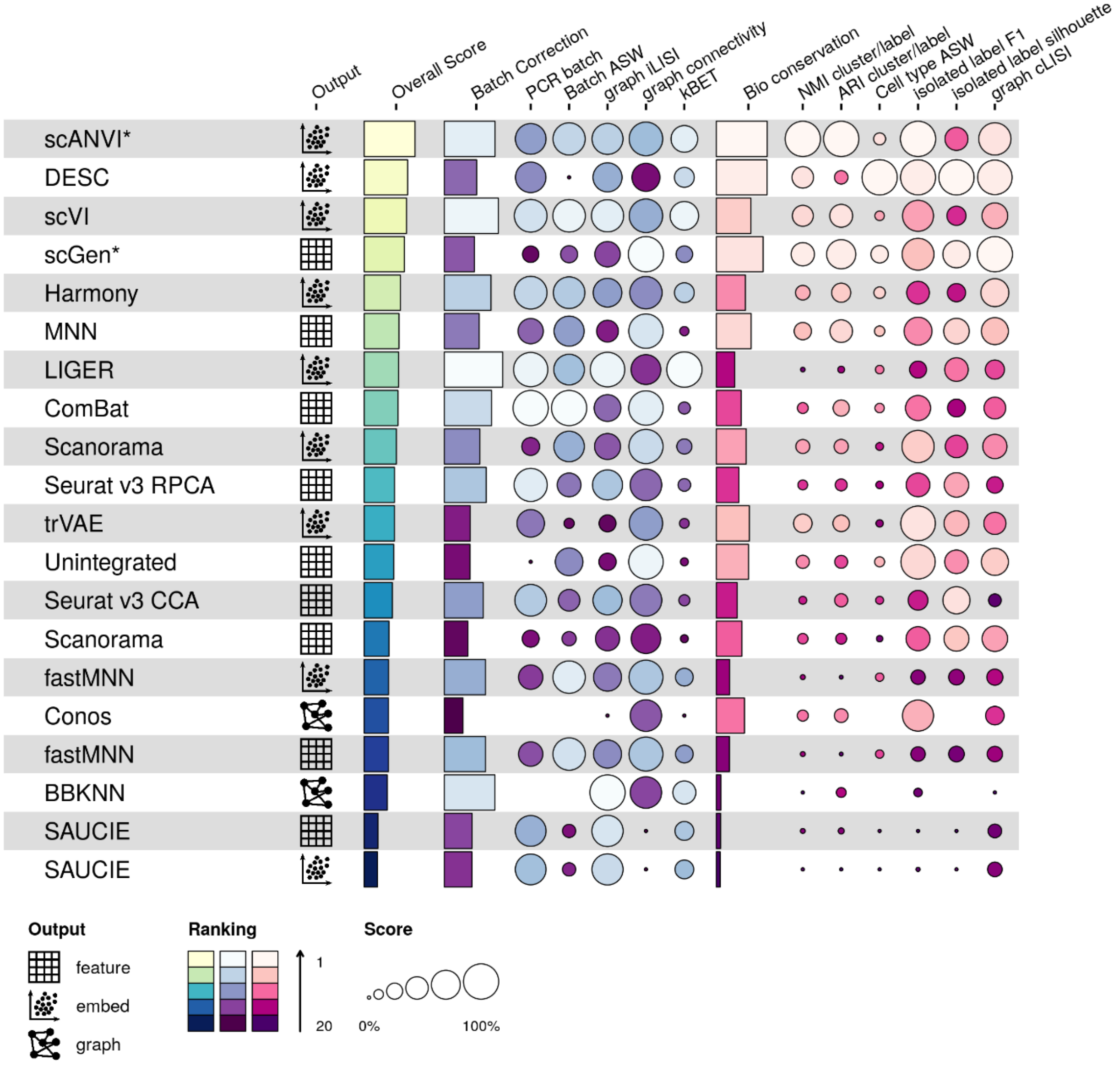

Supplementary Figure 23: Benchmarking results for the small mouse brain task using gene activity based on scATAC-seq. Metrics are divided into batch correction (blue, purple) and bio conservation (pink) categories (see **Methods** for further visualization details). Overall scores are computed by a 40:60 weighted mean of these category scores. Methods that failed to run are omitted.

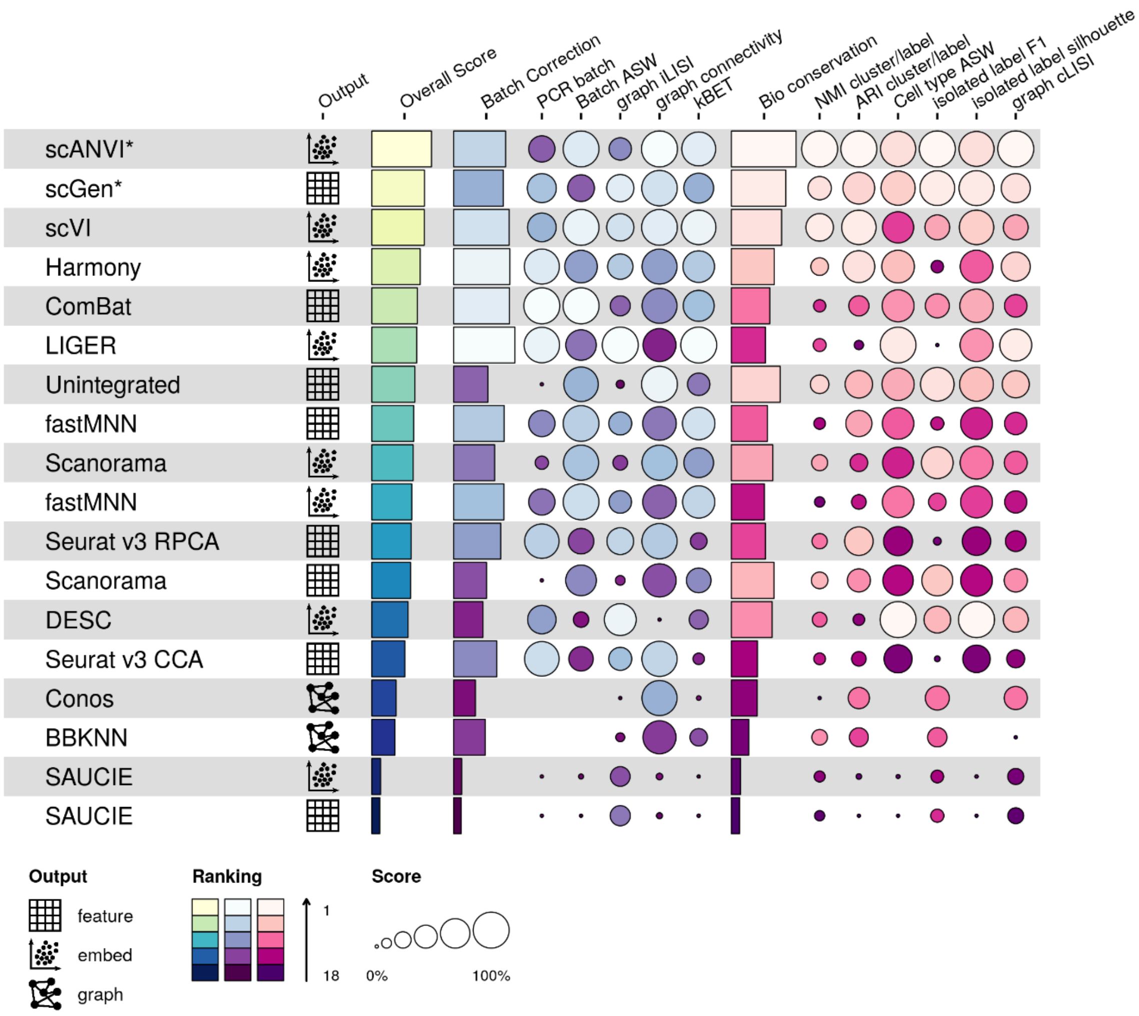

Supplementary Figure 24: Benchmarking results for the large mouse brain task using gene activity based on scATAC-seq. Metrics are divided into batch correction (blue, purple) and bio conservation (pink) categories (see **Methods** for further visualization details). Overall scores are computed by a 40:60 weighted mean of these category scores. Methods that failed to run are omitted.

Embeddings for ATAC tasks

Mouse brain (ATAC) - windows

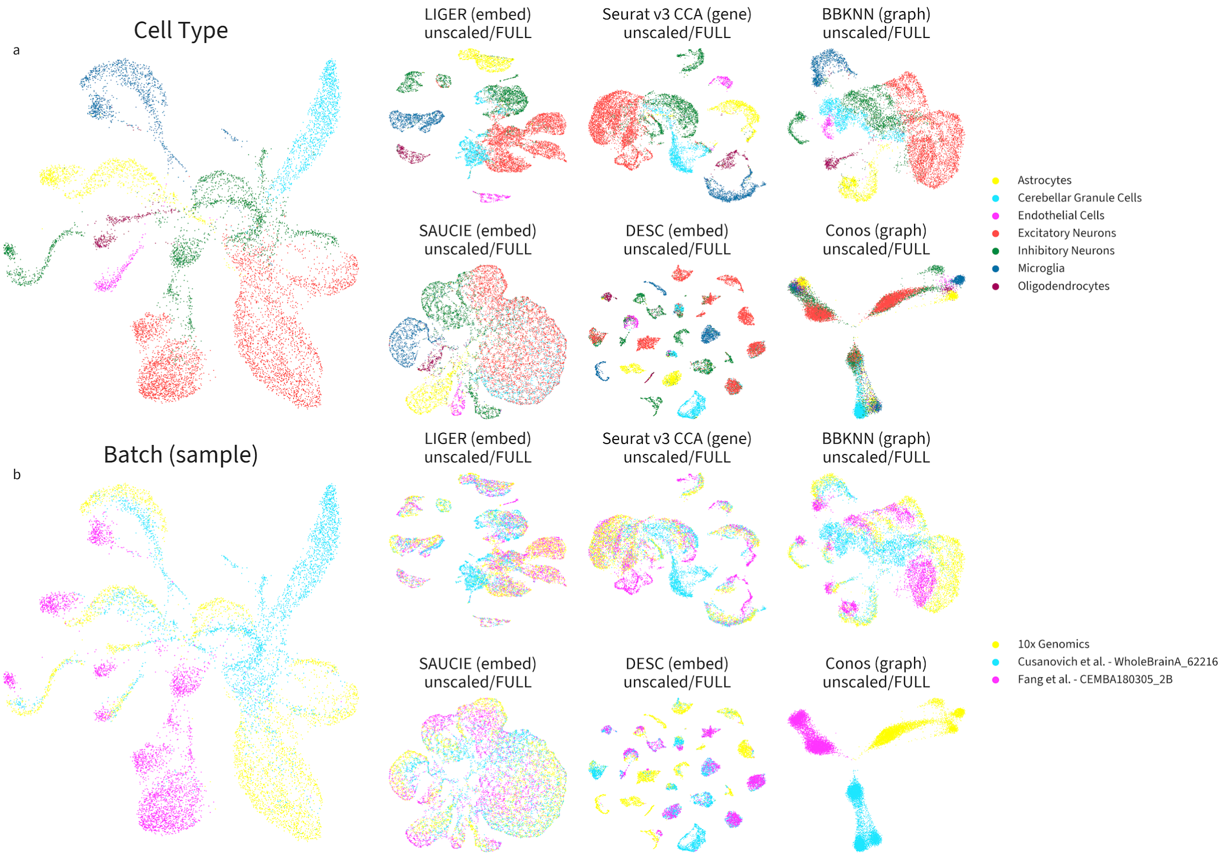

Supplementary Figure 25: Visualization of all small ATAC tasks using windows. The plots show Force Atlas 2 (Conos) and UMAP (all other methods) layouts for the unintegrated data (left), the top 3 performers (upper rows a and b), and the worst 3 performers (lower rows a and b). Plots are colored by (a) cell type annotations, and (b) batch labels.

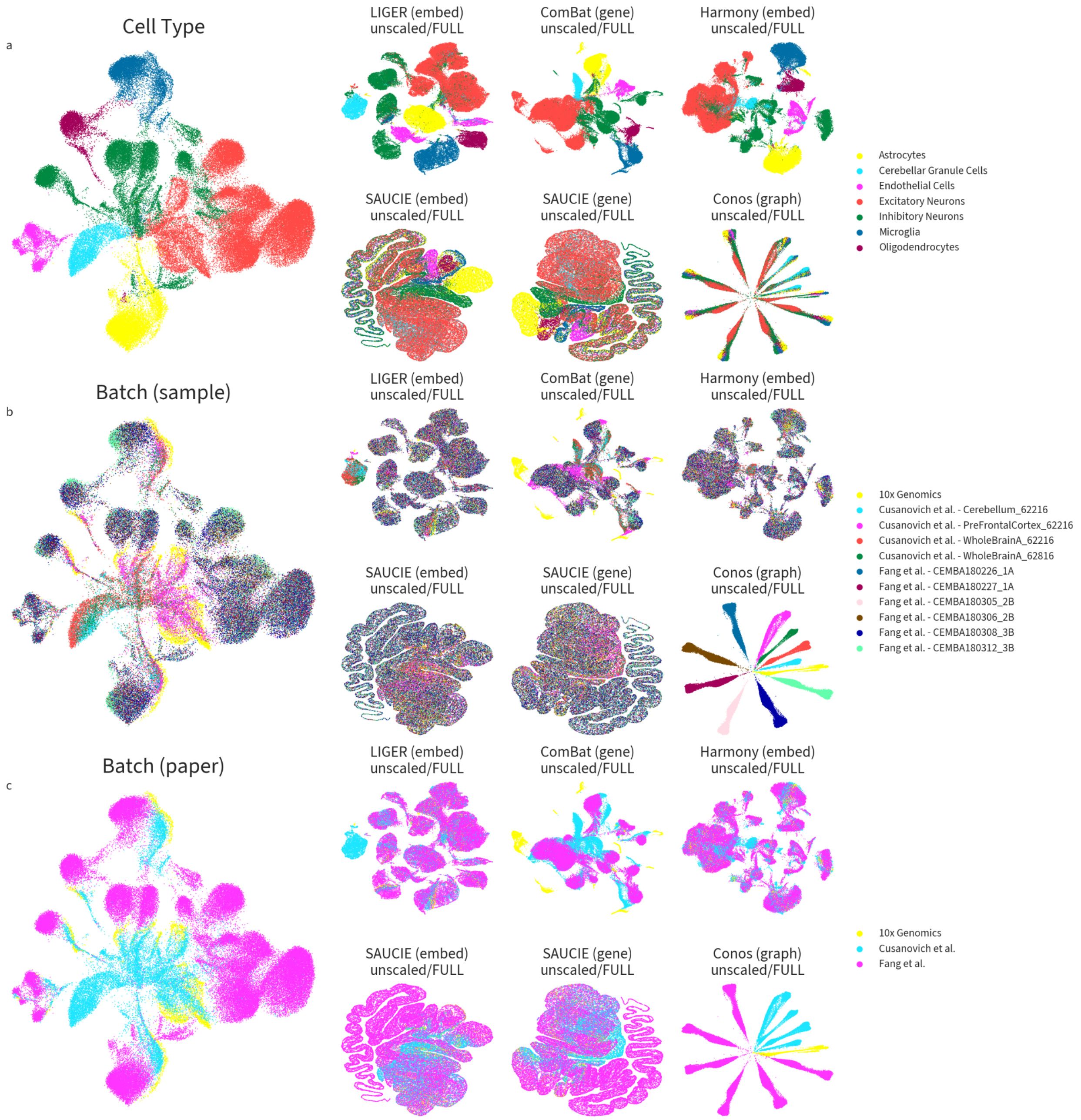

Supplementary Figure 26: Visualization of all large ATAC tasks using windows. The plots show Force Atlas 2 (Conos) and UMAP (all other methods) layouts for the unintegrated data (left), the top 3 performers (upper rows a and b), and the worst 3 performers (lower rows a and b). Plots are colored by (a) cell type annotations, (b) samples as batch labels, and (c) publications as batch labels.

Mouse brain (ATAC) - peaks

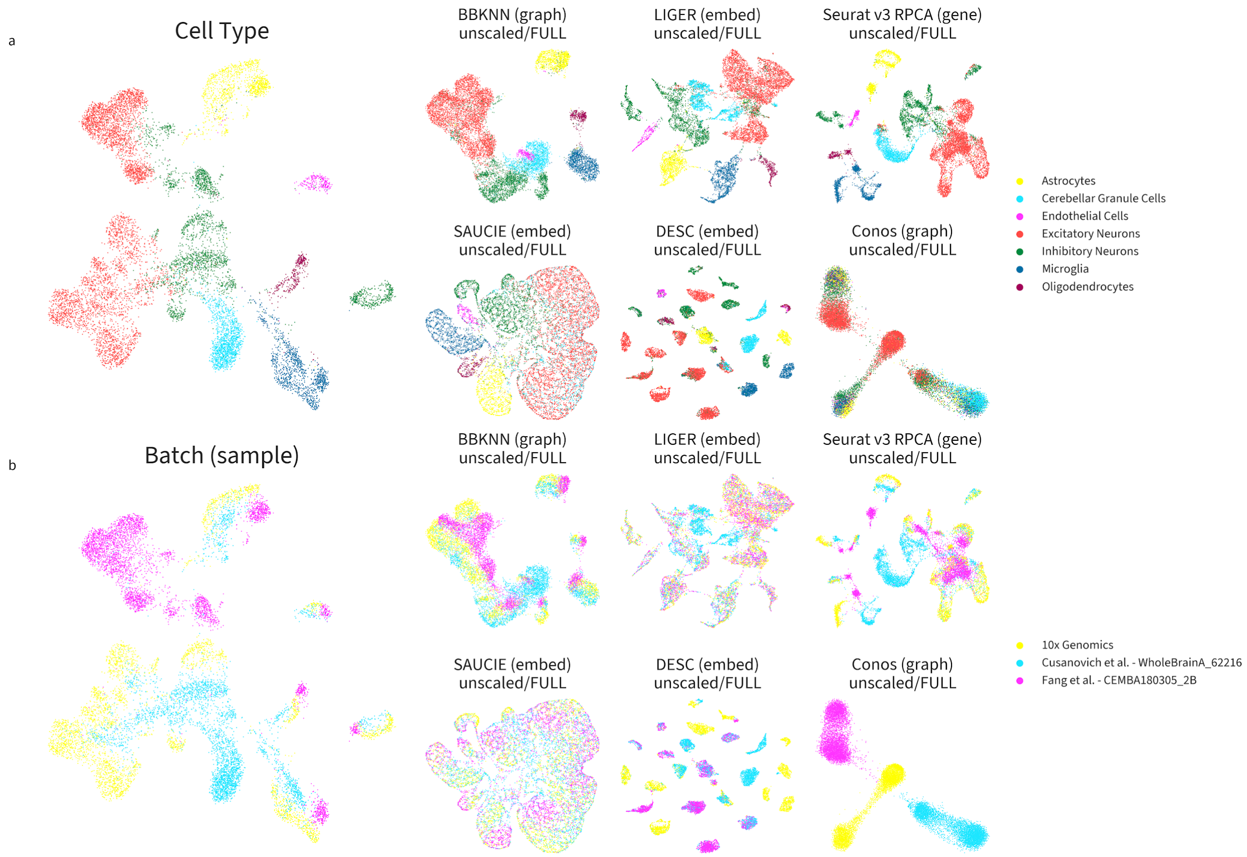

Supplementary Figure 27: Visualization of all small ATAC tasks using peaks. The plots show Force Atlas 2 (Conos) and UMAP (all other methods) layouts for the unintegrated data (left), the top 3 performers (upper rows a and b), and the worst 3 performers (lower rows a and b). Plots are colored by (a) cell type annotations, and (b) batch labels.

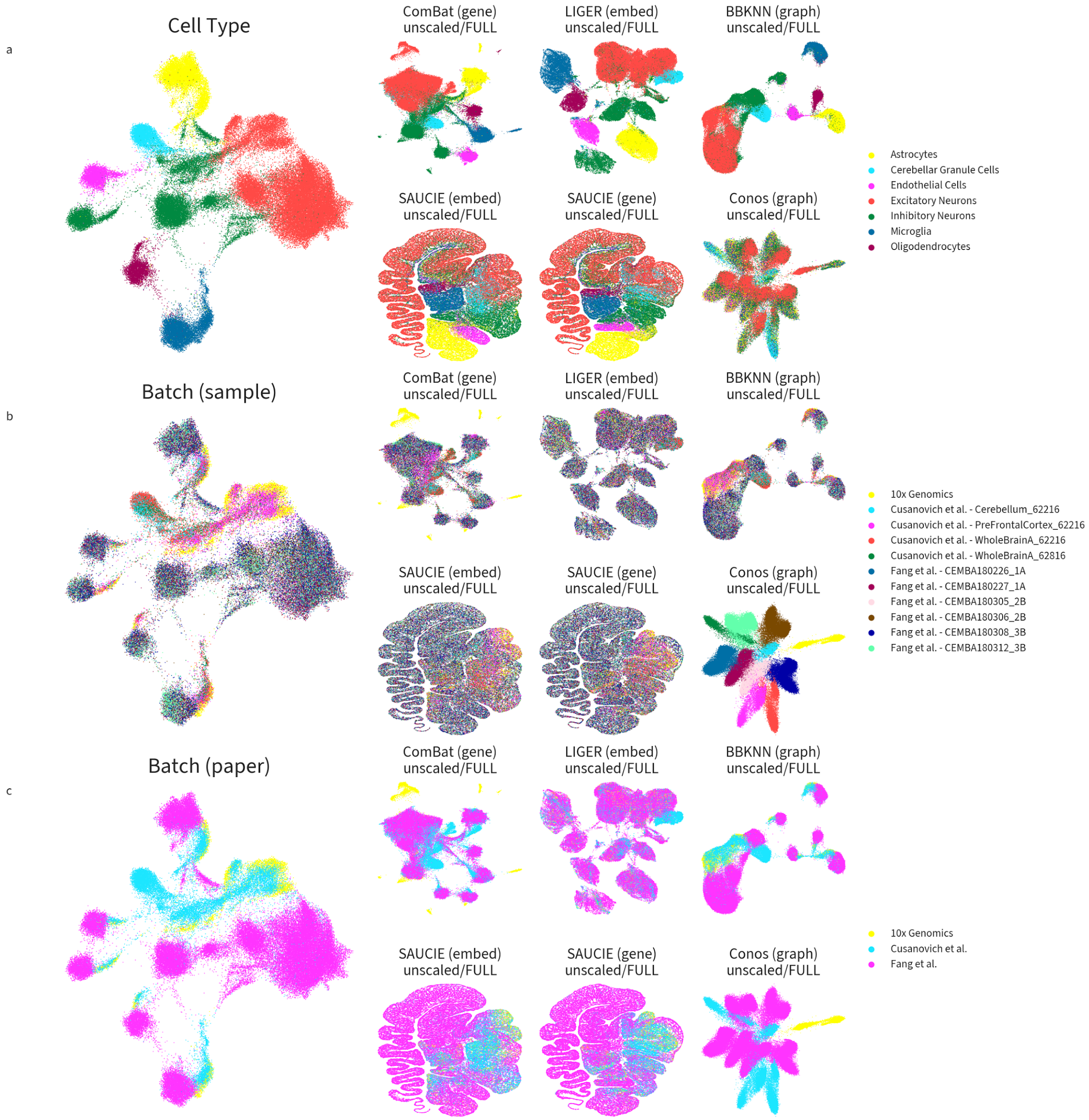

Supplementary Figure 28: Visualization of all large ATAC tasks using peaks. The plots show Force Atlas 2 (Conos) and UMAP (all other methods) layouts for the unintegrated data (left), the top 3 performers (upper rows a and b), and the worst 3 performers (lower rows a and

b). Plots are colored by (a) cell type annotations, (b) samples as batch labels, and (c) publications as batch labels.

Mouse brain (ATAC) - gene activity

a

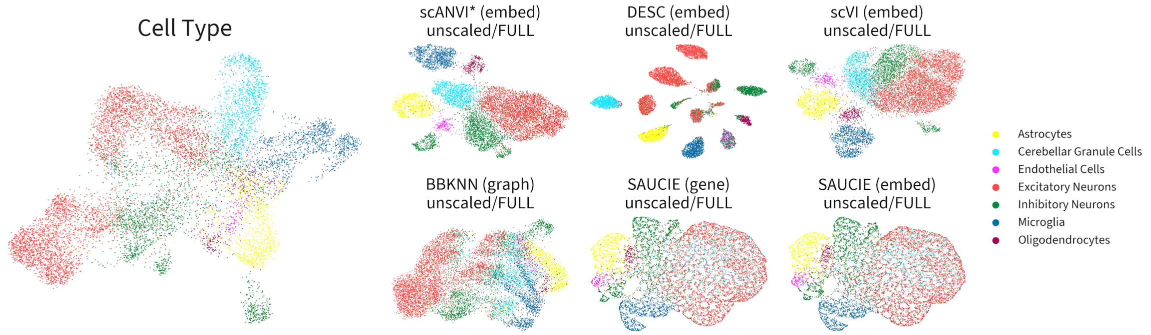

b

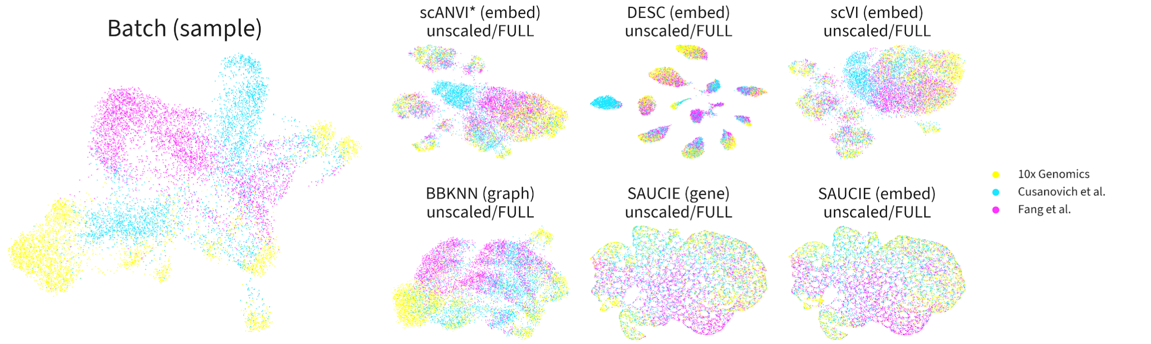

Supplementary Figure 29: Visualization of all small ATAC tasks using gene activity. The plots show UMAP layouts for the unintegrated data (left), the top 3 performers (upper rows a and b), and the worst 3 performers (lower rows a and b). Plots are colored by (a) cell type annotations, and (b) batch labels.

a

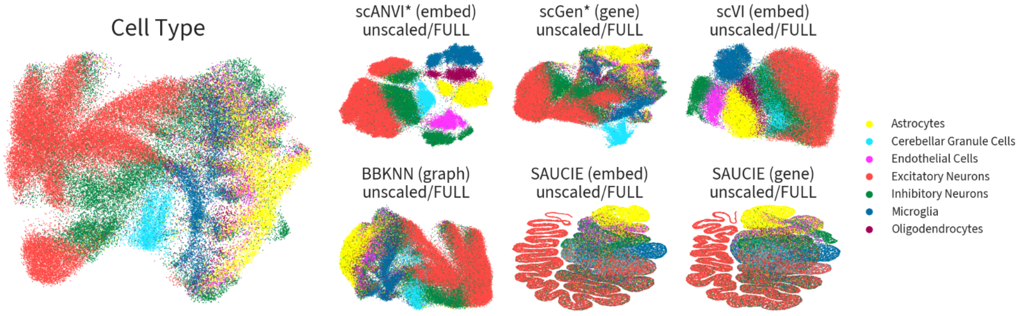

b

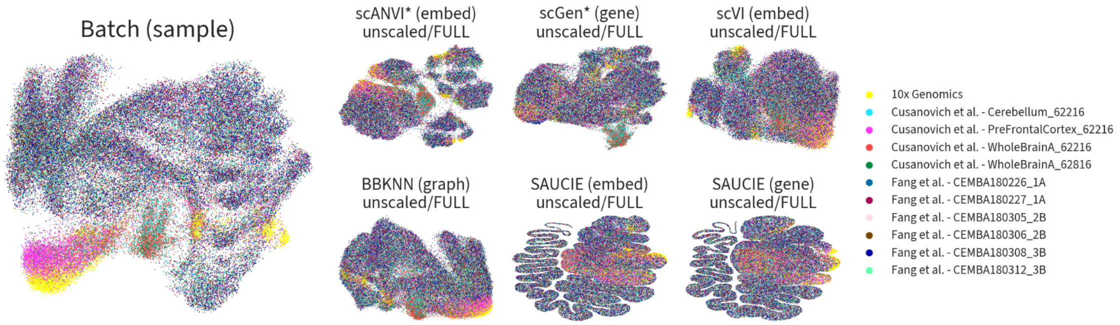

c

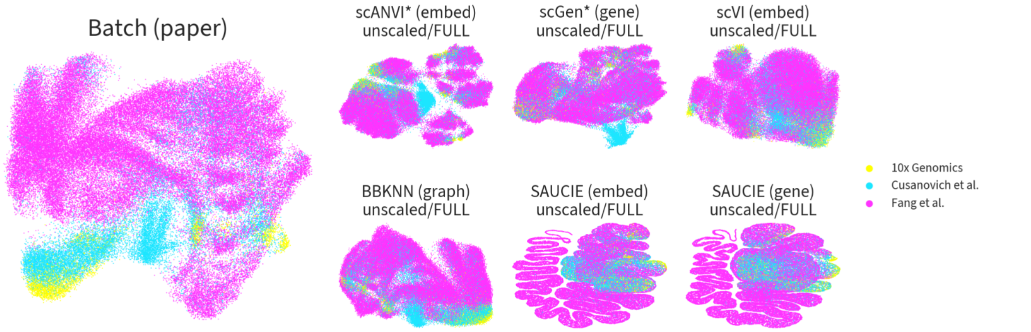

Supplementary Figure 30: Visualization of all large ATAC tasks using gene activity. The plots show UMAP layouts for the unintegrated data (left), the top 3 performers (upper rows a and b), and the worst 3 performers (lower rows a and b). Plots are colored by (a) cell type annotations, and (b) batch labels.

Metric rank distributions

Immune (human)

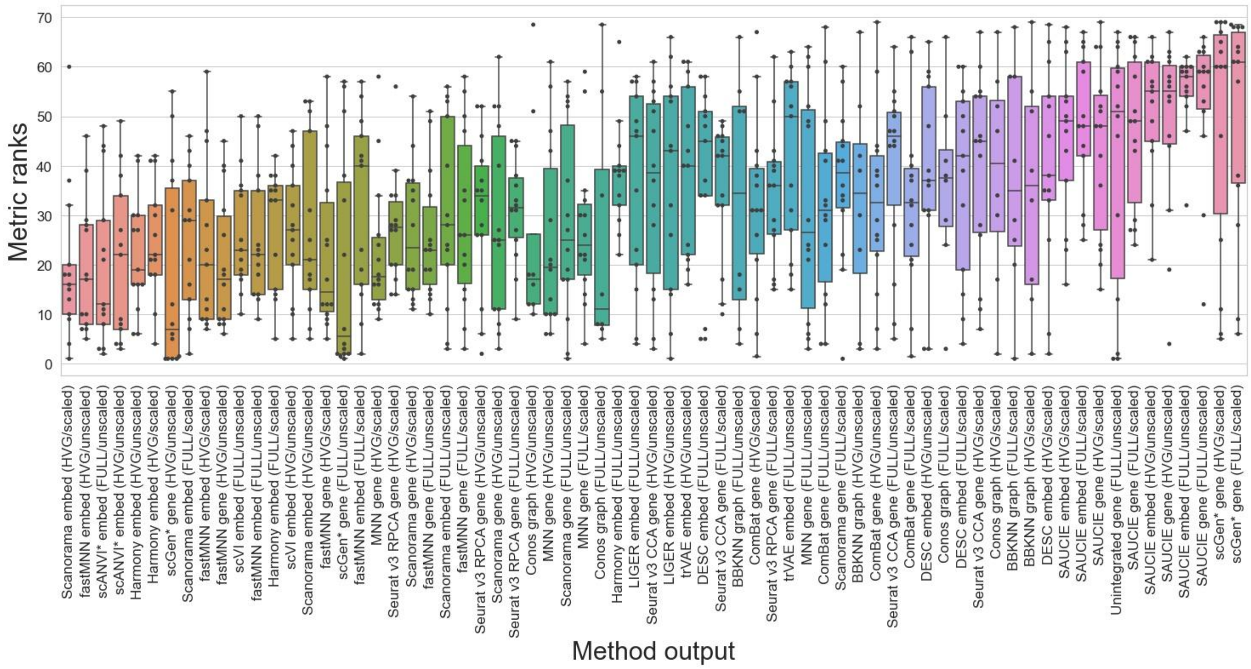

Supplementary Figure 31: Boxplot of metric rank distribution per integration output for the human immune cell task. All metric readouts were converted into ranks across integration outputs and plotted in a box-and-whisker plot. Box boundaries indicate 25% and 75% rank quantiles, and the center line indicates the median rank of all metrics run for a particular method output. Whiskers show the range of the metric rank distribution up to a value of 1.5 times the inter quartile range, after which metric ranks are shown as outlier data points. These statistics are computed over n=8, 13, and 14 independent metric outputs for “graph”, “embed”, and “gene” integration outputs respectively, as indicated by the method output label on the x-axis. All metric ranks per output are shown as data points overlaid onto the boxplot. Integration outputs are ordered by overall score.

Immune (human/mouse)

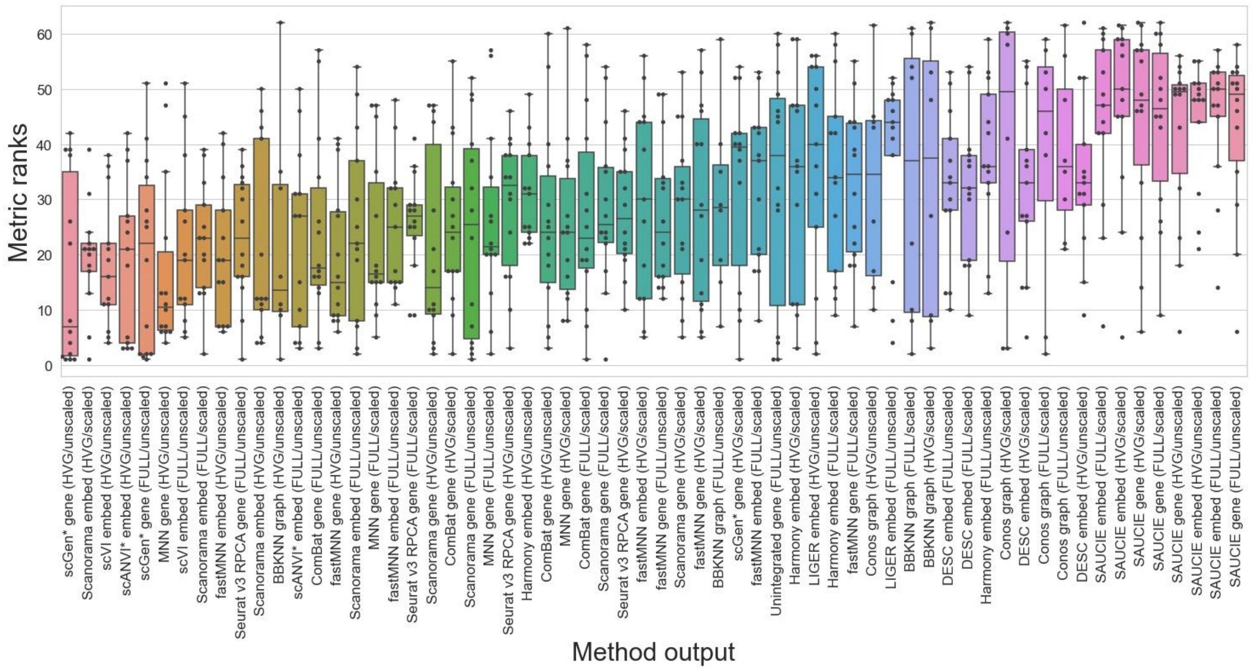

Supplementary Figure 32: Boxplot of metric rank distribution per integration output for the human mouse immune cell task. All metric readouts were converted into ranks across integration outputs and plotted in a box-and-whisker plot. Box boundaries indicate 25% and 75% rank quantiles, and the center line indicates the median rank of all metrics run for a particular method output. Whiskers show the range of the metric rank distribution up to a value of 1.5 times the inter quartile range, after which metric ranks are shown as outlier data points. These statistics are computed over n=8, 13, and 14 independent metric outputs for “graph”, “embed”, and “gene” integration outputs respectively, as indicated by the method output label on the x-axis. All metric ranks per output are shown as data points overlaid onto the boxplot. Integration outputs are ordered by overall score.

Simulation 1

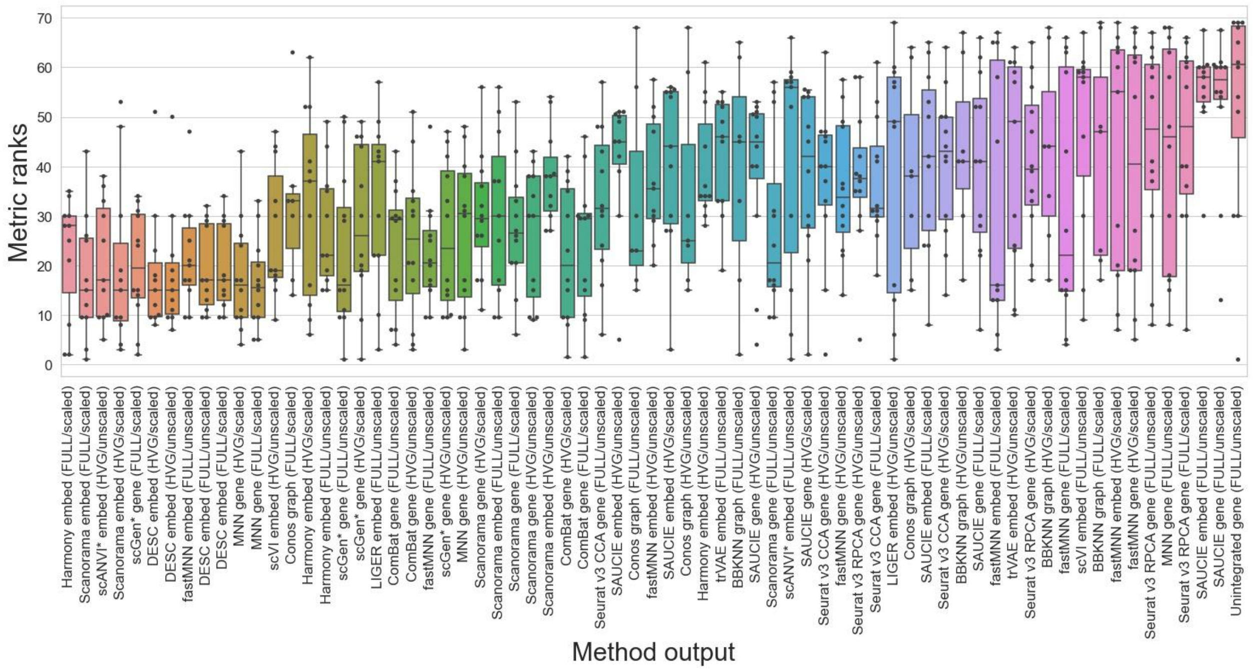

Supplementary Figure 33: Boxplot of metric rank distribution per integration output for the simulation 1 task. All metric readouts were converted into ranks across integration outputs and plotted in a box-and-whisker plot. Box boundaries indicate 25% and 75% rank quantiles, and the center line indicates the median rank of all metrics run for a particular method output. Whiskers show the range of the metric rank distribution up to a value of 1.5 times the inter quartile range, after which metric ranks are shown as outlier data points. These statistics are computed over n=7, 11, and 12 independent metric outputs for “graph”, “embed”, and “gene” integration outputs respectively, as indicated by the method output label on the x-axis. All metric ranks per output are shown as data points overlaid onto the boxplot. Integration outputs are ordered by overall score.

Simulation 2

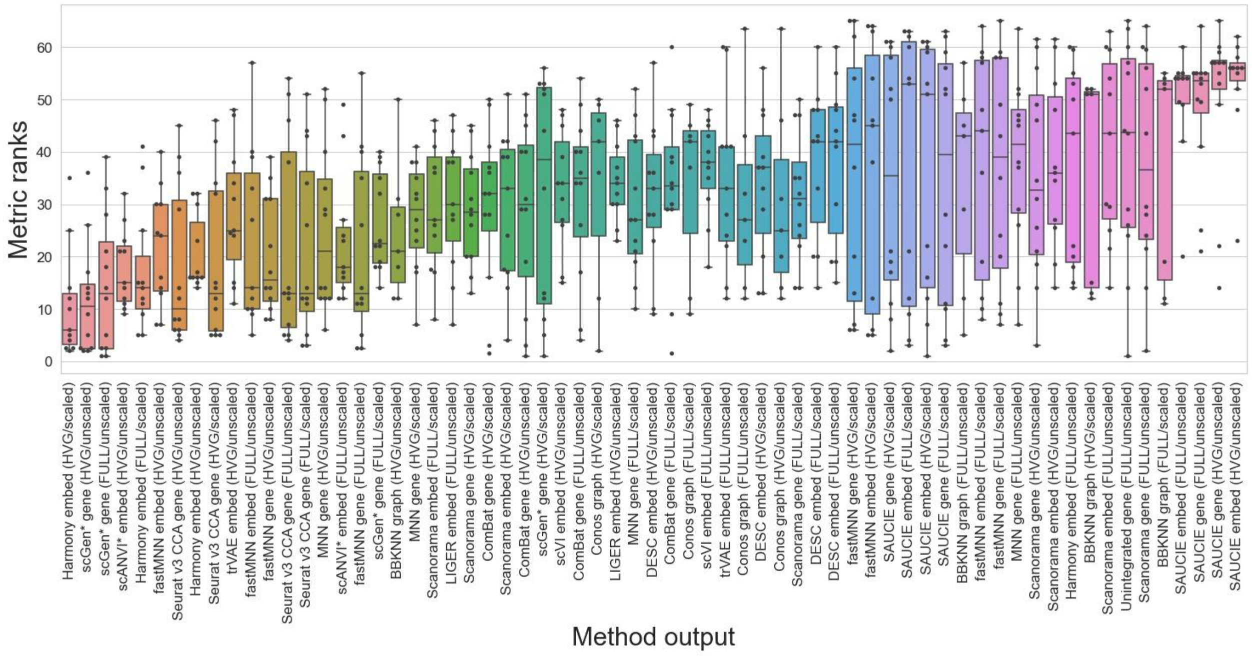

Supplementary Figure 34: Boxplot of metric rank distribution per integration output for the simulation 2 task. All metric readouts were converted into ranks across integration outputs and plotted in a box-and-whisker plot. Box boundaries indicate 25% and 75% rank quantiles, and the center line indicates the median rank of all metrics run for a particular method output. Whiskers show the range of the metric rank distribution up to a value of 1.5 times the inter quartile range, after which metric ranks are shown as outlier data points. These statistics are computed over n=7, 11, and 12 independent metric outputs for “graph”, “embed”, and “gene” integration outputs respectively, as indicated by the method output label on the x-axis. All metric ranks per output are shown as data points overlaid onto the boxplot. Integration outputs are ordered by overall score.

Pancreas

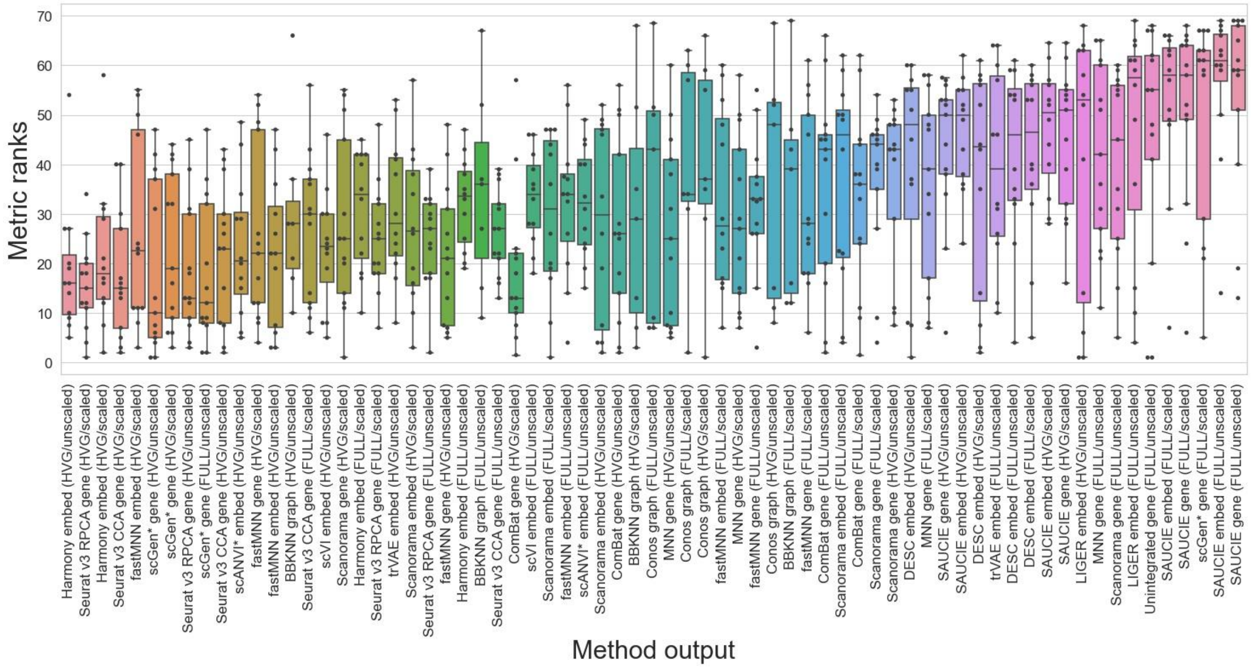

Supplementary Figure 35: Boxplot of metric rank distribution per integration output for the pancreas integration task. All metric readouts were converted into ranks across integration outputs and plotted in a box-and-whisker plot. Box boundaries indicate 25% and 75% rank quantiles, and the center line indicates the median rank of all metrics run for a particular method output. Whiskers show the range of the metric rank distribution up to a value of 1.5 times the inter quartile range, after which metric ranks are shown as outlier data points. These statistics are computed over n=7, 12, and 13 independent metric outputs for “graph”, “embed”, and “gene” integration outputs respectively, as indicated by the method output label on the x-axis. All metric ranks per output are shown as data points overlaid onto the boxplot. Integration outputs are ordered by overall score.

Lung

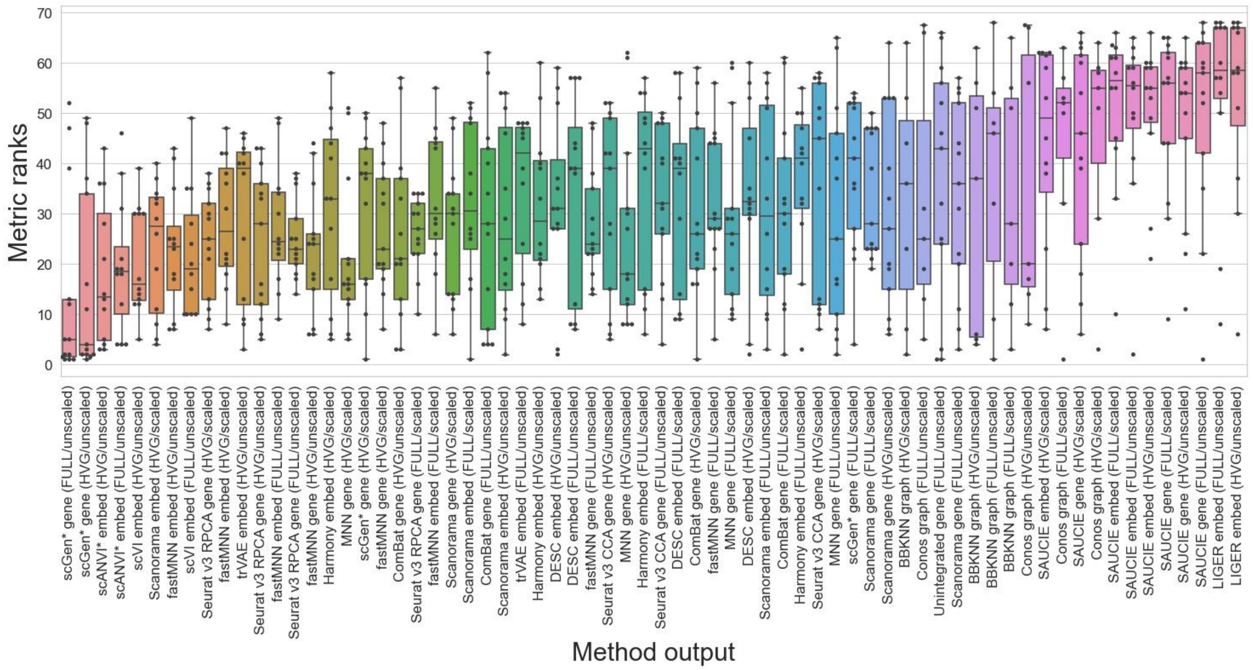

Supplementary Figure 36: Boxplot of metric rank distribution per integration output for the lung atlas integration task. All metric readouts were converted into ranks across integration outputs and plotted in a box-and-whisker plot. Box boundaries indicate 25% and 75% rank quantiles, and the center line indicates the median rank of all metrics run for a particular method output. Whiskers show the range of the metric rank distribution up to a value of 1.5 times the inter quartile range, after which metric ranks are shown as outlier data points. These statistics are computed over n=7, 12, and 13 independent metric outputs for “graph”, “embed”, and “gene” integration outputs respectively, as indicated by the method output label on the x-axis. All metric ranks per output are shown as data points overlaid onto the boxplot. Integration outputs are ordered by overall score.

Mouse brain

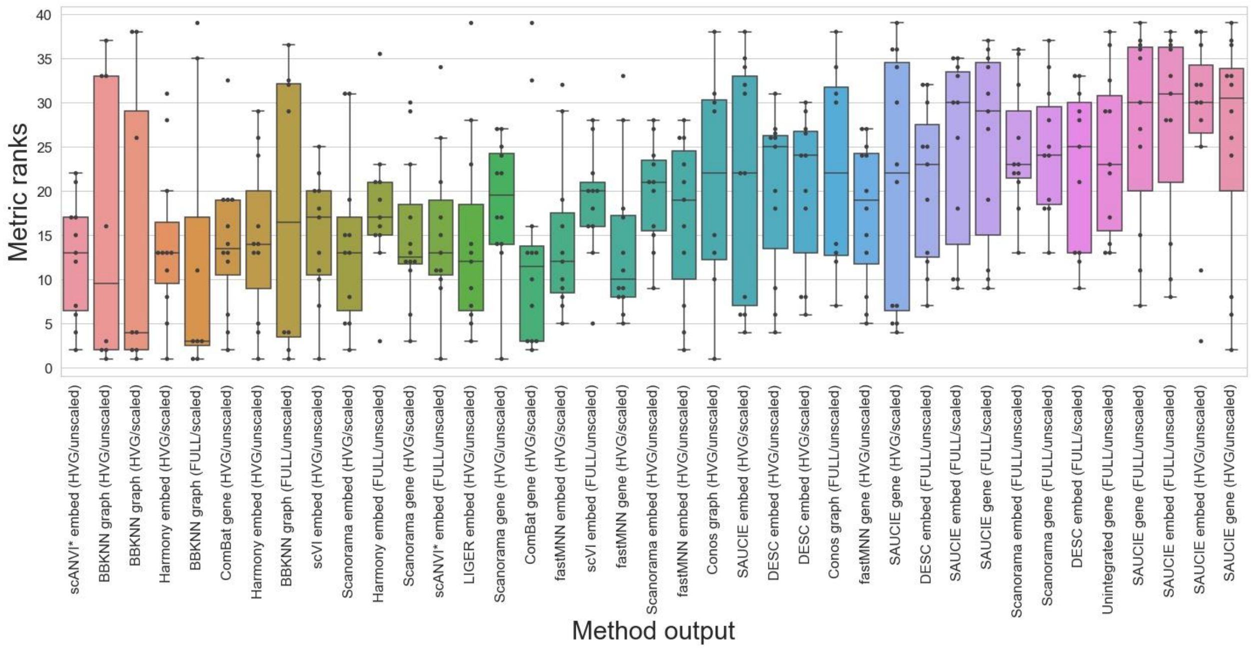

Supplementary Figure 37: Boxplot of metric rank distribution per integration output for the mouse brain RNA integration task. All metric readouts were converted into ranks across integration outputs and plotted in a box-and-whisker plot. Box boundaries indicate 25% and 75% rank quantiles, and the center line indicates the median rank of all metrics run for a particular method output. Whiskers show the range of the metric rank distribution up to a value of 1.5 times the inter quartile range, after which metric ranks are shown as outlier data points. These statistics are computed over n=7, 12, and 13 independent metric outputs for “graph”, “embed”, and “gene” integration outputs respectively, as indicated by the method output label on the x-axis. All metric ranks per output are shown as data points overlaid onto the boxplot. Integration outputs are ordered by overall score.

Mouse brain (ATAC) - all feature spaces

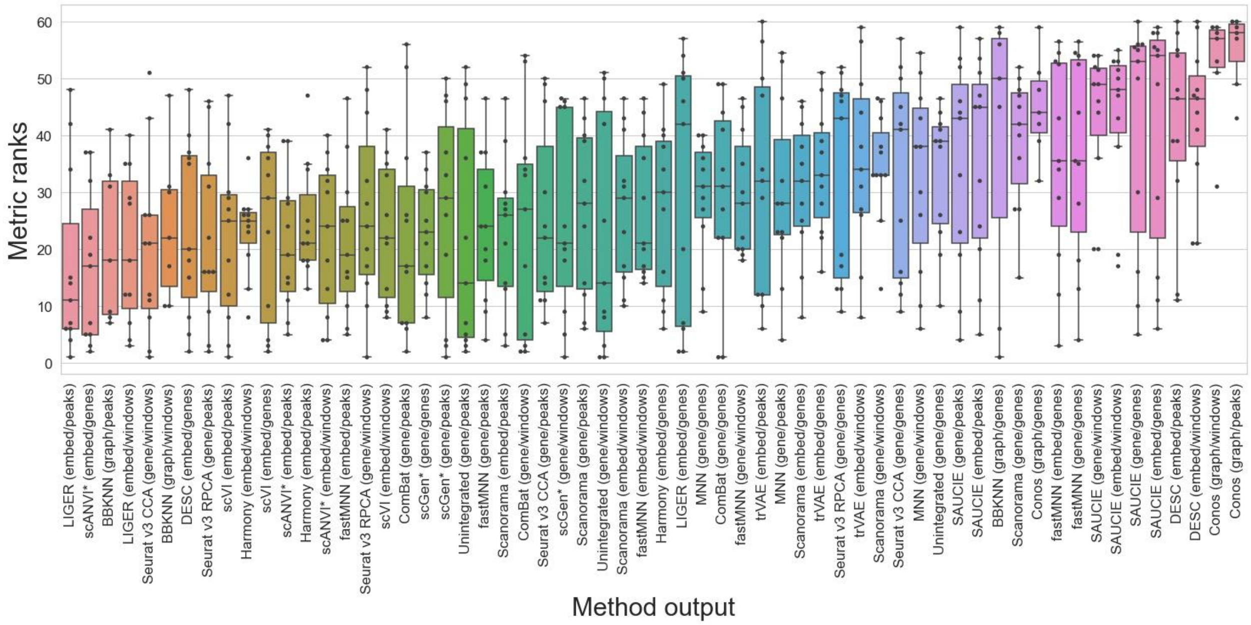

Supplementary Figure 38: Boxplot of metric rank distribution per integration output for all small mouse brain tasks for all feature spaces based on scATAC-seq. All metric readouts were converted into ranks across integration outputs and plotted in a box-and-whisker plot. Box boundaries indicate 25% and 75% rank quantiles, and the center line indicates the median rank of all metrics run for a particular method output. Whiskers show the range of the metric rank distribution up to a value of 1.5 times the inter quartile range, after which metric ranks are shown as outlier data points. These statistics are computed over n=7, 11, and 11 independent metric outputs for “graph”, “embed”, and “gene” integration outputs respectively, as indicated by the method output label on the x-axis. All metric ranks per output are shown as data points overlaid onto the boxplot. Integration outputs are ordered by overall score.

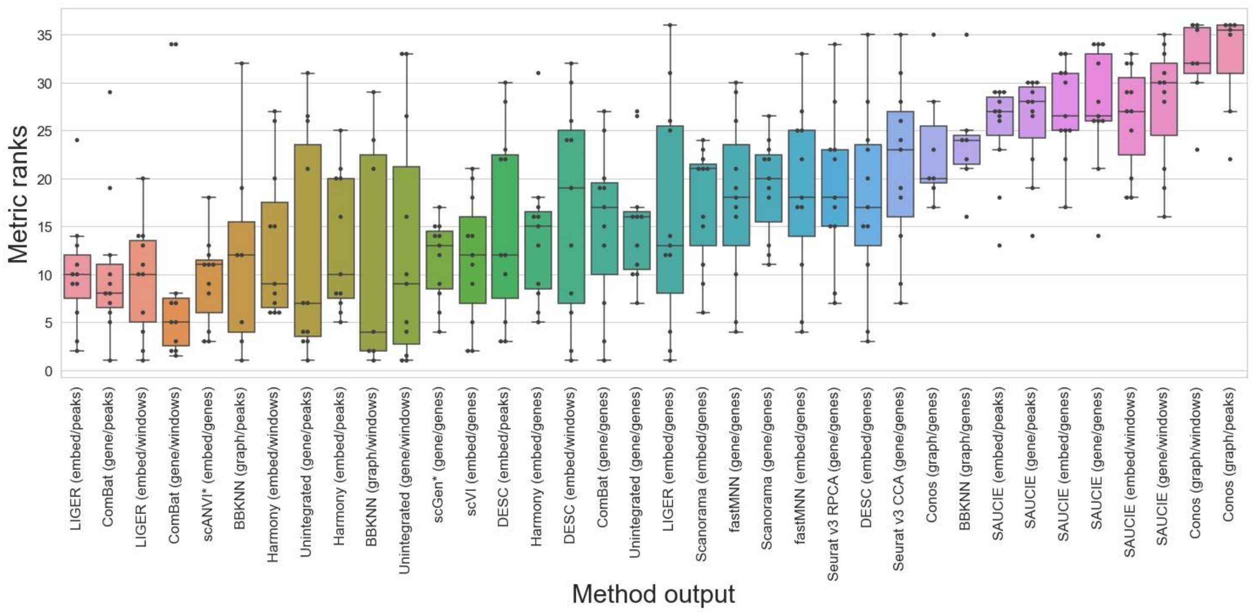

Supplementary Figure 39: Boxplot of metric rank distribution per integration output for all large mouse brain tasks for all feature spaces based on scATAC-seq. All metric readouts were converted into ranks across integration outputs and plotted in a box-and-whisker plot. Box boundaries indicate 25% and 75% rank quantiles, and the center line indicates the median rank of all metrics run for a particular method output. Whiskers show the range of the metric rank distribution up to a value of 1.5 times the inter quartile range, after which metric ranks are shown as outlier data points. These statistics are computed over n=7, 11, and 11 independent metric outputs for “graph”, “embed”, and “gene” integration outputs respectively, as indicated by the method output label on the x-axis. All metric ranks per output are shown as data points overlaid onto the boxplot. Integration outputs are ordered by overall score.

Parameter optimization - Pancreas task

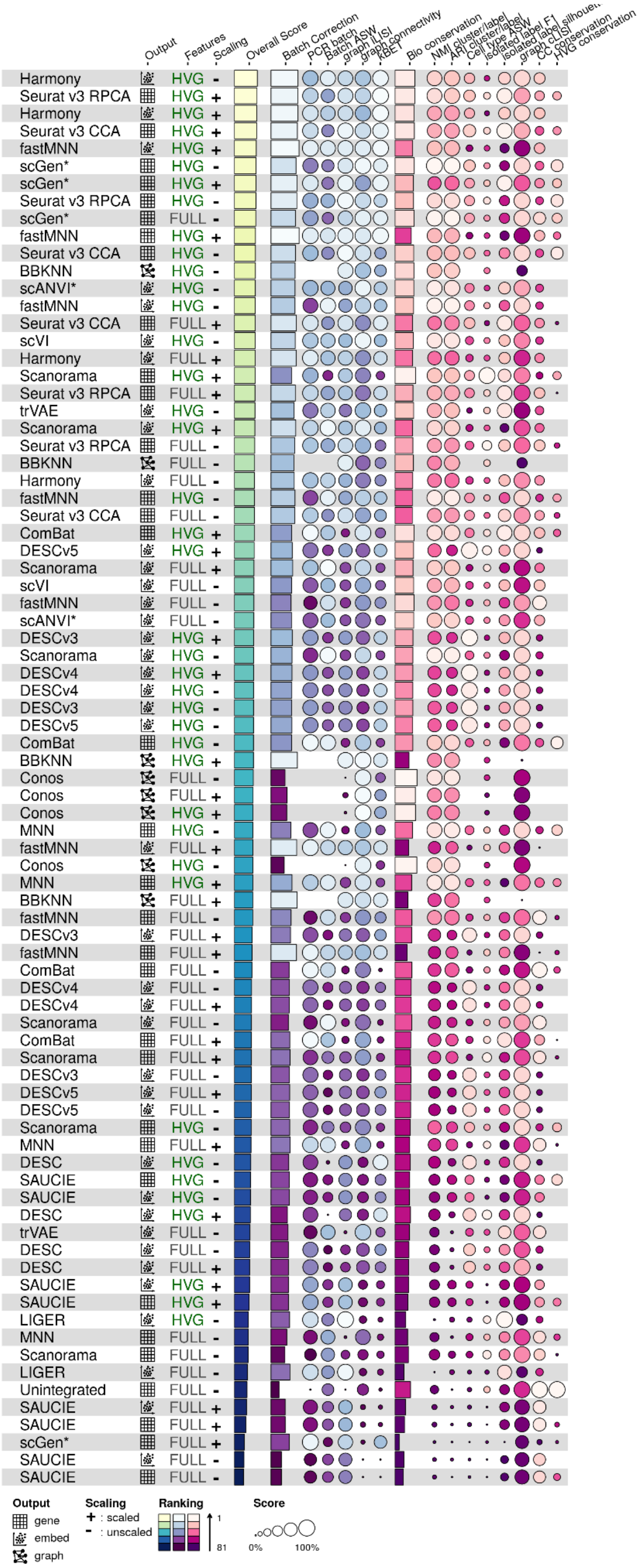

Supplementary Figure 40: Overview of benchmarking results by overall score for the pancreas task with parameter optimized DESC outputs. Metrics are divided into batch correction (blue, purple) and bio conservation (pink) categories. Overall scores are computed by a 40:60 weighted mean of these category scores (see **Methods** for further visualization details). DESCv3, DESCv4, DESCv5 have resolution parameters of 0.3, 0.4, and 0.5 respectively, compared to the default parameter of 0.8 that was used for the default DESC run. With an optimized parameterization, DESCv5 improves to rank 34 compared to rank 51 for non-optimized DESC.

iLISI comparison

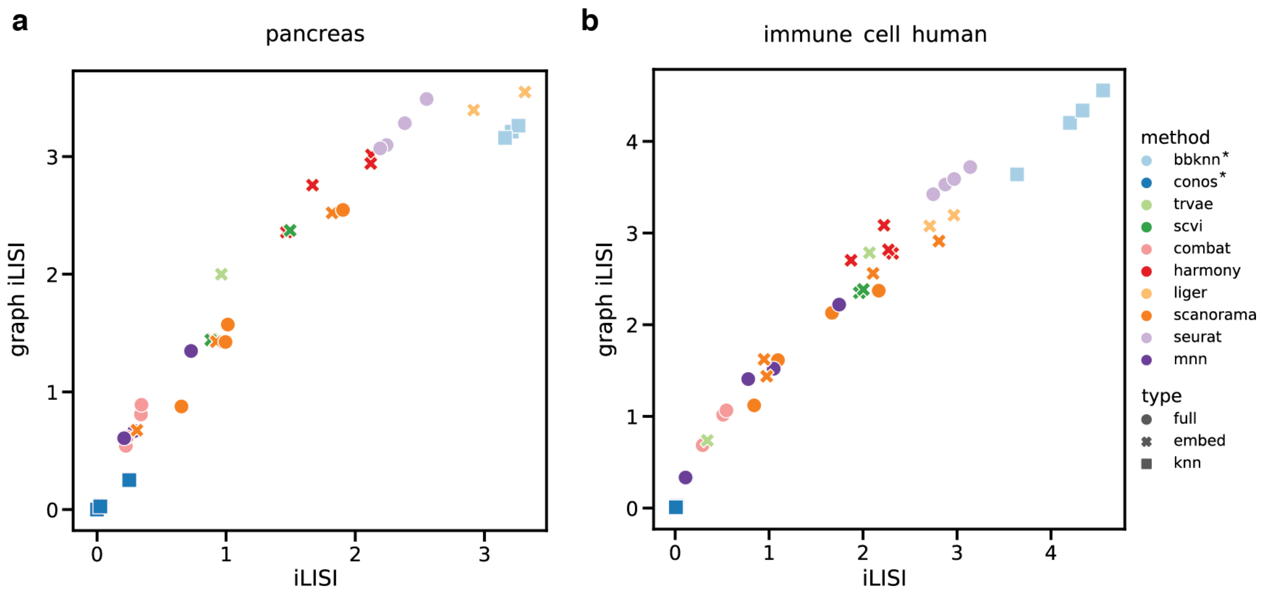

Supplementary Figure 41: Comparison of graph iLISI and iLISI scores. All scores are unscaled, but shifted by -1 for visualization. For graph-based output (marked with an asterisk in the legend and a filled square in the plot), iLISI could not be computed, because these methods do not provide an output on which Euclidean distance can be measured. As iLISI cannot be computed on graph output, the displayed results for graph-based methods were computed with graph iLISI on both x- and y-axis and thus the points lie on the diagonal. We compared both implementations in the pancreas (a) and immune cell human (b) data scenarios. Both scores correlate well on full- and embedding-based data integration methods (circles and crosses), *i.e.* Pearson correlation coefficient is 0.978 for the pancreas task and 0.984 for the immune cell human task.

Trajectories - addendum

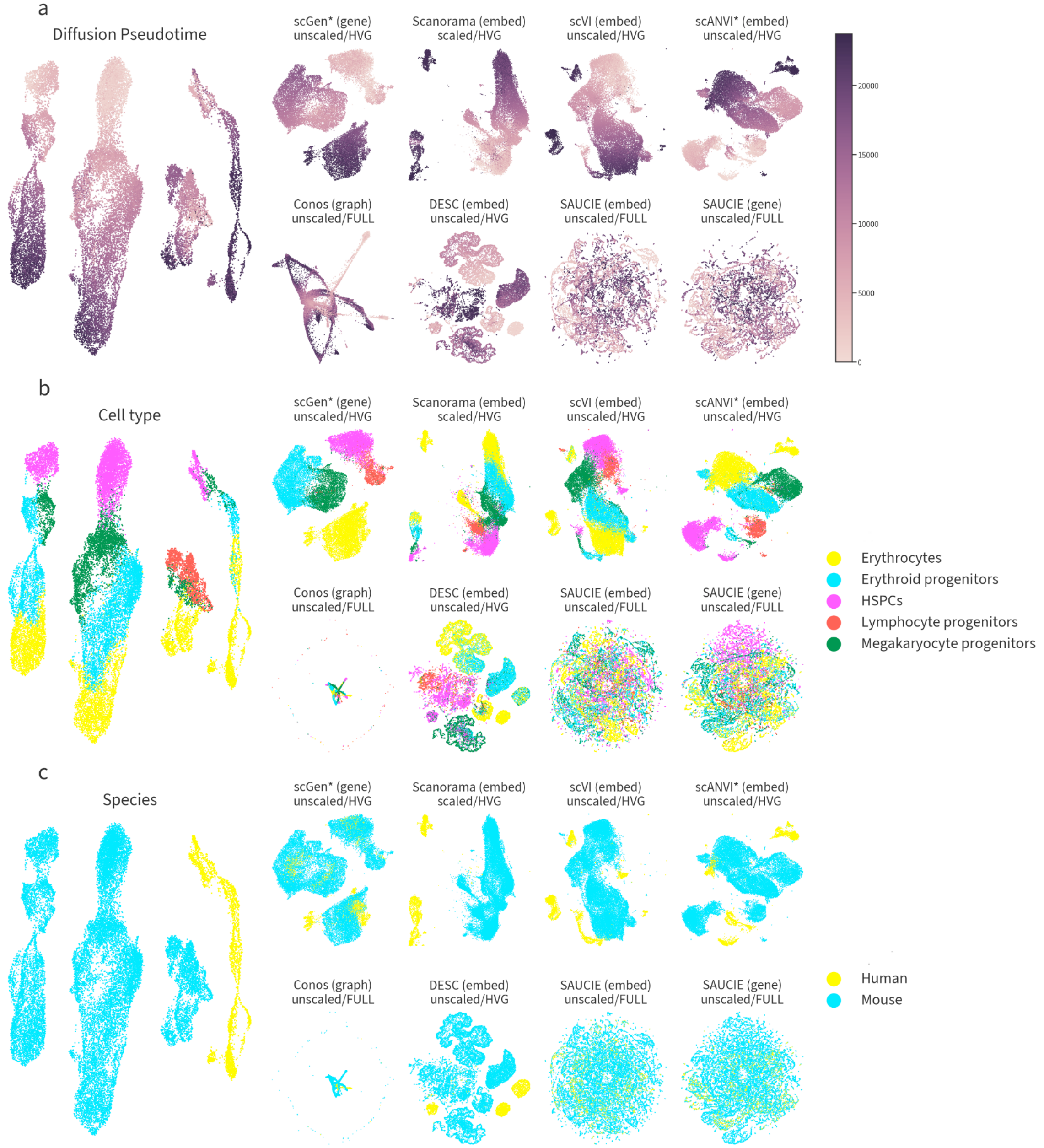

Supplementary Figure 42: Visualization of the best and worst performers on the immune cell human mouse integration task ordered by overall score. The plots show Force Atlas 2 (Conos) and UMAP (all other methods) layouts for the unintegrated data (left), the top 4 performers (upper rows a, b and c), and the worst 4 performers (lower rows a, b and c). Plots are colored by (a) diffusion pseudotime, (b) cell identity annotations, and (c) species. All embeddings only show the cells for which a pseudotime could be computed (typically the largest connected component of the kNN graph). If the trajectory can't be computed, the embedding remains empty, as is the case for SAUCIE (gene) unscaled/full.

Supplementary Table 1: Data integration methods

Supplementary Table 1: **Data integration methods available in order of first preprint publication.** Methods that perform only time-series data integration are omitted. The collection is based on manual literature review and scrna-tools.org[50]; (last updated: November 2020)

Method	Method principle	DOI/arXiv ID/url	GitHub	First preprint Date
MNN	Mutual nearest neighbors	10.1038/nbt.4091	chriscainx/mnnpy<br><br>LTLA/batchelor	July 18, 2017
Seurat v2	Canonical correlation analysis (CCA)	10.1038/nbt.4096	satijalab/seurat	July 18, 2017
SAUCIE	Sparse, regularized autoencoder	10.1038/s41592-019-0576-7	KrishnaswamyLab/SAUCIE	December 19, 2017
MAGAN	Manifold alignment generative adversarial network	http://proceedings.mlr.press/v80/amodio18a.html	KrishnaswamyLab/MAGAN	February 10, 2018
scVI	Conditional variational autoencoder	10.1038/s41592-018-0229-2	YosefLab/scvi-tools	March 30, 2018
Scanorama	SVD + Mutual nearest neighbors	10.1038/s41587-019-0113-3	brianhie/scanorama	July 17, 2018
BBKNN	KNN graph integration	10.1093/bioinformatics/btz625	https://github.com/Teichlab/bbknn	August 22, 2018
scMerge	Factor analysis model on stably expressed genes	10.1073/pnas.1820006116	SydneyBioX/scMerge	September 12, 2018
Conos	PCA + KNN integration	10.1038/s41592-019-0466-z	kharchenkolab/conos	November 2, 2018

LIGER	Integrative non-negative matrix factorization	10.1016/j.cell.2019.05.006	MacoskoLab/liger	November 2, 2018
Seurat v3	CCA + Mutual nearest neighbors	10.1016/j.cell.2019.05.031	satijalab/seurat	November 02, 2018
Harmony	PCA + clustering-based correction	10.1038/s41592-019-0619-0	immunogenomics/harmony	November 04, 2018
scGen	Conditional variational autoencoder (cell identity labels required)	10.1038/s41592-019-0494-8	theislab/scgen	November 29, 2018
RISC	Principal component regression	10.1101/483297	N/A	November 29, 2018
scAlign	Bidirectional mapping through deep learning	10.1186/s13059-019-1766-4	quon-titative-biology/scAlign	December 22, 2018
scPopCorn	Simultaneous optimisation of subpopulations across samples	10.1016/j.cels.2019.05.007	ncbi/scPopCorn	December 28, 2018
DESC	Autoencoder with iterative clustering	10.1038/s41467-020-15851-3	eleozzr/desc	January 25, 2019
scANVI	Semi-supervised variational inference with deep generative models	10.1101/532895	YosefLab/scvi-tools	January 29, 2019
BUSseq	Fits a Bayesian hierarchical model	10.1101/533372	songfd2018/BUSseq-0.99.0	January 29, 2019

FastMNN	PCA + Mutual nearest neighbors	https://marionilab.github.io/FurtherMNN2018/theory/description.html	LTLA/batchelor	June 3, 2019
scBatch	Sample distance matrix adjustment	10.1093/bioinformatics/btaa097	tengfei-emory/scBatch	June 13, 2019
Bermuda	Autoencoder with transfer learning	10.1186/s13059-019-1764-6	txWang/BERMUDA	July 2, 2019
SMNN	Supervised mutual nearest neighbors	10.1093/bib/bbaa097	yycunc/SMNN	September 20, 2019
BEER	Removal of PCs with batch effects	10.1038/s41421-019-0114-x	jumphone/BEER	September 24, 2019
trVAE	Conditional variational autoencoder	arXiv:1910.01791	theislab/trvae	October 4, 2019
Pegasus	Classical location and scale adjustment	10.1038/s41592-020-0905-x	klarman-cell-observatory/pegasus	October 30, 2019
MOFA2	Multi factor analysis model	10.1101/837104	bioFAM/MOFA2	November 9, 2019
scadKNN	Autoencoder (+ KNN classification)	10.1109/BIBM47256.2019.8982969	N/A	November 18, 2019
scPhere	Variational autoencoder	10.1101/853457	klarman-cell-observatory/scPhere	November 25, 2019
Dmatch	Kernel density matching with external reference	10.1101/2020.01.05.895136	qzhan321/Dmatch	January 6, 2020
scDGN	Adversarial networks (cell identity labels required)	10.1101/2020.01.06.896621	SongweiGe/scDGN	January 7, 2020

sstGPLVM	Gaussian process latent variable model with t-distributed noise	10.1101/2020.01.14.906313	architverma1/sc-manifold-alignment	January 14, 2020
BATMAN	Minimum weight matching on bipartite graph	10.1016/j.isci.2020.101185	mandricigor/batman	January 23, 2020
MARS	Learns a joint embedding with landmarks	10.1101/2020.02.25.960302	snap-stanford/mars	February 26, 2020
CSS	Represent cells by similarity to clusters in individual samples	10.1186/s13059-020-02147-4	quadbiolab/simspec	February 28, 2020
SCIPR	Iterative point set registration	10.1101/2020.05.13.093948	AmirAlavi/scipr	May 13, 2020
INSCT	Batch-aware triplet neural network	10.1101/2020.05.16.100024	lkmklsmn/insct	May 17, 2020
FIRM	Rescaling based on subsampling of clusters	10.1101/2020.06.02.129031	mingjingsi/FIRM	June 3, 2020
Monet	Mutual nearest neighbors in PCA space	10.1101/2020.06.08.140673	flo-compbio/monet	June 9, 2020
BC_tSNE	Batch-aware projected t-SNE	10.1093/bioinformatics/btaa189	emanuelealiverti/BC_tSNE	June 11, 2020
SCIM	Technology-invariant autoencoder and bipartite matching	10.1101/2020.06.11.146845	ratschlab/scim	June 14, 2020
STACAS	Alternative anchor identification for the Seurat method	10.1101/2020.06.15.152306	carmonalab/STACAS	June 15, 2020

cFIT	High-dimensional linear model with latent low-dimensions	10.1101/2020.08.31.276345	pengminshi/cFIT	August 31, 2020
scIntegral	Semi-supervised linear model using marker genes	10.1101/2020.09.17.301911	hanbin973/scIntegral	September 19, 2020
CarDEC	Self-supervised embedding and clustering network	10.1101/2020.09.23.310003	jlakkis/CarDEC	September 25, 2020
Dincta	Iterative clustering and embedding adjustment	10.1101/2020.09.28.316901	songtingstone/dincta	September 30, 2020
JIND	Encoder and classifier network	10.1101/2020.10.06.327601	mohit1997/JIND	October 7, 2020
iSMNN	Iterative supervised mutual nearest neighbors	10.1101/2020.11.09.375659	yycunc/iSMNN	November 10, 2020
corral	Multi-table correspondence analysis		laurenhsu1/corral	

Supplementary Table 2: Metrics runs

Supplementary Table 2: Applicability of metrics to data integration outputs. Specifically metrics for beyond-label conservation cannot be run on all outputs such as corrected graph outputs and ATAC tasks. The asterisk (*) denotes that no relevant trajectories were found in the ATAC tasks and none were input into the simulation tasks.

**Metric**	**Graph**	**Embedding**	**Feature**	**RNA**	**ATAC**	**Simulation**
PCR batch		✘	✘	✘	✘	✘
Batch ASW		✘	✘	✘	✘	✘
Graph connectivity	✘	✘	✘	✘	✘	✘
Graph iLISI	✘	✘	✘	✘	✘	✘
kBET	✘	✘	✘	✘	✘	✘
Normalized Mutual Information	✘	✘	✘	✘	✘	✘
Average Rand Index	✘	✘	✘	✘	✘	✘
Cell type ASW		✘	✘	✘	✘	✘
Graph cLISI	✘	✘	✘	✘	✘	✘
Isolated label F1	✘	✘	✘	✘	✘	✘
Isolated label ASW		✘	✘	✘	✘	✘
Cell cycle conservation		✘	✘	✘		
HVG conservation			✘	✘		✘
Trajectory conservation	✘	✘	✘	✘	*	*

Supplementary Note 1: Extending kBET for fair assessment of graph-based integration results

Evaluating how well batch effects are removed in an integration task is complicated by different output formats. Any evaluation metric that can compare graph-based outputs and joint embeddings or corrected feature matrices, must work on the integrated graph (a connectivity matrix). For joint embeddings or corrected feature matrices, such a graph is computed by finding k nearest neighbors based on pairwise distances between cells in the embedding. This process results in a graph where each node has the same out-degree (edges leading outwards). In contrast, a graph-based integration method can output an integration graph with varying *k* per neighborhood. This neighborhood size variance is particularly noticeable in the outputs generated by Conos[44].

We use the kBET[51] metric to assess batch removal for kNN-based outputs. Here, the choice of *k* determines the statistical power of the test per neighborhood. Thus, having a variable *k* means that the rejection of the null hypothesis is less likely in certain regions. As a result, it is important to have a consistent minimal *k* across all tested neighborhoods in all integration outputs. While we can adapt the parameter *k* in our data processing pipeline for methods that output embeddings or corrected feature spaces, it is inherent to the method for graph-based integration methods. In order to benchmark data integration in a consistent manner, we chose to use only the recommended defaults for each method. Thus, we must adapt the input for kBET rather than changing the parameters of the methods that we run to fairly evaluate batch removal across integration output formats.

The output of a graph-based integration method is a graph that encodes the biological signal that is shared across batches. Here, the graph structure, rather than the individual edge, is the important signal. Thus, to increase the number of nearest neighbors we can obtain per cell, we use the local structure in the network to increase the density of the connectivity matrix. Motivated by previous work on diffusion along kNN-graphs in scRNA-seq analysis[18,52], we achieve this by running a diffusion process on the graph. Specifically, we simulate an *N*-step diffusion process where *N* is selected to obtain a minimum of *k* non-zero connectivity per cell. This process is described by the equation:

$$M = \sum_{i=1}^{N} T^{i},$$

where *M* is the diffusion-extended connectivity matrix, and *T* is the row-normalized connectivity matrix.

The above diffusion process is performed at two points in our extended kBET metric. Firstly, we perform graph diffusion on the initial connectivity matrix of graph-based outputs before running kBET. This diffusion run ensures that we have a minimum of *k* nearest neighbors per node. Here, *k* is chosen to match the number of nearest neighbors calculated for other outputs (*k*=50). Secondly, we perform graph diffusion after the connectivity matrix is subsetted to a particular cell identity label. After subsetting, we may obtain multiple connected components in the subsetted graph, especially in poorly integrated datasets. In this setting we first assess which connected components are sufficiently large to evaluate via kBET. A sufficiently large connected component is one with at least 3**k* nodes, where *k* is chosen by the kBET default of the median number of cells per batch within the subsetted data. Note that we enforce minimum and maximum *k* thresholds of 10 and 100. Graph diffusion is performed in all sufficiently large connected components (for all integration outputs) to give a consistent number of nearest neighbors per cell. Cells in connected components that are not sufficiently large are given scores of 1, indicating poor batch integration. Furthermore, cell identity labels where fewer than 75% of cells are in sufficiently large components are given a kBET score of 1 to denote poor batch mixing.

Supplementary Note 2: Graph LISI extends LISI to graph-based integration results

In order to evaluate batch removal in data integration in a consistent manner, we need metrics that can be applied to all output formats. As corrected expression or accessibility matrices and joint embeddings can both be processed to produce integrated graphs, we specifically require metrics that work on graph structures. The only previously published metric for batch removal that works on graphs is kBET. However, to ensure a robust evaluation of batch removal, it is important to base this assessment on multiple metrics.

Local inverse simpson index (LISI)[46] scores are typically computed on nearest neighbor lists. These neighbor lists are obtained from a kNN graph algorithm computed with *k=90* neighbors. Integrated graph outputs, such as those produced by BBKNN[42] and Conos[44], return integrated graphs often with far fewer neighbors. As these methods do not also output joint embeddings, we cannot simply generate new kNN graphs to produce longer neighborhood lists. Thus, the classical LISI metric cannot be applied to integrated graph outputs.

Here, we extended the classical LISI metric to work on integrated graphs in our *graph LISI* metric. In graph LISI, we replace the distance measurement on joint embeddings with a graph distance to compute large nearest neighbor lists also when nodes only have few nearest neighbors. Specifically, we used Dijkstra's algorithm[53] on the connectivity matrix to compute shortest paths from one cell to all other cells. Thereby, the shortest path length serves as an approximation for the distance on an embedding that is typically used in kNN graph algorithms. As integrated data often form a single, connected graph, such that every cell is connected to all other cells. Using Dijkstra's algorithm, we obtain sufficiently large neighborhood sizes to compute the LISI for every cell in the largest connected component. In case there are smaller connected components for which we cannot measure graph distances to other cells, these cells belong to an outlier group, which has not been integrated well. Thus, we assign a LISI of 1 to these cells, which reflects the worst possible score. In accordance with the original LISI, we compute the median over all cells to obtain the LISI score. Finally, the LISI score is scaled in two steps to the unit interval (see **Methods**).

On corrected feature matrices (expression or accessibility) and joint embeddings, we construct a kNN graph connectivity matrix via Euclidean distances on the embedded space or on a PCA representation using the compute nearest neighbors function in Scanpy[16] (*sc.pp.neighbors*). Here, we deliberately choose `n_neighbors=15` as a basis for several reasons. Firstly, graph iLISI with `n_neighbors = 15` compared favourably to the original iLISI implementation in contrast with graph LISI using `n_neighbors = 90` on the unintegrated pancreas scenario (data not shown). Secondly, Dijkstra's algorithm runs faster the fewer neighbors are used to create the kNN graph

for corrected feature matrices and joint embeddings as the algorithm scales linearly with the number of edges[54]. Thirdly, we want to ensure a fair comparison of all output types. As mentioned above, graph-based outputs tend to have smaller neighborhood sizes. Thus, we can create similar initial conditions for graph LISI using comparable neighborhood sizes across output types.

We compared graph iLISI results to the original iLISI on two integration tasks (pancreas and human immune cells, see **Supplementary Fig. 42**). We computed graph iLISI scores on a connectivity matrix with 15 nearest neighbors for corrected feature matrix and joint embedding integration outputs, and on the integrated graph for graph-based outputs. For visualization of the graph LISI results for graph-based outputs, we used the same scores for both x- and y-axis (as the original iLISI does not apply for integrated graphs). It must be noted that we display iLISI scores after step 1 of the scaling, i.e. the shift by -1, such that the worst possible score is 0. Both scores strongly correlate for non-graph integration outputs (Pearson correlation coefficient for the pancreas task is 0.978 and 0.984 for the immune cell human task). Thus, we conclude that graph LISI is a reasonable metric to assess batch removal (as graph iLISI) and cell type preservation (as graph cLISI), respectively, on graph structures.

Supplementary Note 3: Detailed analysis of Integration tasks

3.1 Immune cells

For the immune cell atlas, we investigated two separate integration tasks: the first, considering only human samples (n=10); the second, merging human and mouse samples (n=23). In both cases, two tissues were considered: peripheral blood and bone marrow.

3.1.1 Human

In the human immune cell integration task, six challenges can be identified: (1) inter-sample variability arising from the different donors; (2) integration across single-cell protocols (10X and smart-seq2 in Villani's sample); (3) capturing consistent cell populations across tissues of origin; (4) separation of cell subtypes that are transcriptomically similar; (5) preservation of tissue-specific cell annotations as separate clusters; and (6) conservation of the trajectory of erythrocyte development across batches. Challenges (1) and (2) can be solved by removing batch effects across samples and across platforms, respectively, while preserving biological variation. Successfully solving challenge (3) can be achieved by correctly grouping cell types that are found across tissues (e.g., CD8+ and CD4+ T cells, CD20+ B cells, CD14+ and CD16+ monocytes). In challenge (4), we are interested in evaluating whether cell types that share a similar transcriptome (e.g., CD8+ and CD4+ T cells; CD14+ and CD16+ monocytes) can be recapitulated in separate subclusters. Challenge (5) concerns in particular cell annotations that are bone marrow specific, such as monocyte progenitors, erythroid progenitors, erythrocytes and CD10+ B cells. Finally, challenge (6) can be addressed by conserving the trajectory from hematopoietic stem and progenitor cells (HSPCs) via megakaryocyte progenitors and erythroid progenitors, to erythrocytes. It should be noted that we are evaluating the preservation of a global trajectory structure from two points of view: (i) by considering the whole dataset and focusing on the presence/absence of a trajectory which can be visually recognized (**Supplementary Fig. 9**); and (ii) by considering only cell types belonging to the trajectory and assessing whether the cells are placed in a continuum that is consistent with the calculated pseudotime score (**Extended Data Fig. 1,2, and Supplementary Fig. 1**). Furthermore, our trajectory metric calculates local conservation of the order of cells in the trajectory per batch compared to unintegrated data.

In the low dimensional embedding plots of the top 4 performing methods (Scanorama (embed, scaled/HVG), fastMNN (embed, unscaled/HVG), scANVI (embed, unscaled/FULL) and Harmony (embed, unscaled/HVG); **Fig. 2 b,c and Supplementary Fig. 9**), all methods appear to have resolved inter-sample and inter-platform batch effects. Moreover, most methods succeeded in capturing consistent cell populations across tissues, such as monocytes and T cells. Some

protocol-derived batch structure remained for scANVI, which tended to conserve platform-dependent substructures for full-length Smart-seq2 data (provided in the form of TPMs rather than count data) from Villani et al. in plasmacytoid dendritic cells and CD14+ and CD16+ monocytes. Given that this data violates the distribution assumption made by scANVI and scVI on the input data (negative binomial), this performance is unsurprising. Also Scanorama incorrectly separated plasmacytoid dendritic cells into two clusters that are driven by protocols, being one of the two only composed by Smart-seq2 cells from Villani. Furthermore, all methods separated monocyte-derived dendritic cells into three clusters, mainly composed by Villani's cells. As these sub-states of monocyte-derived dendritic cells are also found in the reference, this separation likely represents a biologically-relevant signal. Interestingly, in all methods but scANVI (which uses cell identity labels), one of these clusters consistently overlaps with CD16+ monocytes, giving a hint for a possible higher biological affinity of these cells to CD16+ monocytes. A second cluster appears to represent true monocyte-derived dendritic cells (across batches) while a third cluster, only composed by Villani's cells, is either kept separated, or as in the case of Harmony, incorrectly overlaps with erythrocytes. Overall, the separation of cell subtypes is successfully overcome by the four methods. All top performing methods performed well on this challenge, retaining a clear distinction between CD8+ and CD4+ T cells, and NKT and NK cells. Furthermore, a clear separation between tissue-specific cell types is achieved in all top methods.

The overall top-performing methods showed similar results on trajectory conservation: all four methods were able to conserve the order of cell identity clusters within the trajectory and scored similarly (**Fig. 2** and **Extended Data Fig. 1,2**). The good performance on trajectory conservation is expected given that the trajectory is nearly unaffected by batch even in the unintegrated data. Interestingly, optimal trajectory conservation scores are typically obtained when integrating using HVG selection and unscaled data (**Supplementary Fig. 1**). The highest trajectory conservation scores are achieved by scGen (unscaled/HVG), Scanorama (unscaled/full), fastMNN (scaled/HVG) and Seurat v3 RPCA (unscaled/HVG). Nevertheless, it is worth noting that these methods do not generally exhibit a perfect continuum of clusters in the UMAP representation, as can be seen in all cases but scGen. This observation shows the limitations of a visual confirmation of trajectories in embedding plots.

We also analyzed the poorest preprocessing combinations for the poorest performing methods (DESC (embed, scaled/HVG), SAUCIE (embed, unscaled/full), SAUCIE (gene, unscaled/full) and scGen (gene, scaled/full); **Supplementary Fig. 9**) to evaluate the result of poor data integration. Here, DESC and SAUCIE (both gene and embed) failed to integrate data between protocols, with DESC also not able to resolve inter-study batches. scGen overcorrected the data, losing the separation between different cell types (such as CD4+/CD8+ T cells, NK/NKT cells) and generating linear strands that are likely derived from insufficient model training due to insufficient data per batch. The two outputs of SAUCIE were very similar, showing that it was not successful in maintaining the separation between CD4+ and CD8+ T cells nor was able to clearly separate tissue-specific cell types, with CD14+ monocytes, erythrocytes and T cells placed in a continuum. Finally, none of the poorest performing methods conserved the trajectory of erythrocyte development (**Extended Data Fig. 1**). DESC and scGen embeddings even made

it impossible to fit a trajectory as the HSPC cluster was disconnected from the rest of the cell type clusters across which the trajectory was fit.

3.1.2 Human and mouse

Integrating mouse and human samples adds a higher level task to the challenges that characterize human samples alone: cross-species integration. In particular, this translates into two separate challenges: (1) cross-species integration inside the same tissue of origin; and (2) cross-species integration between tissues. Furthermore, we are interested in assessing if the methods are able to identify, in the low dimensional embedding plots (**Supplementary Fig. 10, 16**), a cross-species trajectory of erythrocyte differentiation. As in the case of human samples alone, we can also evaluate integration success by removal of sample and protocol batch effects while preserving tissue-specific cell identities and cell subtypes.

Considering the embedding plots of the top four performing methods (scGen (gene, unscaled/HVG), Scanorama (embed, scaled/HVG), scVI (embed, unscaled/HVG) and scANVI (embed, unscaled/HVG); **Supplementary Fig. 10**), it is interesting to notice that all methods but Scanorama were able to successfully overcome the batch effects derived from species and tissues. In fact, scGen performed very well in integrating across samples and platforms (minor problems only for monocyte-derived dendritic cells, which formed multiple clusters), as well as identifying clear clusters across tissues and species (such as monocytes, NK and NKT cells, B cells). However, it is worth noting that scGen uses cell type annotations to perform the integration, while the other three top performing methods do not. Moreover, two minor inaccuracies could be identified: CD8+ T cells standing apart from CD4+ T cells and a missing continuum between HSPCs, progenitors and erythrocytes. Good results are also achieved by scVI and scANVI, which delivered a partly successful inter-tissue and inter-species integration. Cell types such as CD4+ and CD8+ T cells, NK/NKT cells, monocytes and B cells were correctly integrated. However, integration across species failed for specific cell types such as HSPCs, erythrocytes and monocyte progenitors which showed a separation between human and mouse cells.

With regards to Scanorama, we observed a successful integration only occurring in human samples. The results on the mouse counterpart showed a strong study-related batch effect, with cell types belonging to the same tissue (bone marrow), but different studies (MCA and Dahlin), completely separated. A similar performance ranking can be observed by the overall top four methods in the trajectory analysis, when considering only the cell subset of erythroid differentiation (**Supplementary Fig. 42**). scGen, successfully removing batch effects, is able to reconstruct the trajectory across tissues and species to an extent. It is worth noting that the embedding produced by scGen shows an erroneous separation of erythrocytes into two clusters (driven by the species batch). Moreover, a good pseudotime ordering is achieved by scVI and scANVI. However, the embeddings of both methods show many human cell clusters (e.g. erythrocytes, HSPCs) that are in the correct pseudotime order, but are not integrated with their mouse counterparts. On the other hand, Scanorama failed to reconstruct the pseudotime order,

showing problems in the integration of batches. Scanorama incorrectly separated two samples from Dahlin's bone marrow from the main trajectory, assigning the latest pseudotime scores to cells belonging to all five stages of development. Interestingly, the best trajectory conservation results, achieved by Scanorama (gene, unscaled/full), Scanorama (embed, unscaled/full), scVI (embed, unscaled/full) and scANVI (embed, unscaled/full), showed a very clear reconstruction in terms of pseudotime, but poorer results in terms of batch correction, with a clear separation between species and tissues still visible (**Supplementary Fig. 16**). Indeed, Scanorama (both gene and embed) produced the top trajectory conservation scores by only fitting a trajectory to the mouse cells, as the human cells were disconnected from these clusters. This suggests that trajectory may best be reconstructed per species rather than across them. The combination of unscaled and full features proved to be the best at conserving a trajectory.
A different scenario is depicted by the four poorest-performing methods: SAUCIE (gene, unscaled/full), SAUCIE (embed, unscaled/full), DESC (embed, unscaled/HVG) and Conos (graph, unscaled/full) (**Supplementary Fig. 10**). While Conos overcorrected the data, removing batch variation due to species but also biological cell type variation, DESC output was strongly affected by multiple sources of batch effect (species, tissues and studies), leading to cells belonging to the same cell types divided into multiple subclusters. We observed a successful integration over both species and tissues only occurring in NK cells and CD8+ T cells. Yet, distances between similar cell states (e.g., CD4+ and CD8+ T cells) were lost in the embedded output, likely due to the model being based on clustering. Finally, both gene and embed outputs of SAUCIE were very similar: although the inter-platform batch effect was not resolved, we observed a partial success in the integration of mouse and human cell types such as CD4+ T cells, NK and NKT cells. Once again, integration of the two bone marrow studies (Dahlin and MCA) is missing, with a clear separation still visible for shared cell types such as neutrophils and progenitors. Moreover, SAUCIE removed most of the biological variation between specific cell types such as HSPCs, lymphocyte progenitors, monocytes, and neutrophils, belonging to the Dahlin study. Finally, smaller clusters, such as plasmacytoid dendritic cells and basophils, can no longer be detected. Trajectory structure is generally poorly conserved across the bottom performing methods, except for Conos, which exhibits a continuous transition between the cell identities involved in erythrocyte development across human and mouse data, but does not order these correctly in diffusion pseudotime (**Supplementary Fig. 42**).

3.2 Simulation 1

Simulation 1 consists of six batches designed to replicate an experiment consisting of multiple samples from a single tissue (with seven cell types), produced using different technologies. This simulation presents several challenges for integration. The simulated batches differ in number of cells (1000 - 3000), cell type proportions (0 - 35%) and counts per cell (30 - 100% of baseline). Integration methods must attempt to remove the technical differences between batches while maintaining differences between cell types and retaining cell types that are only present in some batches.

Most of the methods performed well on this task, resulting in embeddings that showed distinct clusters by cell type but little evidence of separation between batches (**Supplementary Fig. 11**). All method versions were able to improve batch correction compared to the unintegrated data and the best performing versions of methods also improved bio conservation (**Extended Data Fig. 4**). The embeddings for the best performing versions show clear separation of cell types and mixing of batches. Group 7 represented a rare cell type that is only present in two of the six batches at low proportion. While this was a challenge for some poor performing integration outputs, all top performers successfully distinguished this group from other simulated cells. Of the worst performers, MNN (unscaled/full feature) and Seurat v3 RPCA (scaled/full feature) showed some undercorrection. While they grouped cells of the same type a clear batch structure can still be seen within these groups. The other worst performer was SAUCIE (both embedding and gene; unscaled/full feature). SAUCIE's embeddings show a general separation between cell types, but this is not as clear as for the top performing methods especially for the rare cell type "Group 7". Furthermore, the least prevalent batch (Batch 6) was not integrated with the rest of the dataset.

Overall, this simulation proved to be a simple integration task such that nearly all methods regardless of pre-processing succeeded in retaining cell type structure while merging data across batches.

3.3 Simulation 2

Simulation 2 is designed to replicate a more complex experiment with a nested design and includes four batches, each of which has three subbatches. This design is analogous to a multi-center experiment where each center processes multiple batches (possibly using different technologies). In this task the between center batch effect (batch) can be expected to be larger than the batch effect between samples from the same center (subbatch). The extra level of variation presents a challenge for integration methods which must remove the batch and subbatch effects while retaining differences between cell types. An additional challenge is presented by the relatively few cells that methods can use to learn the batch effect in this task. The number of groups in Simulation 2 has been reduced to four.

As would be expected given the more complex scenario we observed a greater spread of performance on Simulation 2 compared to Simulation 1 (**Extended Data Fig. 4**). The top performing methods (Harmony, scGen, scANVI and fastMNN) were all able to improve both batch correction and bio-conservation compared to the unintegrated dataset, as were some other top-performing methods. All four of these methods produced embeddings with clearly separated cell types, however some separation of subbatches within these groups is still visible on the embeddings (apart from scGen) (**Supplementary Fig. 12**). In particular, the cells from Batch4Sub2 are separated from the rest of the cells in Group2 and Group4 in the scANVI embedding. FastMNN also shows some overlap between Group 1 and Group 3. In this task the worst performing methods showed both overcorrection and undercorrection. The embeddings

for SAUCIE (both embedding and gene output on the unscaled HVG input) and BBKNN show mixing of all batches, subbatches and groups with no clear separation in the UMAP, corresponding to low scores for both batch correction and bio conservation. In contrast, the embedding for Scanorama (gene output on the unscaled, full feature input) shows undercorrection and failure to merge cell types across batches, corresponding to very low batch correction scores. However, because cell types are still grouped together within batches the overall bio conservation scores for these integrations is similar to the mid-range performers. Other versions of Scanorama performed better but did not do as well relative to other methods on the simulations as it did on the real RNA datasets.

3.4 Pancreas

The human pancreas task consists of nine batches from six datasets. We have several challenges in the dataset: Firstly, we integrated different experimental protocols with varying sequencing depth. Data from the CEL-seq and CEL-seq2 are UMI counts, which were converted to transcript numbers through binomial statistics. Thus, the resulting values can be considered as UMI-count-like. inDrop is a UMI-based 3' biased protocol, SMARTer is a full-length protocol and was already RPKM-normalised, while SMART-Seq2 and Fluidigm C1 are full-length and highly sensitive protocols, which do not contain UMIs. Secondly, we have a nested batch effect as we consider four different donors from the inDrop dataset as separate batches, while all other datasets are treated as single batches. Thirdly, the datasets differ in data complexity, ranging from four major endocrine cell types in the SMARTer dataset to 14 different cell types in the inDrop dataset. In addition, T cells were only found in the inDrop dataset. We distinguished two subtypes of stellate cells (activated and quiescent), which should ideally be placed in close proximity to one another, but should not overlap. Likewise, the immune cell types (mast cells, macrophages, and T cells) should be placed in close proximity in the embedding plots. This collection of datasets was used in several data integration method publications to benchmark methods, which helps the reader to compare our results to the respective original literature[39,40,44–46,55].
Overall, the top performing methods integrated all batches correctly, accounting for both nested batch effects and different scales of the protocols, while separating cell types (see **Supplementary Fig. 6,13**). Seurat v3 (CCA and RPCA) was not affected by the nested batch structure, as all cells were evenly distributed within each cell type. In contrast, Harmony (embedding, unscaled/HVG) showed patches of cells from the fluidigm C1 batch in the alpha and beta cell clusters, indicating an incomplete removal of the batch effect, but a well-mixed distribution in all other cell types otherwise. In fastMNN (scaled/hvg), cells appear evenly distributed within each cell type, but we observe a small separate group of ductal cells from the celseq batch next to alpha cells. Thus, Seurat v3 visually corrected the nested batch effect better than Harmony (embedding) and fastMNN.
Examining the distribution of rare cell types (e.g., epsilon cells, T cells, macrophages, mast cells and Schwann cells), we observed several differences across the top performing methods. For instance, epsilon cells were partially merged with quiescent stellate cells in Seurat v3 CCA,

while being clearly separated in Seurat v3 RPCA, fastMNN and Harmony (embedding). Mast cells and macrophages were partially merged in Seurat v3 CCA and Harmony (embedding), while placed separately in Seurat V3 RPCA and fastMNN. The subtypes of stellate cells partially overlap in Seurat v3 CCA and Harmony (also with Schwann cells), while they are placed in close proximity but separately in all other top performing methods.

Interestingly, all top performing methods indicated transition states (*e.g.*, between alpha and acinar or ductal cells in Seurat v3; and alpha and beta cells in Harmony (embedding) and fastMNN) that were not present in the unintegrated datasets. The acinar-to-ductal transition has only been described in the context of pancreatitis and pancreatic cancer[56] all donors were reported as healthy, the indicated transitions are spurious and a result of mild overcorrection. In terms of biological variance conservation, Harmony (embedding) scores best among the top performing methods, although Scanorama performs better, but has lower batch correction performance (in 15th overall place; **Supplementary Data 1**). In general, Seurat v3 (CCA and RPCA) removed the nested batch effects, while conserving most of the strong biological signal in the pancreas datasets, while Harmony (embedding) and fastMNN accounted less well for the nested batch effect structure.

Interestingly, the poorest performing methods are predominantly deep learning approaches. Moreover, while scGen (HVG) was among the top performers (5th and 6th place, respectively), scGen (scaled/full feature) was among the worst-performing methods and had the lowest bio-conservation scores. Specifically, the UMAP for scGEN (scaled/full feature) resembles strings and a ball of wool, which is predominantly formed by beta, gamma and delta cells. This UMAP is a vivid example of the latent space of an insufficiently trained auto-encoder, which has not yet collapsed to a meaningful data distribution. In the SAUCIE UMAP plots (both gene and embedding) we observed a good batch effect removal and a broad conservation of all cell types, however the cell types mix in the embedding and are no longer easily distinguishable. Furthermore, rare cell types were merged and are no longer detectable. In contrast, LIGER created several large clusters. The most abundant endocrine cell types (i.e. alpha and beta cells) form a cluster each. However, all other clusters mix several entirely unrelated cell types (i.e. ductal, stellate and delta cells), and we observe alpha cells in all clusters. All rare cell types, except for endothelial cells, are merged with the larger clusters. This view is also reflected in the bio-conservation scores, where LIGER performed second poorest across all methods.

3.5 Lung atlas

The lung atlas integration task consists of three datasets taken from a single publication[57]. These datasets consist of 10X and Drop-seq data from lung transplants and biopsies. There are five particular challenges of the lung atlas integration task. These challenges encompass: (1) Inter-individual variation between human donors, (2) Integration of drop-seq (donor IDs B1-B4) and 10X data (donor IDs *A1-A6* and *1-6*), (3) separation of neutrophil and basal cell subtypes with specific annotation, (4) detection of rare cell types shared by few donors (ionocytes), and (5) integration across sampling types and locations. Where challenges (1), (2), (3), and (4) are solved by removing all batch effects pertaining to donor and protocol variation while retaining

detailed biological variation, it is more difficult to determine success for challenge (5). Specifically, donors with IDs *1-6* and *B1-B4* were obtained from lung transplants and tissue resections, whereas *A1-A6* donors were sampled via biopsies. While transplant samples typically probe the lung parenchyma, biopsies probe the airways. These sampling protocols result in cell type composition differences. For example, while biopsy samples contain basal 1, basal 2, ciliated and secretory cells, these are either absent (basal) or only present as minor cell populations in transplant donor data. Furthermore, while there are several cell type annotations that are present across samples (ciliated, secretory, endothelium, dendritic cells, and macrophages), these cell types can differ between sampling locations. Especially in secretory and endothelial cells it is expected that spatial location affects the transcriptome to make these cell types distinct between biopsy and transplant samples. Secretory cells from biopsy samples were originally labeled specifically as club cells, where transplant secretory cells contained no higher resolution annotation. Moreover, endothelial cells from lung parenchyma (transplant donors) will be predominantly respiratory endothelial cells that are involved in air exchange, while endothelial cells from airway walls have no such function. Thus, integration of secretory or endothelial cells into a single cluster represents a removal of biological signal. Yet, removing this signal may be intended if a low resolution overview of the data across batches is preferred. This overview may be preferable for tasks such as cell annotation transfer.

The top 4 performers in this integration task were scGen (unscaled/full), scANVI (unscaled/HVG), scVI (unscaled/HVG), and Scanorama (embed; scaled/HVG) (**Supplementary Fig. 7)**. These methods generally succeeded in integrating Drop-seq and 10X datasets, overcoming inter-individual variation, and distinguishing rare cell types such as ionocytes (**Supplementary Fig. 14)**. A central aspect that differentiates the top performing methods was the merging and preservation of cell type information from challenges (3) and (5). While all top performing methods, preserved basal cell subtypes, neutrophil subtypes were merged by scVI and Scanorama. scANVI retained some visual substructure between neutrophil subtypes but did not preserve the differences shown in the unintegrated case, whereas scGen separated neutrophil subtypes into distinct populations. Distinguishing neutrophil subtypes is particularly challenging for data integration methods as these subtypes are predominantly present in exclusive donors. Thus, methods that used cell type information (scGen and scANVI) could use this information to disentangle the biological variation from the batch separation. Considering cell identities that are shared between sampling locations, we found that no high performing integration method fully merged secretory cells from airway samples and tissue resections. While this may reflect negatively in our metrics, it also suggests a sensitivity to secretory cell subtypes. Specifically, while scVI, Scanorama, and scANVI maintained distinct clusters across spatial locations, scGen retained a very close proximity between secretory cell types across spatial locations. Overall, Scanorama tended to maintain the sampling location signal also in ciliated, endothelium, and dendritic cells, scVI in ciliated cells and partially in endothelium, and scANVI only in ciliated cells, while scGen merged all of these cell types across locations. Especially in endothelial cells, this can be regarded as removal of biological variation. In contrast, macrophages were best integrated by Scanorama (embed). These cells are predominantly found in transplant samples and thus spatial location played a lesser role here. Interestingly, all top-performing deep learning methods (scGen, scANVI, and scVI) integrated

macrophages well across platforms, but exhibited some donor substructure in the macrophage population. ScVI and Scanorama did however partially overlap the macrophage cluster with neutrophils. A quick comparison between the top 4 methods and the popular Seurat RPCA which ranked 6th shows that while batch integration performed similarly well, the conservation of biological signal is slightly better in the top performers: Seurat RPCA introduced a visual transition between macrophages and dendritic cells, and while it did conserved location in secretory cells, it also separated some drop-seq cells from the 10X cells in this cell type.
Poor integration performance varied between methods. While LIGER (unscaled/HVG) strongly overcorrected the data by mixing cell types and batches (removing most of the biological variation in the data), the poorest performing Conos and SAUCIE (embed and feature) results still showed distinguishable cell types that were merged into a continuum. Conos specifically over-integrated the data by creating a strongly connected embedding in which most cell types were overlapping and neutrophil subtypes and ionocytes are indistinguishable. SAUCIE (embed and feature) results show a similar overlapping embedding with all cell types in one winding continuum that is suggestive of insufficient model training with the default parameterizations. Interestingly, Seurat v3 CCA (scaled/HVG; ranked in bottom half, but not bottom 4; see scIB website for result) merged basal 1 and basal 2 cells, alveolar type 1 and 2 cells (otherwise noticeably distinct populations), and even merged B cells that were mainly from a single donor (B4) and exhibited substructure within this donor even in the unintegrated data. This suggests that Seurat v3 CCA performed a stronger dimensionality reduction than other methods, especially when data input is scaled, and thus also merged signals that may be variable within a single batch. Indeed, the original annotations were also generated via a Seurat analysis pipeline[57] suggesting these may have been annotated in this overcorrected low dimensional space.

3.6 Mouse brain (RNA)

The mouse brain RNA integration task consists of 4 datasets produced using different protocols. The particular challenge of this task is its size, since we have almost 1 mio. cells to integrate. Due to its size, we omitted the kBET metric for this task, as it did not scale to datasets of this size. Furthermore, mouse brain data is captured across spatial locations, and consists of both single-cell and single-nucleus RNA-seq data. While we evaluated biological label conservation only on the broad cell type labels with our metrics (**Supplementary Fig. 8**; note label-free conservation was also measured), we also investigated the spatial arrangement of cells, especially as specific subtypes of neurons, for instance, are restricted to certain regions in the brain (**Supplementary Data 7**). Furthermore, the dataset from Zeisel *et al.*[22] profiled, as the only study, cells from the Pons (PO) and the hypothalamus (HTH), while Saunders *et al.*[24], as the only study, profiled the brain regions Entopeduncular Nucleus (ENT), Globus pallidus and nucleus basalis (GP), and distinguished frontal and posterior cortex. The other two studies only provided the label ‘cortex’ (CTX). It must be noted that the spatial information in the Rosenberg *et al.*[21] dataset was inferred based on marker gene expression, which we opted to label as unknown instead (66,648 cells). Nonetheless, an ideal data integration method would remove

the batch effect and integrate the cell types resolved by their location in the brain. Here, we combined three pieces of information (dataset, cell type, and location) to visually assess the quality of integration (**Supplementary Fig. 15**). We focused on the following aspects: first, we examined how all datasets integrate and if we observe an integration of single-nucleus RNA-seq (Rosenberg dataset) and scRNA-seq datasets; then, we examine the integration of droplet-based scRNA-seq datasets (Zeisel *et al.* and Saunders *et al.*) and the Smart-seq2 dataset from Tabula Muris; next, we examined how well cell types integrated and whether rare and abundant cell types could still be distinguished; likewise, we examined unexpected spurious connections, for example, transitions from neurons to endothelial cells; finally, we considered the spatial substructure within a cell type to check if cell types match spatially (e.g., cerebellar astrocytes, neurons from the hippocampus, and neurons from the cortex).

Among the best performing methods, we find variable integration performances ranging from successful integration (scANVI embed, unscaled/HVG; and Harmony, scaled/HVG) to partial integration (ComBat and BBKNN, both unscaled/HVG). Both Combat and BBKNN integrated the Saunders and Zeisel datasets well and the Rosenberg single-nucleus RNA-seq dataset only partially. However, the Tabula Muris dataset appears to be completely separated from the other datasets in Combat, while the BBKNN integrated Tabula Muris also partially (e.g. endothelial cells from all scRNA-seq data are integrated, while other cell types from Tabula Muris are placed separate, but in close proximity of the corresponding cell types from the Zeisel and Saunders datasets). Furthermore, biological variance conservation performs well in BBKNN as rare cell types form distinct clusters and are discernible from other cell types. In contrast, Combat showed differences in the integration across cell types. ComBat integrated endothelial cells and brain pericytes from all datasets but Tabula Muris[23], while other cell types of the Tabula Muris and the Rosenberg dataset (e.g., oligodendrocytes, oligodendrocyte precursor cells and astrocytes) remained separated. We focused on the integration of Saunders and Zeisel datasets in the following. ComBat integrated rare cell types (e.g., ependymal cells, microglial cells, and macrophages), oligodendrocytes, and oligodendrocyte precursors well, although some separation between batches is still visible upon close inspection. We observe a partial preservation of regional substructure in both BBKNN and ComBat corrected data: while the complex regional structure was generally preserved in neurons, there was limited integration across batches within regional neuron clusters. For instance, cortical and hippocampal neurons from Zeisel and Saunders only partially clustered together in the UMAP, yet neurons of the cerebellum (CB) and striatum (STR) did overlap in region-specific, batch-agnostic clusters. For astrocytes, the overlap for Zeisel and Saunders batches was better, although rather than distinct regional subclusters, we observed a main astrocyte cluster in which the distribution was dominated by brain region. However, cerebellar astrocytes did separate from the main astrocyte cluster, and cells from both datasets overlapped. Brain pericytes and endothelial cells showed several subclusters in which these cell types clustered together across locations and datasets with partial cell type overlap. Based on the function and location close to blood vessels, these two cell types have highly similar transcriptional profiles, which might have led to some misannotation, and the influence of the spatial location within the brain is less pronounced.

Overall, both BBKNN and Combat created a visually partially integrated UMAP, but did not consistently preserve spatial information.

While scANVI and Harmony (HVG/scaled) successfully integrated all four datasets, some of the cell types only partially overlapped. Both scANVI and Harmony placed rare cell types as separate clusters, in which all datasets are well mixed. Exceptions are microglial cells from Tabula Muris, which cluster separately from the other datasets in the scANVI embedding, while all other cell types from this batch were placed in close proximity to the corresponding cell types from the other datasets, and olfactory ensheathing cells in the Harmony embedding, a population that is attached to the astrocyte cluster. Furthermore, brain pericytes are well integrated in Harmony while scANVI shows four separate clusters indicating a lack of integration. In contrast, scANVI integrated all oligodendrocytes, while Harmony (scaled/HVG) created two clusters, one attaching to astrocytes. Again, scANVI integrated all astrocytes, while Harmony shows two clusters: one of cerebellar (CB) astrocytes and one of astrocytes from all other regions. scANVI showed potential transitions between otherwise unrelated cell types in the UMAP embedding: microglia and neurons or endothelial cells and neurons were connected, while Harmony (scaled/HVG) showed these transitions only in a subpopulation of neurons and endothelial cells. Cell types where transitions are expected (e.g., oligodendrocytes and oligodendrocyte precursor cells) were also connected in both methods. Concerning spatial information, regions mixed equally well in both methods, and neuronal brain regions such as cortex (CTX) and striatum (STR) were correctly integrated. Overall, both Harmony (scaled/HVG) and scANVI preserved most of the rare cell type information with a tendency to overcorrect. We conclude that the batch effect across datasets, especially from the single-nucleus RNA-seq protocol, is stronger than the spatial signal in all cell types but neurons, where we observe a separation by brain region also after integration.

Among the worst performing integration runs, we also find a Scanorama (embedding and gene, unscaled/full feature) result. Both outputs failed to integrate the Tabula Muris dataset and the snRNA-seq dataset from Rosenberg *et al.*, and only partially integrated the scRNA-seq data from Zeisel *et al.* and Saunders *et al*. In the Scanorama (embedding, unscaled/full feature) output rare cell types (i.e., ependymal cells, microglial cells and macrophages) partially overlapped or formed a cluster, while oligodendrocytes from Saunders *et al.* and Zeisel *et al.* were placed next to each other, but seemed to merge with a subset of neuronal cells from the Zeisel data. In the Scanorama (gene, unscaled/full feature) output we observe a similar integration pattern as in the embedding version, but additionally oligodendrocytes and progenitors from Zeisel *et al.*, and Saunders *et al.* datasets integrated without merging with other cell types. This is reflected in a slightly higher bio conservation score for the Scanorama gene output. Furthermore, regional differences were only partially preserved. DESC (embedding, scaled/full feature) and SAUCIE showed the poorest performance in the mouse brain task. The UMAP plot of DESC integration shows more structure with many scattered cells. Only Zeisel et al. and Saunders et al. partially integrate, but the embedding does not separate major cell types such as neurons and astrocytes from these data sets. Interestingly, lowly abundant cell types, such as ependymal cells and macrophages, form separate clusters with

visually well-integrated batches. The UMAP plots of the SAUCIE integrated data show a single dense point cloud, where Tabula Muris data did not integrate at all and the other three datasets marginally overlap in most cell types, while separating slightly within the point cloud. Overall little biological variance is conserved in this embedding with cell types representing regions of an intermingled large cloud. Rare cell types can be spotted as worm-like structures at the outer rim of the UMAP plots. For the regional organisation of the data, cortex and cerebellum (CB) cluster together, but all other regions strongly mix into both cortical and CB regions. In general, similar to the top performers, the poorest performing methods showed variable batch removal performance either fully integrating snRNA-seq data fully (SAUCIE) or not at all (Scanorama, embedding and gene, unscaled/full feature). However, in the poorest integration results we observe a tendency to sacrifice cell type variation with increasing batch correction, leading to increased overlap between otherwise distinct cell types.

Overall, none of the methods created an ideal data integration and the distance from top 4 to bottom 4 methods was less obvious than in other tasks. In general, when all datasets were successfully integrated, the spatial distribution of the cells was only preserved in two of the best performing methods. In particular, spatial structure appeared to be more strongly encoded in neurons compared to other cell types, likely due to neuronal subtypes being organized in a spatial structure in the brain but not annotated in our integration task. The batch effect (i.e., protocol differences across datasets) was dominant in neurons, while less apparent in the rare cell types. A possible explanation for this effect may be the experimental handling of the cells. For example, endothelial cells and brain pericytes are small and approximately round and therefore easy to handle once they are isolated. In contrast, neurons are relatively large, fragile and have complex shapes (long, branched dendrites and axons). In the single-nucleus protocol, cell size was not a limiting factor as only nuclei were extracted from the cells. Saunders *et al.*[24] used Drop-Seq, Zeisel used the 10X Genomics protocol, and Tabula Muris FACS-sorted cells were processed in the Smart-seq2 protocol. Here, size limitations may play a role in the final data quality. Therefore, partial integration of snRNA-seq and scRNA-seq happened on non-neuronal cell types. Interestingly, scANVI and Harmony (scaled/hvg) were the only methods where we observed a successful integration of all studies in all cell types. Furthermore, BBKNN was ranked highly in particular for batch effect removal, while UMAPs indicated poorer performance than the metrics suggested (in particular for the Rosenberg snRNA-seq dataset). A contributing factor may be the lack of the kBET metric on this task, which only left 2 batch removal metrics that could be calculated for graph-based outputs (one of which BBKNN optimizes; see **Discussion**). Thus, BBKNN batch removal results in particular are likely to be less robust for the mouse brain RNA task than for other tasks. We conclude that the mouse brain integration task was a particularly difficult challenge as the batch effect was inhomogeneous within each dataset, as neurons differed more strongly than other cell types, and overall stronger than the differences due to location of the cells in all cell types but neurons. Thus, focussing on a particular cell type (such as neurons) potentially helps to obtain a cleaner integration, which matches subtypes and spatial locations more accurately.

3.7 Mouse brain ATAC tasks

The scATAC-seq data consists of 3 datasets. Each dataset was produced using a different protocol: single nucleus ATAC-seq from Fang *et al.*[26,27], single-cell combinatorial indexing from Cusanovich *et al.*[28] and 10X Chromium for the 10X dataset. We generated two different data scenarios to integrate from these three datasets, by largely changing the relative proportions of cells between datasets. We generated a large ATAC data scenario with strongly imbalanced cell populations between datasets (5%:20%:75% for 10x, Cusanovich *et al.* and Fang *et al.*, approximately) and a smaller ATAC scenario with balanced cell contributions (approximally 33%:33%:33% for 10x, Cusanovich *et al.* and Fang *et al.*). To successfully integrate scATAC-seq batches in our tasks, methods must overcome the following challenges: (1) integrate single nucleus and single-cell ATAC-seq data, (2) deal with the presence of nested batch effects in the large ATAC integration tasks, (3) preserve cell type variation, and (4) correctly isolate the cell type which is specific to only one batch.

scATAC-seq data can be collected for every position in the genome in comparison to scRNA-seq data which is only collected for genes. This poses a challenge on the selection of the features to be used as a basis for data integration. We generated integration tasks using three different types of features: peaks, windows and gene activity; all presenting their own advantages and inconveniences. The peak feature space (i.e. open chromatin peaks) is often used in the analysis of scATAC-seq data, as it provides a set of features with relevant biological information. Peaks are typically called on the pseudo-bulk data of every experiment separately. Therefore, the called peaks are potentially different per dataset and the count matrices cannot be concatenated. Thus, we built count matrices from the union of the peaks called from the different datasets independently. This provided a very large number of features, some of which were initially only present in one batch. We also constructed a window feature space, using non-overlapping sliding windows (5000bp), which generated a unique set of variables that were, by definition, identical in all datasets. However, it also provided a large number of windows which covered uninformative areas of the genome. Both peak and window feature spaces pose a problem for the scalability of integration methods that were initially built for scRNA-seq data, as they generated a large number of features (>500,000). Therefore, to reduce the feature space, we selected the top variable peaks or windows per batch. This necessary step posed a third challenge in the integration of scATAC-seq datasets: the more batches/cell-types that must be integrated, the fewer shared highly variable peaks/windows between them, hindering the integration task. We selected, per batch, the 150,000 most highly variable peaks/windows for both the large and the small integration tasks. Then, we merged batches and removed cells covered by less than 500 features after merging the data.

To circumvent the issue of the large number of dimensions in peak and window feature spaces, we also used gene activity as a proxy for gene expression. Gene activity consisted of the number of open peaks overlapping with a gene (genebody + 5kb upstream of the transcription start site)[58]. This created a small set of features (~4000) that were both consistent across

datasets and provided a data type more similar to scRNAseq count matrices, which could potentially increase the adequacy of scRNA-seq integration methods when applied to scATAC-seq data.

In summary, we generated 6 integration tasks (3 feature spaces times two data scenarios) to test 16 integration methods.

3.7.1 Small and large ATAC tasks based on 5k-bp window matrices

In the small ATAC data scenario for the window feature space, nine methods outperformed the unintegrated results in the overall score (47% of the methods). All of these methods preserved cell type variation (challenge 3) and correctly isolated Cerebellar Granule Cells, stemming from only one batch (challenge 4), although LIGER incorrectly placed them with excitatory neurons (**Supplementary Fig. 25** and **scIB website**). Seurat v3 CCA was the best-performing method in terms of high biological conservation, although it only ranked second due to a lower score for batch correction, because it did not completely mix the single-nucleus ATAC-seq dataset from Fang et al, as could be observed in the UMAP (**Supplementary Fig. 25**). LIGER ranked first, achieving the highest batch correction scores, and despite lower bio-conservation scores than Unintegrated. Among the other top methods, only Harmony succeeded at batch integration, while the others (BBKNN, Seurat v3 RPCA, scANVI, scVI, scGen and ComBat) did not completely succeed at batch integration, as batch structure could still be observed inside of every distinct cell type in the integrated object (**Supplementary Fig. 19** and **scIB website**). Interestingly, scANVI, scVI and scGen were the only machine learning methods to perform better than the unintegrated dataset. They performed very similarly on all metrics, but distinguished themselves from other machine learning methods (DESC, SAUCIE, and trVAE) with improved batch correction metrics (mainly batch ASW and graph connectivity). Conos, DESC and SAUCIE were the worst-performing methods in the small ATAC task. Although SAUCIE (both embedded and full feature) was better than the unintegrated dataset for batch correction, it had the lowest bio-conservation score because the cell-type structure was lost (**Supplementary Fig. 25**).

In the large ATAC dataset, only eight methods scaled to the larger cell numbers (from 10,761 cells in the small ATAC tasks to 84,813 cells in the large ATAC tasks) in the allocated computing resources (24 CPU cores and 384 GB of RAM for 48 hours). The methods that did not scale to large cell numbers were FastMNN, MNN, Scanorama, scANVI, scGen, scVI, Seurat v3 CCA, Seurat v3 RPCA, and trVAE. Among the methods that did scale to this large dataset, only LIGER and Harmony managed to integrate datasets between experiments and to remove the nested batch effects (challenges 1 and 2). Both methods also preserved cell type variation (challenge 3), although partially failing at keeping the Cerebellar Granular Cells isolated (challenge 4) (**Supplementary Fig. 26**). LIGER outperformed Harmony with slightly better batch correction scores, mainly due to its very strong kBet score, as well as better biological

conservation mainly due to the lower isolated label scores in Harmony (as cerebellar granule cells were partially merged with excitatory neurons; **Supplementary Fig. 20 and 26**). From the other top performing methods, ComBat and BBKNN only managed to mix the batches stemming from the same experiment, failing to integrate the batches in the 10x Genomics, Fang et *al*. and Cusanovich et *al*. with each other (challenge 1 and 2; **Supplementary Fig. 26**). BBKNN ranks below Unintegrated with a poor bio conservation score due to a low graph cLISI and isolated label F1. As in the small integration tasks, the methods with the lowest overall scores were SAUCIE, Conos and DESC.

3.7.2 Small and large ATAC tasks based on peak matrices

In the small data scenario for the peak feature space, eight methods outperformed the unintegrated dataset. The top-performing methods were BBKNN, LIGER and Seurat v3 RPCA (**Supplementary Fig. 21**), thanks to their successful tradeoff between batch effect correction and bio-conservation. However, BBKNN and Seurat v3 RPCA did not manage to completely integrate batches, compared to LIGER (challenge 1); and BBKNN also mixed the Cerebellar Granule Cells with endothelial cells, being the only top performing method to not succeed at challenge 4 (**Supplementary Fig. 27**). In terms of only the conservation of biological variance, Seurat v3 RPCA was the best integration method, however it provided only a mild batch effect correction, as could be observed in the UMAP, where batches remained fully separated and positioned next to each other per cell type (**Supplementary Fig. 27**). LIGER was the best-performing method for batch correction, which was also noticeable from the visualization (**Supplementary Fig. 27**)) as all batches were closely integrated across cell types. However, it produced an embedding with lower isolated label F1 and isolated label silhouette metrics compared to the other top methods. As in the window small data scenario, scVI, scANVI and scGen were also among the top-ranking methods. The lowest performing methods were Conos, DESC and SAUCIE, as in the small window scenario (**Supplementary Fig. 27**).

In the large ATAC dataset, only the eight methods that scaled to the large task on the windows task scaled to larger numbers of cells on peaks. Moreover, as for windows LIGER was the only method that outperformed unintegrated output in all challenges. As for windows, ComBat and BBKNN did not manage to integrate batches between datasets, but only inside datasets (**Supplementary Fig. 34** and **scIB website**). Contrary to the large windows task, BBKNN is now ranking above Unintegrated because of a relatively better biological conservation thanks to an improved graph cLISI and isolated label F1, while Harmony is now ranking lower than Unintegrated due to a lower biological conservation. Conos, SAUCIE and DESC remained the lowest performing methods.

3.7.3 Small and large ATAC tasks based on gene activity matrices

The most relevant result from both the large and small data scenarios in the gene feature space was that, for the unintegrated datasets (i.e. concatenated datasets), the gene activity feature

space had a substantially lower performance compared to the peak and window feature spaces (**Extended Data Fig. 5,6**). In particular, gene activity performed much worse in bio conservation, due to a lack of cell type diversity in all cell types, compared to the same data on peaks or windows (**Extended Data Fig. 5,6**). However, gene activity retained similar batch effect level as peaks and windows in the unintegrated embedding. The loss of biological variability did however also coincide with a lower batch variability in the integrated outputs (mean bio-conservation and batch removal scores on gene activity integrated outputs: 0.39 and 0.66; on peak integrated outputs: 0.61 and 0.50; and on window integrated outputs: 0.59 and 0.47). This suggests that removal of batch effects is easier on the gene activity space than on other feature spaces. However, as biological variance is obscured, gene activity is less well designed for scATAC-seq data analysis. This is reflected in the lower performance (in terms of overall score) for integration outputs using the gene feature space (**Extended Data Fig. 5,6**). In particular, only 21% of all the methods run on the gene feature space performed better than the peak unintegrated result in the small integration scenario, and 5% did so in the large one.

When testing the different methods on the gene feature space, we were unable to obtain a clear separation of the different cell types. However, four deep learning methods (scANVI, DESC, scVI and scGens) were able to differentiate the major cell types in the small data scenario and partially removed the technical/batch variability (**Supplementary Fig. 23**). In general, machine learning techniques (scANVI, DESC, scVI, scGen and trVAE) tended to have a high bio-conservation score. LIGER remained, like in small peaks and windows, the top method for batch correction. From the bottom of the rank, SAUCIE (both embedding and full feature), BBKNN, and fastMNN had the lowest scores for biological conservation. In contrast, BBKNN had one of the best batch correction scores (**Supplementary Fig. 23 and scIB website**).

Due to the substantial reduction of the number of features nearly all methods were able to run on the large dataset using gene activities in contrast to peaks or windows. The top three methods of the large ATAC task tended to be in line with the small ATAC task, namely scANVI, scGen, and scVI (**Supplementary Fig. 23-24**). However, in the small ATAC task DESC was ranked higher than scVI due to a higher bio-conservation score (**Supplementary Fig. 23 and scIB website**). All succeeded at partially integrating the 11 batches (challenge 1 and 2) (**Supplementary Fig. 30**). The top performing method, scANVI, performed reasonably well at batch correction and had the highest bio conservation score, as it separated all cell types in the embedding, which was not achieved by the other two top-performing methods, scGen and scVI (challenge 3; **Supplementary Fig. 30**). scANVI and scGen could identify the isolated cell type (cerebellar granule cells; challenge 4), while scVI mixed them with inhibitory neurons (**Supplementary Fig. 30**). This suggests that cell identity labels are needed to discern this cell type from others, as it is not otherwise discernible on the gene activity feature space. The bottom three methods of the large ATAC task were SAUCIE both full feature and embedding, and BBKNN Both methods of SAUCIE had low batch correction scores and low bio-conservation scores, which were also reflected in the UMAPs (**Supplementary Fig. 30**). Three methods, MNN, trVAE both embedding and full feature, did not scale from the small ATAC task to the large ATAC task (from 11,270 cells to 84,813 cells) in our benchmarking environment

(24 CPU cores and 384 GB of RAM for 48 hours) due to the large number of cells.

Based on the results from both small and large ATAC tasks in the gene features space, the top-performing methods, scANVI, scGen, and scVI, were all machine learning based methods (**Supplementary Fig. 23-24**). While scANVI and scGen were supervised methods, scVI was the best unsupervised method that performs reasonably well in both tasks. However, not all machine learning methods performed well, as trVAE and SAUCIE (both embedding and full feature) performed worse than the unintegrated datasets, while DESC performed better than the unintegrated dataset in the small ATAC task although it performed worse in the large ATAC task.

Supplementary References

1. Grün, D. *et al.* De Novo Prediction of Stem Cell Identity using Single-Cell Transcriptome Data. *Stem Cells* 1–39 (2016).
2. Muraro, M. J. *et al.* A Single-Cell Transcriptome Atlas of the Human Pancreas. *Cell Syst* **3**, 385–394.e3 (2016).
3. Lawlor, N. *et al.* Single-cell transcriptomes identify human islet cell signatures and reveal cell-type-specific expression changes in type 2 diabetes. *Genome Res.* **27**, 208–222 (2017).
4. Segerstolpe, Å. *et al.* Single-Cell Transcriptome Profiling of Human Pancreatic Islets in Health and Type 2 Diabetes. *Cell Metab.* **24**, 593–607 (2016).
5. Xin, Y. *et al.* RNA Sequencing of Single Human Islet Cells Reveals Type 2 Diabetes Genes. *Cell Metab.* **24**, 608–615 (2016).
6. Baron, M. *et al.* A Single-Cell Transcriptomic Map of the Human and Mouse Pancreas Reveals Inter- and Intra-cell Population Structure. *Cell Syst* **3**, 346–360.e4 (2016).
7. Grün, D., Kester, L. & van Oudenaarden, A. Validation of noise models for single-cell transcriptomics. *Nat. Methods* **11**, 637–640 (2014).
8. Lun, A. T. L., Bach, K. & Marioni, J. C. Pooling across cells to normalize single-cell RNA

sequencing data with many zero counts. *Genome Biol.* **17**, 75 (2016).

9. Oetjen, K. A. *et al.* Human bone marrow assessment by single-cell RNA sequencing, mass cytometry, and flow cytometry. *JCI Insight* **3**, (2018).
10. Dahlin, J. S. *et al.* A single-cell hematopoietic landscape resolves 8 lineage trajectories and defects in Kit mutant mice. *Blood* **131**, e1–e11 (2018).
11. Han, X. *et al.* Mapping the Mouse Cell Atlas by Microwell-Seq. *Cell* **173**, 1307 (2018).
12. Datasets -Single Cell Gene Expression -Official 10x Genomics Support. https://support.10xgenomics.com/single-cell-gene-expression/datasets/3.0.0/pbmc_10k_v3.
13. Freytag, S., Tian, L., Lönnstedt, I., Ng, M. & Bahlo, M. Comparison of clustering tools in R for medium-sized 10x Genomics single-cell RNA-sequencing data. *F1000Res.* **7**, 1297 (2018).
14. Sun, Z. *et al.* A Bayesian mixture model for clustering droplet-based single-cell transcriptomic data from population studies. *Nat. Commun.* **10**, 1649 (2019).
15. Villani, A.-C. *et al.* Single-cell RNA-seq reveals new types of human blood dendritic cells, monocytes, and progenitors. *Science* **356**, (2017).
16. Wolf, F. A., Angerer, P. & Theis, F. J. SCANPY: large-scale single-cell gene expression data analysis. *Genome Biol.* **19**, 15 (2018).
17. Durinck, S., Spellman, P. T., Birney, E. & Huber, W. Mapping identifiers for the integration of genomic datasets with the R/Bioconductor package biomaRt. *Nature Protocols* vol. 4 1184–1191 (2009).
18. Coifman, R. R. *et al.* Geometric diffusions as a tool for harmonic analysis and structure definition of data: diffusion maps. *Proc. Natl. Acad. Sci. U. S. A.* **102**, 7426–7431 (2005).
19. Wolf, F. A. *et al.* PAGA: graph abstraction reconciles clustering with trajectory inference through a topology preserving map of single cells. *Genome Biol.* **20**, 59 (2019).

20. Vieira Braga, F. A. *et al.* A cellular census of human lungs identifies novel cell states in health and in asthma. *Nature Medicine* vol. 25 1153–1163 (2019).

21. Rosenberg, A. B. *et al.* Single-cell profiling of the developing mouse brain and spinal cord with split-pool barcoding. *Science* **360**, 176–182 (2018).

22. Zeisel, A. *et al.* Molecular Architecture of the Mouse Nervous System. *Cell* **174**, 999–1014.e22 (2018).

23. Tabula Muris Consortium *et al.* Single-cell transcriptomics of 20 mouse organs creates a Tabula Muris. *Nature* **562**, 367–372 (2018).

24. Saunders, A. *et al.* Molecular Diversity and Specializations among the Cells of the Adult Mouse Brain. *Cell* **174**, 1015–1030.e16 (2018).

25. Single-cell RNA-seq data from Smart-seq2 sequencing of FACS sorted cells. http://dx.doi.org/10.6084/m9.figshare.5715040.v1 (2017) doi:10.6084/m9.figshare.5715040.v1.

26. Fang, R., Preissl, S., Hou, X., Lucero, J. & Wang, X. Fast and Accurate Clustering of Single Cell Epigenomes Reveals Cis-Regulatory Elements in Rare Cell Types. *bioRxiv* (2019) doi:10.1101/615179.

27. Li, Y. E. *et al.* An Atlas of Gene Regulatory Elements in Adult Mouse Cerebrum. *Cold Spring Harbor Laboratory* 2020.05.10.087585 (2020) doi:10.1101/2020.05.10.087585.

28. Cusanovich, D. A. *et al.* A Single-Cell Atlas of In Vivo Mammalian Chromatin Accessibility. *Cell* **174**, 1309–1324.e18 (2018).

29. Li, H. & Durbin, R. Fast and accurate short read alignment with Burrows–Wheeler transform. *Bioinformatics* **25**, 1754–1760 (2009).

30. Li, H. *et al.* The Sequence Alignment/Map format and SAMtools. *Bioinformatics* **25**, 2078–2079 (2009).

31. Quinlan, A. R. & Hall, I. M. BEDTools: a flexible suite of utilities for comparing genomic features. *Bioinformatics* **26**, 841–842 (2010).

32. Danese, A., Richter, M. L., Fischer, D. S., Theis, F. J. & Colomé-Tatché, M. EpiScanpy: integrated single-cell epigenomic analysis. *bioRxiv* 648097 (2019) doi:10.1101/648097.

33. Zhang, Y. *et al.* Model-based Analysis of ChIP-Seq (MACS). *Genome Biol.* **9**, 1–9 (2008).

34. Zappia, L., Phipson, B. & Oshlack, A. Splatter: simulation of single-cell RNA sequencing data. *Genome Biol.* **18**, 174 (2017).

35. Lun, A. T. L. *et al.* EmptyDrops: distinguishing cells from empty droplets in droplet-based single-cell RNA sequencing data. *Genome Biol.* **20**, 63 (2019).

36. Griffiths, J. A., Richard, A. C., Bach, K., Lun, A. T. L. & Marioni, J. C. Detection and removal of barcode swapping in single-cell RNA-seq data. *Nat. Commun.* **9**, 2667 (2018).

37. McCarthy, D. J., Campbell, K. R., Lun, A. T. L. & Wills, Q. F. Scater: pre-processing, quality control, normalization and visualization of single-cell RNA-seq data in R. *Bioinformatics* **33**, 1179–1186 (2017).

38. Johnson, W. E., Evan Johnson, W., Li, C. & Rabinovic, A. Adjusting batch effects in microarray expression data using empirical Bayes methods. *Biostatistics* vol. 8 118–127 (2007).

39. Haghverdi, L., Lun, A. T. L., Morgan, M. D. & Marioni, J. C. Batch effects in single-cell RNA-sequencing data are corrected by matching mutual nearest neighbors. *Nat. Biotechnol.* **36**, 421–427 (2018).

40. Hie, B., Bryson, B. & Berger, B. Efficient integration of heterogeneous single-cell transcriptomes using Scanorama. *Nat. Biotechnol.* **37**, 685–691 (2019).

41. Lopez, R., Regier, J., Cole, M. B., Jordan, M. I. & Yosef, N. Deep generative modeling for single-cell transcriptomics. *Nat. Methods* **15**, 1053–1058 (2018).

42. Polański, K. *et al.* BBKNN: Fast Batch Alignment of Single Cell Transcriptomes. *Bioinformatics* (2019) doi:10.1093/bioinformatics/btz625.
43. McInnes, L. & Healy, J. UMAP: Uniform Manifold Approximation and Projection for Dimension Reduction. *arXiv [stat.ML]* (2018).
44. Barkas, N. *et al.* Joint analysis of heterogeneous single-cell RNA-seq dataset collections. *Nat. Methods* **16**, 695–698 (2019).
45. Stuart, T. *et al.* Comprehensive Integration of Single-Cell Data. *Cell* **177**, 1888–1902.e21 (2019).
46. Korsunsky, I. *et al.* Fast, sensitive and accurate integration of single-cell data with Harmony. *Nat. Methods* (2019) doi:10.1038/s41592-019-0619-0.
47. Lotfollahi, M., Naghipourfar, M., Theis, F. J. & Alexander Wolf, F. Conditional out-of-sample generation for unpaired data using trVAE. *arXiv [cs.LG]* (2019).
48. Amodio, M. *et al.* Exploring single-cell data with deep multitasking neural networks. *Nat. Methods* **16**, 1139–1145 (2019).
49. Li, X. *et al.* Deep learning enables accurate clustering with batch effect removal in single-cell RNA-seq analysis. *Nat. Commun.* **11**, 2338 (2020).
50. Zappia, L., Phipson, B. & Oshlack, A. Exploring the single-cell RNA-seq analysis landscape with the scRNA-tools database. *PLoS Comput. Biol.* **14**, e1006245 (2018).
51. Büttner, M., Miao, Z., Wolf, F. A., Teichmann, S. A. & Theis, F. J. A test metric for assessing single-cell RNA-seq batch correction. *Nat. Methods* **16**, 43–49 (2019).
52. Haghverdi, L., Büttner, M., Wolf, F. A., Buettner, F. & Theis, F. J. Diffusion pseudotime robustly reconstructs lineage branching. *Nat. Methods* **13**, 845–848 (2016).
53. Dijkstra, E. W. A Note on Two Problems in Connexion with Graphs. *Numerische Mathematik* 269–271 (1959).

54. Barbehenn, M. A note on the complexity of Dijkstra's algorithm for graphs with weighted vertices. *IEEE Trans. Comput.* **47**, 263 (1998).

55. Tran, H. T. N. *et al.* A benchmark of batch-effect correction methods for single-cell RNA sequencing data. *Genome Biol.* **21**, 12 (2020).

56. Murtaugh, L. C. & Keefe, M. D. Regeneration and repair of the exocrine pancreas. *Annu. Rev. Physiol.* **77**, 229–249 (2015).

57. Vieira Braga, F. A. *et al.* A cellular census of human lungs identifies novel cell states in health and in asthma. *Nat. Med.* **25**, 1153–1163 (2019).

58. Granja, J. M., Corces, M. R., Pierce, S. E. & Bagdatli, S. T. ArchR: An integrative and scalable software package for single-cell chromatin accessibility analysis. *bioRxiv* (2020).
