# Supplementary material for: Benchmarking atlas-level data integration in single-cell genomics
Source: Nat Methods. 2021 Dec 23;19(1):41–50. doi: 10.1038/s41592-021-01336-8 (PMC8748196; doi:10.1038/s41592-021-01336-8)
Supplement: Supplementary file 2 — Reporting Summary [file 41592_2021_1336_MOESM2_ESM.pdf]

nature research

Corresponding author(s): M Colome-Tatche and FJ Theis

Last updated by author(s): Aug 23, 2021

Reporting Summary

Nature Research wishes to improve the reproducibility of the work that we publish. This form provides structure for consistency and transparency in reporting. For further information on Nature Research policies, see our Editorial Policies and the Editorial Policy Checklist.

Statistics

For all statistical analyses, confirm that the following items are present in the figure legend, table legend, main text, or Methods section.

n/a	Confirmed	
☒	☐	The exact sample size (*n*) for each experimental group/condition, given as a discrete number and unit of measurement
☒	☐	A statement on whether measurements were taken from distinct samples or whether the same sample was measured repeatedly
☐	☒	The statistical test(s) used AND whether they are one- or two-sided<br>*Only common tests should be described solely by name; describe more complex techniques in the Methods section.*
☐	☒	A description of all covariates tested
☒	☐	A description of any assumptions or corrections, such as tests of normality and adjustment for multiple comparisons
☐	☒	A full description of the statistical parameters including central tendency (e.g. means) or other basic estimates (e.g. regression coefficient) AND variation (e.g. standard deviation) or associated estimates of uncertainty (e.g. confidence intervals)
☒	☐	For null hypothesis testing, the test statistic (e.g. *F*, *t*, *r*) with confidence intervals, effect sizes, degrees of freedom and *P* value noted<br>*Give P values as exact values whenever suitable.*
☒	☐	For Bayesian analysis, information on the choice of priors and Markov chain Monte Carlo settings
☒	☐	For hierarchical and complex designs, identification of the appropriate level for tests and full reporting of outcomes
☐	☒	Estimates of effect sizes (e.g. Cohen's *d*, Pearson's *r*), indicating how they were calculated

Our web collection on statistics for biologists contains articles on many of the points above.

Software and code

Policy information about availability of computer code

Data collection	No software was used for data collection
Data analysis	scran (version 1.10.2), Scanpy (versions 1.4.4 commit bd5f862; 1.4.5 commit d69832a), biomaRt (version 2.38.0), BWA (version 0.7.17), SAMtools (version 1.10), BEDtools (version 2.29.0), HTSlib (version 1.10.2), epiScanpy (verssion 0.2.2), Splatter (version 1.10.0), R (version 3.6.1, 4.0.0), DropletUtils (version 1.6.1), Scater (version 1.14.6), mnnpy (version 0.1.9.5), batchelor (version 1.4.0), scanorama (version 1.4), scvi (version 0.6.6), bbknn (version 1.3.5), Conos (version 1.3.0), Seurat (version 3.1.1), Harmony (version 1.0), LIGER (version 0.4.2), scGen (version 1.1.5), trVAE (version 0.0.1), SAUCIE (version 2020-06-04), scikit-learn (version 0.22.1), kBET (version 0.99.6, release 4c9dafa), pandas (version 1.1.1), scipy (version 1.3.0, 1.4.1), statsmodels (version 0.11.1), rmarkdown (version 2.3), ggplot2 (version 3.3.2), reactable (version 0.2.2), drake workflow manager (version 7.12.5), renv (version 0.11.0). Custom code is available in the github repos: www.github.com/theislab/scib, www.github.com/theislab/scib-pipeline, and www.github.com/theislab/scib-reproducibility.

For manuscripts utilizing custom algorithms or software that are central to the research but not yet described in published literature, software must be made available to editors and reviewers. We strongly encourage code deposition in a community repository (e.g. GitHub). See the Nature Research guidelines for submitting code & software for further information.

Data

Policy information about availability of data

All manuscripts must include a data availability statement. This statement should provide the following information, where applicable:

- Accession codes, unique identifiers, or web links for publicly available datasets
- A list of figures that have associated raw data
- A description of any restrictions on data availability

We re-processed the following public datasets for our integration tasks: pancreas - GSE81076, GSE85241, GSE86469, GSE84133, GSE81608 (GEO), and E-

MTAB-5061 (ArrayExpress); immune cell bone marrow - GSE120221, GSE107727 (GEO); immune cell peripheral blood – 10X data from https://support.10xgenomics.com/single-cell-gene-expression/datasets/3.0.0/pbmc_10k_v3, GSE115189, GSE128066, GSE94820 (GEO); in addition to the Mouse Cell Atlas datasets of bone marrow and peripheral blood downloaded from https://figshare.com/articles/MCA_DGE_Data/5435866. For the lung integration task, the Drop-seq data was available from GEO (GSE130148), while the 10X data was obtained directly from the authors. For mouse brain (RNA), we obtained the raw count matrix for the snRNA-seq dataset from GEO (GSE110823), the annotated count matrix (10X Genomics protocol) from Zeisel et al. (http://mousebrain.org; file name L5_all.loom), and the count matrices per cell type (Drop-seq protocol) from Saunders et al. (http://dropviz.org/; DGE by Region section). FACS-sorted mouse brain tissue data (Smart-seq2 protocol, myeloid and non-myeloid cells, including the annotation file "annotations_FACS.csv") from Tabula Muris were obtained from figshare (https://figshare.com/projects/Tabula_Muris_Transcriptomic_characterization_of_20_organs_and_tissues_from_Mus_musculus_at_single_cell_resolution/27733). For the mouse brain (ATAC) integration, we used FASTQ files from Fang et al. (six samples, single nucleus ATAC-seq protocol; retrieved from http://data.nemoarchive.org/biccn/grant/cemba/ecker/chromatin/scell/raw/) and Cusanovich et al. (four samples, combinatorial indexing scATAC-seq protocol; GEO accession number GSE111586) and we retrieved fragment and index files from a 10X Genomics dataset for fresh adult mouse brain cortex (sample retrieved from https://support.10xgenomics.com/single-cell-atac/datasets/1.2.0/atac_v1_adult_brain_fresh_5k). Our re-processed versions of these datasets are publicly available as pre-processed Anndata objects on Figshare (doi: 10.6084/m9.figshare.1242096885). The output data from all metric runs are available in Supplementary Data file D1.

Field-specific reporting

Please select the one below that is the best fit for your research. If you are not sure, read the appropriate sections before making your selection.

☒ Life sciences ☐ Behavioural & social sciences ☐ Ecological, evolutionary & environmental sciences

For a reference copy of the document with all sections, see nature.com/documents/nr-reporting-summary-flat.pdf

Life sciences study design

All studies must disclose on these points even when the disclosure is negative.

Sample size	No samples were collected in this study. There was no multiple group comparison performed with a statistical test, thus no power analysis was necessary.
Data exclusions	Cells were removed from the benchmarked datasets based on standard quality control criteria following published best practices (doi: 10.15252/msb.20188746)
Replication	There are no experimental findings in this paper
Randomization	No samples were collected, thus no randomization was performed.
Blinding	There was no group allocation the investigators could be blinded to.

Reporting for specific materials, systems and methods

We require information from authors about some types of materials, experimental systems and methods used in many studies. Here, indicate whether each material, system or method listed is relevant to your study. If you are not sure if a list item applies to your research, read the appropriate section before selecting a response.

Materials & experimental systems

n/a	Involved in the study	
☒	☐	Antibodies
☒	☐	Eukaryotic cell lines
☒	☐	Palaeontology and archaeology
☒	☐	Animals and other organisms
☒	☐	Human research participants
☒	☐	Clinical data
☒	☐	Dual use research of concern

Methods

n/a	Involved in the study	
☒	☐	ChIP-seq
☒	☐	Flow cytometry
☒	☐	MRI-based neuroimaging
